# Interpreting amide proton transfer‐weighted imaging contrast between normal and tumor brain tissues using the asymmetry analysis method at 4.7 T

**DOI:** 10.1002/mrm.70041

**Published:** 2025-08-24

**Authors:** Malvika Viswanathan, Yashwant Kurmi, Xiaoyu Jiang, Junzhong Xu, Zhongliang Zu

**Affiliations:** ^1^ Vanderbilt University Institute of Imaging Science, Vanderbilt University Medical Center Nashville Tennessee USA; ^2^ Department of Biomedical Engineering Vanderbilt University Nashville Tennessee USA; ^3^ Department of Radiology and Radiological Sciences Vanderbilt University Medical Center Nashville Tennessee USA; ^4^ Department of Physics and Astronomy Vanderbilt University Nashville Tennessee USA

**Keywords:** amide proton transfer (APT), chemical exchange saturation transfer (CEST)

## Abstract

**Purpose:**

To provide a comprehensive analysis of the contributors to the amide proton transfer‐weighted (APTw) imaging signal using an asymmetry analysis method, as well as its contrast between tumors and the contralateral normal tissues at 4.7 T.

**Methods:**

First, a signal model was developed to demonstrate the dependence of APTw signal on various contributors, including water T_1_, reference signal containing direct water saturation (DS) and magnetization transfer (MT), as well as APT, amine CEST, and nuclear Overhauser enhancement (NOE) effects. Second, these effects were measured in rat brains bearing 9 L tumors, with saturation field strengths (B_1_) of 2 and 3 μT, at 4.7 T to assess their relative contributions. Specifically, the reference signal was determined using an extrapolated MT reference approach. The amine CEST effect was isolated using an auxiliary asymmetry analysis method, while the APT and NOE effects were quantified through a multiple‐pool Lorentzian fit of CEST Z‐spectra acquired at 15.2 T.

**Results:**

Our findings reveal that at 2 μT, the APT effect is comparable to the NOE/asymmetric MT effects in tumors. Whereas at 3 μT, the APT effect becomes greater than the NOE/asymmetric MT effects in tumors. At these two B_1_ levels, the contribution from the amine CEST effect cannot be ignored. APTw contrast between tumors and normal tissues primarily arises from decreased NOE/asymmetric MT effects, with an additional spillover‐dilution effect from the reduced MT effect in tumors.

**Conclusion:**

This study provides insights into the contributors to APTw signal and its contrast between tumors and normal tissues, thereby enhancing our understanding of APTw imaging.

## 1 INTRODUCTION

CEST is an MRI contrast mechanism used to detect solute molecules through chemical exchange between RF saturated solute protons and water protons.^1^, ^2^, ^3^, ^4^, ^5^ A major variation of CEST imaging is amide proton transfer (APT), which arises from proteins/peptides at approximately 3.5 ppm.^6^, ^7^ Being an indirect method for detecting solute molecules by measuring water signals, CEST/APT signals from tissues are not solely dependent on the solute‐water saturation transfer effect. They are also influenced by various confounding factors, including direct water saturation (DS) and magnetization transfer (MT) effects. Conventionally, an asymmetry analysis of the Z‐spectrum, termed the asymmetry in the magnetization transfer ratio (MTR_asym_), has been used to quantify APT effect by reducing contaminations from DS and MT effects. This method is simple for data analysis and allows for fast measurement because it does not require the acquisition of the entire Z‐spectrum with fine frequency resolution. As a result, it has been extensively used in clinical 3 T over the past decade. Previously, the MTR_asym_‐quantified APT imaging has demonstrated potential in detecting tumors,^8^, ^9^ grading tumors,^10^, ^11^, ^12^, ^13^, ^14^, ^15^, ^16^, ^17^, ^18^ distinguishing active glioma from treatment effects,^19^, ^20^, ^21^, ^22^, ^23^, ^24^, ^25^, ^26^, ^27^, ^28^ identifying genetic markers in tumors,^29^, ^30^, ^31^, ^32^, ^33^ and so forth. It presents with hyperintense signals in brain tumors compared to normal tissues, and its signal increases in more malignant tumors. In 2018, the United States (US) Food and Drug Administration (FDA) approved the use of APT imaging on Philips 3 T for clinical use in cancer diagnosis.

Despite that the MTR_asym_‐quantified APT imaging is proving useful in the clinical assessment of brain tumors, and it has become increasingly evident over the past 15 years that the signal derived from Z‐spectrum asymmetry analysis encompasses multiple contributions beyond protein/peptide amides. These additional contributions include asymmetry in the semisolid magnetization transfer (asymmetric MT) effect and macromolecular nuclear Overhauser enhancement (NOE) effect at approximately −3.5 ppm,^2^, ^5^ both of which tend to diminish in tumors and, therefore, may enhance the MTR_asym_‐quantified APT effect. Additionally, the MTR_asym_‐quantified APT effect depends on water longitudinal relaxation time (T_1w_ = 1/R_1w_) and is subject to a spillover‐dilution from the DS and MT effects, which vary significantly in tumors and scale the MTR_asym_‐quantified APT contrast between tumors and normal tissues.^34^, ^35^ Therefore, although the elevated MTR_asym_‐quantified APT effect in tumors was initially interpreted as indicative of over‐expressed proteins/peptides in highly cellular and malignant tumors,^36^, ^37^ it has been suggested that the effective application of APTw imaging in tumors might rely on a coincidental symbiotic effect of these mixed contributions.^5^ Recently, a consensus paper^38^ highlighted that the multiple contributions to the MTR_asym_‐quantified APT are dependent on the magnetic field strength (B_0_) and saturation field strength (B_1_). Consequently, MTR_asym_‐quantified APT imaging has been referred to as APT‐weighted imaging (APTw), with specific recommendations for B_1_ values in tumor applications. Understanding the relative contributions of these various effects can provide deeper insight into the molecular mechanisms that contribute to the successful application of the APTw imaging. This manuscript aims to evaluate these contributions in more detail.

Suitable B_1_ of at least 2 μT have been recommended for the APTw imaging at 3 T.^7^, ^38^ However, at such high B_1_ and low B_0_ values, resolving the APT effect and other contributions becomes challenging, complicating the evaluation of their relative magnitudes. Previously, positive APTw values at 3.5 ppm have been observed in tumors at high B_1_ values, which were used to validate that the APT effect is greater than the NOE/asymmetric MT effects.^7^, ^38^ However, this does not consider the contribution from amine. The amine CEST effect occurs at approximately 3 ppm and is in the fast exchange regime,^39^ which has a broad peak and may interfere with APT, particularly with high B_1_ values. Additionally, the DS and MT effects are significant at high B_1_ and low B_0_ values and their contaminations to the APTw contrast between tumors and normal tissues via the spillover‐dilution effect are substantial. In this article, we first provide a signal model to understand how these confounding effects and the spillover‐dilution effect impact the APTw contrast. Second, we used our previously developed auxiliary asymmetry analysis metrics^40^ to separate the contributions from the amine CEST effect, allowing us to evaluate its relative contribution. Last, we conducted experiments at 15.2 T with B_1_ of 2 μT, where the APT and NOE effects can be more distinctly resolved, to determine their relative contributions. Through these experiments, we provide an approximate estimation of the relative contributions from each effect to the APTw effect and its contrast between tumors and normal tissues.

## 2 THEORY

### 2.1 Signal model for MTR_asym_

MTR_asym_ has been defined by^1^

(1) $${\mathrm{MTR}}_{\text{asym}}\left(\Delta \omega \right)=\frac{S\left(-\Delta \omega \right)-S\left(+\Delta \omega \right)}{S_{0}},$$

where (+) indicates the labeled proton's frequency offset; (−) indicates the offset on the opposite side of the water peak; Δω is the saturation pulse frequency offset from water; S(+Δω) is the label signal and S(−Δω) is the reference signal for this asymmetry analysis; and S_0_ is the control signal acquired without saturation.

Previously, we shown that S(Δω) in a three‐pool model, including water, MT, and CEST, can be approximately descripted by Eq. ([2).^41^ Additionally, the reference signal ($S_{\mathrm{ref}}\left(\Delta \omega \right)$), which contains DS and MT effects but excludes the CEST effect, can be approximately descripted by Eq. ([3):

(2) $$\begin{array}{cc} S\left(\Delta \omega \right) & \approx \frac{S_{0}R_{1\mathrm{obs}}}{R_{\mathrm{eff}}+\frac{R_{\mathrm{ex}}^{\text{CEST}}\left(\Delta \omega \right)}{1+f_{m}}+R_{\mathrm{ex}}^{\mathrm{MT}}\left(\Delta \omega \right)}\\ & \approx \frac{S_{0}R_{1\mathrm{obs}}}{R_{\mathrm{eff}}+R_{\mathrm{ex}}^{\mathrm{MT}}\left(\Delta \omega \right)}-\frac{S_{0}R_{1\mathrm{obs}}R_{\mathrm{ex}}^{\text{CEST}}\left(\Delta \omega \right)}{{\left(R_{\mathrm{eff}}+R_{\mathrm{ex}}^{\mathrm{MT}}\left(\Delta \omega \right)\right)}^{2}\left(1+f_{m}\right)}+\cdots , \end{array}$$

(3) $$S_{\mathrm{ref}}\left(\Delta \omega \right)\approx \frac{S_{0}R_{1\mathrm{obs}}}{R_{\mathrm{eff}}+R_{\mathrm{ex}}^{\mathrm{MT}}\left(\Delta \omega \right)},$$

where $R_{1\mathrm{obs}}$ represents the observed water longitudinal relaxation rate; f_m_ is the MT pool concentration; $R_{\mathrm{eff}}\left(\Delta \omega \right)$, $R_{\mathrm{ex}}^{\text{CEST}}\left(\Delta \omega \right)$, and $R_{\mathrm{ex}}^{\mathrm{MT}}\left(\Delta \omega \right)$ denote the effective water relaxation, CEST, and MT effects in the rotating frame, respectively.^34^, ^42^, ^43^ Notably, $R_{\mathrm{ex}}^{\text{CEST}}$ depends only on the solute pool concentration, solute‐water exchange/coupling rate (k_sw_), and solute transverse relaxation rate (R_2s_), which is more specific to the solute itself and, therefore, reflects the clean CEST effect.^34^ $R_{\mathrm{ex}}^{\text{CEST}}$ can be obtained by using an apparent exchange‐dependent relaxation (AREX) metric, which inversely subtracts the label and reference signal, along with a correction of $R_{1\mathrm{obs}}$ effect.^34^

Because the symmetric MT is much larger than the asymmetric MT effect, we designate $R_{\mathrm{ex}}^{\mathrm{MT}}$ in Eqs. ([2) and ([3) to represent the symmetric MT (i.e., $R_{\mathrm{ex}}^{\mathrm{MT}}\left(-\Delta \omega \right)=R_{\mathrm{ex}}^{\mathrm{MT}}\left(+\Delta \omega \right)$) and consider the asymmetric MT as an $R_{\mathrm{ex}}^{\text{CEST}}$ effect. By replacing S(Δω) in Eq. ([2) and $S_{\mathrm{ref}}\left(\Delta \omega \right)$ in Eq. ([3) with S(+Δω) and S(−Δω) in Eq. ([1), respectively, and substituting $R_{1\mathrm{obs}}/\left(R_{\mathrm{eff}}+R_{\mathrm{ex}}^{\mathrm{MT}}\left(\Delta \omega \right)\right)$ in the second term in Eq. ([2) with $S_{\mathrm{ref}}\left(\Delta \omega \right)/S_{0}$, we deduce Eq. ([4):

(4) $$\begin{array}{cc} {\mathrm{MTR}}_{\text{asym}}\left(\Delta \omega \right) & =\frac{1}{R_{1\mathrm{obs}}}\cdot {\left(\frac{S_{\mathrm{ref}}\left(\Delta \omega \right)}{S_{0}}\right)}^{2}\frac{1}{1+f_{m}}\\ & \;\cdot \left(R_{\mathrm{ex}}^{\text{CEST}}\left(-\Delta \omega \right)-R_{\mathrm{ex}}^{\text{CEST}}\left(+\Delta \omega \right)\right)\\ & =\frac{1}{R_{1\mathrm{obs}}}\cdot R_{\mathrm{ref}}\cdot R_{\mathrm{ex}\text{\_}\text{asym}}^{\text{CEST}}\\ & =\frac{1}{R_{1\mathrm{obs}}}\cdot R_{\mathrm{ref}}\cdot {\text{AREX}}_{\text{asym}}\left(\Delta \omega \right), \end{array}$$

in which $R_{\mathrm{ex}\text{\_}\text{asym}}^{\text{CEST}}=R_{\mathrm{ex}}^{\text{CEST}}\left(-\Delta \omega \right)-R_{\mathrm{ex}}^{\text{CEST}}\left(+\Delta \omega \right)$ reflects the asymmetry analysis of CEST effect in the rotating frame. $R_{\mathrm{ex}\text{\_}\text{asym}}^{\text{CEST}}$ can be derived through an inverse asymmetry analysis, which uses the AREX metric to process the label signal and the reference signal produced using the asymmetry analysis, denoted as ${\text{AREX}}_{\text{asym}}=\left(\frac{S_{0}}{S\left(+\Delta \omega \right)}-\frac{S_{0}}{S\left(+\Delta \omega \right)}\right)R_{1\mathrm{obs}}$. In tissues, ${\text{AREX}}_{\text{asym}}$ encapsulates the summed effect of the asymmetry analysis of APT, NOE, amine CEST, and guanidinium CEST, as well as the asymmetric MT effects, in the rotating frame. Here, we termed $\frac{1}{R_{1\mathrm{obs}}}$ as the $T_{1\mathrm{obs}}$ recovery‐related contributor and $R_{\mathrm{ref}}={\left(\frac{S_{\mathrm{ref}}\left(\Delta \omega \right)}{S_{0}}\right)}^{2}\frac{1}{1+f_{m}}$ as the reference‐related contributor to ${\mathrm{MTR}}_{\text{asym}}$. $R_{\mathrm{ref}}$ contains the spillover‐dilution from DS and MT effects, which scales the ${\text{AREX}}_{\text{asym}}$ values. Figures S1 and S2 show that the approximate model in Eq. ([4) can provide a rough estimation of ${\mathrm{MTR}}_{\text{asym}}$, and that ${\mathrm{MTR}}_{\text{asym}}$ is approximately proportional to each contributor in Eq. ([4).

### 2.2 Auxiliary metric to isolate amine CEST effect from all other effects

Previously, we developed an auxiliary metric ($S_{A}$) to vary the relative contributions from different CEST pools based on their different exchange properties^40^, ^44^, ^45^, ^46^:

(5) $$S_{A}\left(\Delta \omega \right)=\frac{S_{0}}{1+\left(\frac{S_{0}}{S_{H}\left(\Delta \omega \right)}-1\right)\frac{B_{1\text{\_}L}^{2}}{B_{1\text{\_}H}^{2}}},$$

where $B_{1\text{\_}L}$ and $B_{1\text{\_}H}$ are lower and higher $B_{1}$ values, respectively, and $S_{L}$ and $S_{H}$ are the CEST signals with $B_{1\text{\_}L}$ and $B_{1\text{\_}H}$, respectively. We have shown that when $B_{1\text{\_}L}$ is 2 or 3 μT and $B_{1\text{\_}H}$ is 6 μT, the inverse asymmetry analysis of $S_{A}$ (i.e., ${\text{AREX}}_{\mathrm{aux}\text{\_}\text{asym}\text{\_}І}=\left(\frac{S_{0}}{S_{A}\left(+\Delta \omega \right)}-\frac{S_{0}}{S_{A}\left(+\Delta \omega \right)}\right)R_{1\mathrm{obs}}$) captures most contributions from the fast‐exchange pools, such as the amine, while significantly reducing contributions from the slow‐exchange pools, such as APT, NOE, and asymmetric MT found in $S_{L}$. Additionally, it eliminates the influences of DS and symmetric MT effects. Furthermore, when the inverse asymmetry analysis of $S_{A}$ is subtracted from that of $S_{L}$ (i.e., ${\text{AREX}}_{\mathrm{aux}\text{\_}\text{asym}\text{\_}\Pi }=\left(\frac{S_{0}}{S_{L}\left(+\Delta \omega \right)}-\frac{S_{0}}{S_{L}\left(+\Delta \omega \right)}-\frac{S_{0}}{S_{A}\left(+\Delta \omega \right)}+\frac{S_{0}}{S_{A}\left(+\Delta \omega \right)}\right)R_{1\mathrm{obs}}$), the results reflect most contributions from the APT/NOE/asymmetric MT, with minimal influence from the amine CEST effects found in $S_{L}$ and no influences from DS and symmetric MT effects.^40^ The guanidinium CEST effect at 2 ppm falls within the intermediate‐exchange regime, which cannot be fully separated using this method. However, its peak is relatively narrow and, therefore, contributes minimally to 3.5 ppm. Figures S3–S13 validate these two auxiliary asymmetry analyses in effectively separating the amine CEST from other effects. ${\text{AREX}}_{\mathrm{aux}\text{\_}\text{asym}\text{\_}І}$ at 3.5 ppm provides an approximate estimation of the contribution from amine to ${\text{AREX}}_{\text{asym}}$ at 3.5 ppm. Additionally, the sign of ${\text{AREX}}_{\mathrm{aux}\text{\_}\text{asym}\text{\_}\Pi }$ at 3.5 ppm indicates whether the APT dominates over NOE/asymmetric MT effects, without interference from amine. The relationship between the conventional asymmetry analysis metrics using $\omega_{1\text{\_}L}$ and the two auxiliary asymmetry analysis metrics can be descripted by,^40^

(6) $${\text{AREX}}_{\text{asym}}\left(\Delta \omega \right)={\text{AREX}}_{\mathrm{aux}\text{\_}\text{asym}\text{\_}І}\left(\Delta \omega \right)+{\text{AREX}}_{\mathrm{aux}\text{\_}\text{asym}\text{\_}\Pi }\left(\Delta \omega \right).$$

### 2.3 Signal model for ΔMTR_asym_

By inspecting Eq. ([4]), we found that $R_{1\mathrm{obs}}$, $R_{\mathrm{ref}}$ (i.e., DS and MT effects), as well as ${\text{AREX}}_{\text{asym}}$ contribute to ${\mathrm{MTR}}_{\text{asym}}$ in a multiplicative manner. In contrast, by inspecting Eq. ([6]), we found that ${\text{AREX}}_{\mathrm{aux}\text{\_}\text{asym}\text{\_}І}$ (i.e., amine CEST) and ${\text{AREX}}_{\mathrm{aux}\text{\_}\text{asym}\text{\_}\Pi }$ (i.e., APT/NOE/asymmetric MT) contribute to ${\text{AREX}}_{\text{asym}}$ in an additive manner. Therefore, we used the ratio of the multiplicative contributors between tumors and normal tissues to analyze their contributions to the APTw contrast in Eq. ([7). Meanwhile, we used the subtraction of the additive contributions between tumors and normal tissues to analyze their contributions to the APTw contrast in Eq. ([8).

(7) $$C_{{\mathrm{MTR}}_{\text{asym}}}\approx C_{R_{1\mathrm{obs}}}\cdot C_{\mathrm{ref}}\cdot C_{{\text{AREX}}_{\text{asym}}},$$

(8) $${\text{ΔAREX}}_{\text{asym}}\approx \Delta {\text{AREX}}_{\mathrm{aux}\text{\_}\text{asym}\text{\_}І}+\Delta {\text{AREX}}_{\mathrm{aux}\text{\_}\text{asym}\text{\_}\Pi },$$

where $C_{{\mathrm{MTR}}_{\text{asym}}}=\frac{{\mathrm{MTR}}_{\text{asym}\text{\_}t}}{{\mathrm{MTR}}_{\text{asym}\text{\_}n}}$, $C_{R_{1\mathrm{obs}}}=\frac{R_{1\mathrm{obs}\text{\_}n}}{R_{1\mathrm{obs}\text{\_}t}}$, $C_{\mathrm{ref}}=\frac{R_{\mathrm{ref}\text{\_}t}}{R_{\mathrm{ref}\text{\_}n}}$, $C_{{\text{AREX}}_{\text{asym}}}=\frac{{\text{AREX}}_{\text{asym}\text{\_}t}}{{\text{AREX}}_{\text{asym}\text{\_}n}}$ at 3.5 ppm, ${\text{ΔAREX}}_{\text{asym}}={\text{AREX}}_{\text{asym}\text{\_}t}-{\text{AREX}}_{\text{asym}\text{\_}n}$, $\Delta {\text{AREX}}_{\mathrm{aux}\text{\_}\text{asym}\text{\_}І}={\text{AREX}}_{\mathrm{aux}\text{\_}\text{asym}\text{\_}І\text{\_}t}-{\text{AREX}}_{\mathrm{aux}\text{\_}\text{asym}\text{\_}І\text{\_}n}$, and ${\text{AREX}}_{\mathrm{aux}\text{\_}\text{asym}\text{\_}\Pi }={\text{AREX}}_{\mathrm{aux}\text{\_}\text{asym}\text{\_}\Pi \text{\_}t}-{\text{AREX}}_{\mathrm{aux}\text{\_}\text{asym}\text{\_}\Pi \text{\_}n}$, with ‘t’ representing tumors and ‘n’ representing normal tissues. Figures S14 and S15 show that the approximate model in Eq. ([7) can provide an accurate estimation of $C_{{\mathrm{MTR}}_{\text{asym}}}$, and that $C_{{\mathrm{MTR}}_{\text{asym}}}$ is proportional to each contributor in Eq. ([7).

Based on Eq. ([7), ΔMTR_asym_ can be rewritten as:

(9) $$\begin{array}{cc} {\text{ΔMTR}}_{\text{asym}} & ={\mathrm{MTR}}_{\text{asym}\text{\_}t}-{\mathrm{MTR}}_{\text{asym}\text{\_}n}\\ & =\left(C_{{\mathrm{MTR}}_{\text{asym}}}-1\right)\cdot {\mathrm{MTR}}_{\text{asym}\text{\_}n}\\ & \approx \left(C_{R_{1\mathrm{obs}}}\cdot C_{\mathrm{ref}}\cdot {\text{AREX}}_{\text{asym}\text{\_}t}-{\text{AREX}}_{\text{asym}\text{\_}n}\right)\\ & \cdot \frac{{\mathrm{MTR}}_{\text{asym}\text{\_}n}}{{\text{AREX}}_{\text{asym}\text{\_}n}}. \end{array}$$

As a special case of Eq. ([9), when $C_{R_{1\mathrm{obs}}}$ = 1 and $C_{\mathrm{ref}}$ = 1, ${\text{ΔMTR}}_{\text{asym}}=\frac{1}{R_{1\mathrm{obs}}}\cdot R_{\mathrm{ref}}\cdot {\text{ΔAREX}}_{\text{asym}}$ according to Eq. ([4). Therefore, for the interpretation of the APTw contrast in a simpler term, we look ΔMTR_asym_ as ${\text{ΔAREX}}_{\text{asym}}$ (i.e., the variation of a combined effect from amine CEST and APT/NOE/asymmetric MT) scaled by $C_{R_{1\mathrm{obs}}}$ and $C_{\mathrm{ref}}$ (i.e., the variation of R_1obs_, DS, and MT).

## 3 METHODS

### 3.1 Animal Preparation

Rats bearing 9 L tumors were prepared by injecting 1 × 10^5^ 9 L glioblastoma cells in the brain. MRI was conducted after 2 to 3 weeks. Figure S16 displays the T_1obs_ maps from these animals, indicating tumor regions. All rats were immobilized and anesthetized with 2% to 3% isoflurane and 97% to 98% oxygen during the experiments. Respiration rate was monitored to be in a range from 40 to 70 breaths per minute. Rectal temperature was maintained at 37°C using a warm‐air feedback system. Animal procedures were approved by Vanderbilt University Medical Center.

### 3.2 MRI

CEST experiments were performed by applying a continuous wave (CW)‐CEST sequence with a 5 s saturation pulse followed by a single‐shot spin‐echo echo planar imaging and 2 s recovery time. Z‐spectra were acquired with RF frequency offset from −10 to 10 ppm, and S_0_ was obtained by setting the RF frequency offset at 250 ppm. Experiments were conducted on five rats on a Varian 4.7 T and four rats on a Bruker 15.2 T scanner. At 4.7 T, Z‐spectra with B_1_ of 0.5, 2, 3, and 6μT were acquired. T_1obs_ and f_m_ were obtained using a selective inversion recovery quantitative MT method.^47^ Water transverse relaxation rate (R_2w_ = 1/T_2w_) was obtained by using a spin‐echo sequence with a series of echo times. At 15.2 T, Z‐spectra with B_1_ of 0.5, 1, 1.5, and 2 μT were acquired. T_1obs_ was obtained using an inverse recovery sequence. All images had a matrix size 64 × 64, a FOV 30 mm × 30 mm, a slice thickness of 2 mm, and a single average.

### 3.3 In vivo validation of the signal model at 4.7 T

${\mathrm{MTR}}_{\text{asym}}$ was derived using either Eq. ([1) or Eq. ([4), whereas $C_{{\mathrm{MTR}}_{\text{asym}}}$ was derived using either ${\mathrm{MTR}}_{\text{asym}\text{\_}t}/{\mathrm{MTR}}_{\text{asym}\text{\_}n}$ or Eq. ([7). To differentiate ${\mathrm{MTR}}_{\text{asym}}$ and $C_{{\mathrm{MTR}}_{\text{asym}}}$ obtained from different methods, ${\mathrm{MTR}}_{\text{asym}}$ obtained using Eq. ([4) was called the calculated ${\mathrm{MTR}}_{\text{asym}}$, and $C_{{\mathrm{MTR}}_{\text{asym}}}$ obtained using Eq. ([7) was called the calculated $C_{{\mathrm{MTR}}_{\text{asym}}}$. ${\text{AREX}}_{\mathrm{aux}\text{\_}\text{asym}\text{\_}І}$, ${\text{AREX}}_{\text{asym}}$, and ${\text{AREX}}_{\mathrm{aux}\text{\_}\text{asym}\text{\_}\Pi }$ were obtained using methods described previously.^40^ Comparisons between ${\mathrm{MTR}}_{\text{asym}}$ and calculated ${\mathrm{MTR}}_{\text{asym}}$, and between $C_{{\mathrm{MTR}}_{\text{asym}}}$ and calculated $C_{{\mathrm{MTR}}_{\text{asym}}}$, were used to validate the signal model in Eq. ([4) and Eq. ([7).

An extrapolated MT reference (EMR) approach^48^, ^49^, ^50^ was used to obtain $S_{\mathrm{ref}}$, which was used in the calculation of Eq. ([4) and Eq. ([7). Specifically, Z‐spectra with Δω of ±10, ±8.75, ±7.5, ±0.125, and 0 ppm on 4.7 T and with B_1_ of 0.5, 2, and 3 μT were fitted to a two‐pool (water and MT) model. This model included parameters such as the coupling rate between the MT component and water (k_mw_), transverse relaxation time of the MT component (T_2m_), k_mw_f_m_T_1w_, and frequency offset of the MT component (Δ_m_).^51^ These parameters were fitted, and $S_{\mathrm{ref}}$ values at 3.5 ppm were then estimated using the fitted parameters. T_1w_/T_2w_ was obtained from the measurements. The MT was assumed to have a Lorentzian function. Tables S1 and S2 show the EMR fitting parameters and results.

### 3.4 Multiple‐pool model Lorentzian fit to separate the APT and NOE at 15.2 T

Multiple‐pool model Lorentzian fit (mfit) was used to quantify the APT and NOE effects from the Z‐spectra acquired at 15.2 T. Eq. ([10) gives the model function of the mfit method.

(10) $$\frac{S\left(\Delta \omega \right)}{S_{0}}=1-\sum_{i=1}^{N}L_{i}\left(\Delta \omega \right),$$

$L_{i}\left(∆\omega \right)=A_{i}/\left\{1+{\left(∆\omega -{∆}_{i}\right)}^{2}/{\left(0.5W_{i}\right)}^{2}\right\}$, which represents a Lorentzian line with a central frequency offset relative to water (${∆}_{i}$), peak full width at half maximum ($W_{i}$), peak amplitude ($A_{i}$), and N is the number of fitted pools. The Z‐spectra were processed using a six‐pool model Lorentzian fit, including: amide, amine/guanidine, water, NOE at −1.6 ppm,^52^, ^53^, ^54^, ^55^ NOE at −3.5 ppm, and MT. For simplicity, the NOE in this article refers to the NOE at −3.5 ppm. N was estimated by observing exchange/coupling effects on Z‐spectra acquired at 15.2 T. Table S3 shows the mfit parameters. To quantify the APT and NOE effects, reference signals were obtained by summing all Lorentzians except for the corresponding pool.^56^ Both MTR and AREX metrics, referred to as MTR_mfit_ and AREX_mfit_, respectively, were used for the quantification using the mfit method. The MTR_mfit_ and AREX_mfit_‐quantified APT and NOE values were obtained from the maximum values between 3 and 4 ppm, and between −3 and −4 ppm, respectively, on the corresponding fitted spectra.

### 3.5 Data analysis and statistics

CEST images at 4.7 T were denoised using a median filter with a window size of 3 × 3. Additionally, Z‐spectra with 2, 3, and 6 μT at 4.7 T were smoothed using the MATLAB function “smoothdata,” with a Gaussian filter and a window size of 8. This approach can effectively reduce the noise on Z‐spectra, particularly for acquisitions at high B_1_ where CEST peaks are typically broad, as demonstrated in Figure S17. The region of interest (ROI) of the tumor was defined based on the T_1obs_ map, and the ROI of the contralateral normal tissues was selected to mirror the tumor. ROI‐averaged data were used for statistical analysis. Differences in parameters between the tumors and contralateral normal tissues were assessed using Student's t‐tests.

## 4 RESULTS

### 4.1 CEST Z‐spectra and asymmetry analysis spectra demonstrating the presence of confounding effects and spillover‐dilution effect to MTR_asym_ at 4.7 T

**FIGURE 1:**
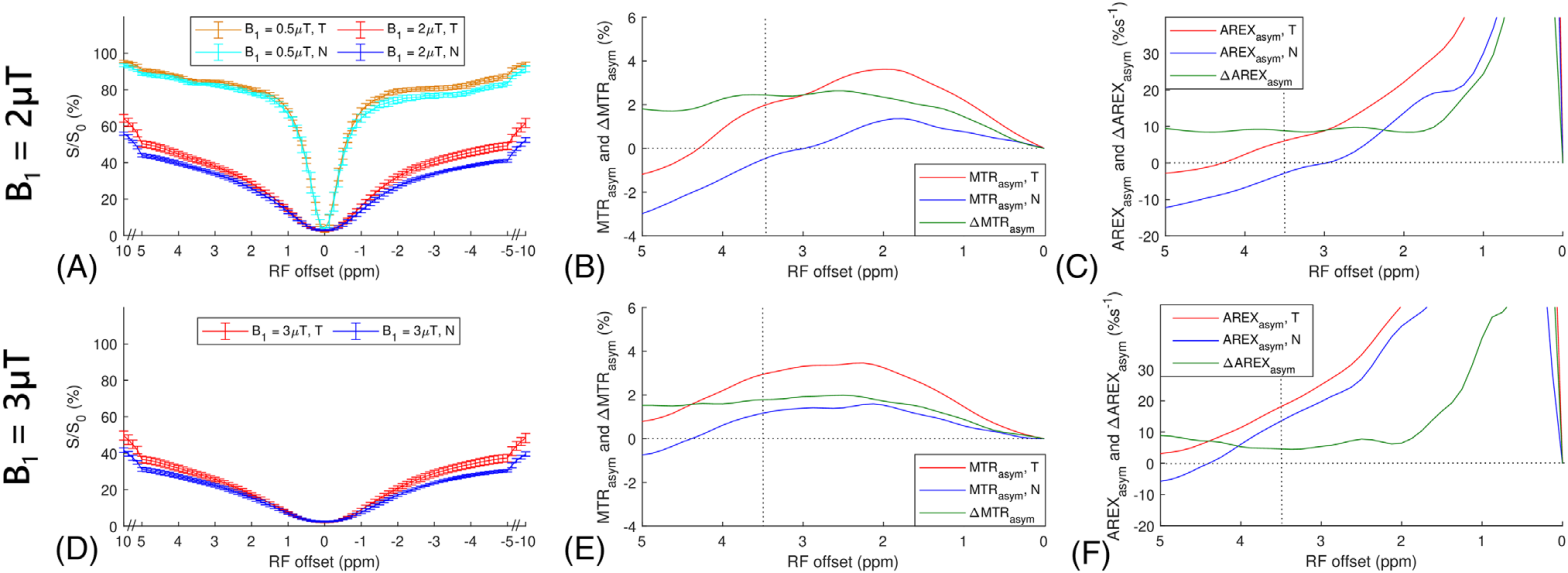
(A,D) Shows the mean and SD of the CEST Z‐spectra from the tumors (T) and contralateral normal tissues (N) in rat brains with B_1_ of 2 μT and 3 μT, respectively, at 4.7 T. (B,E) Show the corresponding averaged ${\mathrm{MTR}}_{\text{asym}}$ and ${\text{ΔMTR}}_{\text{asym}}$ spectra with B_1_ of 2 μT and 3 μT, respectively. (C,F) Show the corresponding averaged ${\text{AREX}}_{\text{asym}}$ and ${\text{ΔAREX}}_{\text{asym}}$ spectra with B_1_ of 2 μT and 3 μT, respectively. The averaged CEST Z‐spectra with B_1_ of 0.5 μT are also plotted in (A). Dotted lines in (B,E,C,F) represent the ${\mathrm{MTR}}_{\text{asym}}$, ${\text{ΔMTR}}_{\text{asym}}$, ${\text{AREX}}_{\text{asym}}$, and ${\text{ΔAREX}}_{\text{asym}}$ values of 0% and 0%s^−1^, respectively, with RF offset of 3.5 ppm.

Figure 1A,D illustrate the Z‐spectra from tumors and normal tissues in the five rats, using B_1_ of 2 and 3 μT, respectively, at 4.7 T. Figure 1A also shows the Z‐spectra with B_1_ of 0.5 μT to demonstrate the presence of the APT peak that cannot be discernibly observed at higher B_1_ values. Figure 1B,E display the corresponding ${\mathrm{MTR}}_{\text{asym}}$ and ${\text{ΔMTR}}_{\text{asym}}$ spectra with B_1_ of 2 and 3 μT, respectively. The positive ${\mathrm{MTR}}_{\text{asym}}$ at 3.5 ppm in tumors, as well as the shapes of the ${\mathrm{MTR}}_{\text{asym}}$ and ${\text{ΔMTR}}_{\text{asym}}$ spectra, with these two B_1_ values, align with earlier findings at both 4.7 T^7^, ^57^ and 3 T.^58^ Notably, although there is a small peak at 3.5 ppm in both the ${\mathrm{MTR}}_{\text{asym}}$ and ${\text{AREX}}_{\text{asym}}$ spectra, it overlays a sloping baseline. This sloping baseline belongs to a broader peak that centers at approximately 2 ppm in the ${\mathrm{MTR}}_{\text{asym}}$ spectra and closer to water in the ${\text{AREX}}_{\text{asym}}$ spectra, potentially indicating the presence of the amine/guanidinium CEST effects. Additionally, the ${\mathrm{MTR}}_{\text{asym}}$ values near 5 ppm with B_1_ of 2 μT are negative, suggesting the presence of NOE/asymmetric MT effects. Consequently, ${\mathrm{MTR}}_{\text{asym}}$ at 3.5 ppm represents a combined contribution from APT, amine/guanidinium CEST, and NOE/asymmetric MT effects, which aligns with the previous discussion.^5^, ^38^ Figure S18 demonstrates ${\mathrm{MTR}}_{\text{asym}}$ and ${\text{AREX}}_{\text{asym}}$ spectra with/without amine/guanidinium CEST or NOE/asymmetric MT effects to support this analysis.

Figure 1C,F presents the corresponding ${\text{AREX}}_{\text{asym}}$ and ${\text{ΔAREX}}_{\text{asym}}$ spectra with B_1_ of 2 and 3 μT, respectively. Notably, although ${\text{ΔMTR}}_{\text{asym}}$ at 3.5 ppm in Figure 1B,E are higher than those farther from water, previously used to validate the dominance of the variation of APT over the variation of NOE/asymmetric MT in tumors,^57^ ${\text{ΔAREX}}_{\text{asym}}$ at 3.5 ppm is comparable to or even lower than those farther from water. This indicates that higher ${\text{ΔMTR}}_{\text{asym}}$ at 3.5 ppm may not exclusively represent an APT peak, but could also be attributed to the spillover‐dilution effect from varied DS and MT effects in tumors. Figures S19 and S20 demonstrate the presence of a peak at 3.5 ppm in the ${\text{ΔMTR}}_{\text{asym}}$ spectra, but not in the ${\text{ΔAREX}}_{\text{asym}}$ spectra, when T_2w_ and MT effect were varied while keeping APT effect constant. In addition, Figure S21 show the presence of a peak at 3.5 ppm in the ${\text{ΔMTR}}_{\text{asym}}$ spectra, but a lower value at 3.5 ppm than those farther from water in the ${\text{ΔAREX}}_{\text{asym}}$ spectra, when both the NOE (−3.5) and asymmetric MT effects were varied while keeping APT effect constant. These simulations confirm the observations from animals supporting this analysis.

### 4.2 Signal model to evaluate the multiplicative contributors (i.e., T_1obs_ and MT) to MTR_asym_ contrast between tumors and normal tissues at 4.7 T

**FIGURE 2:**
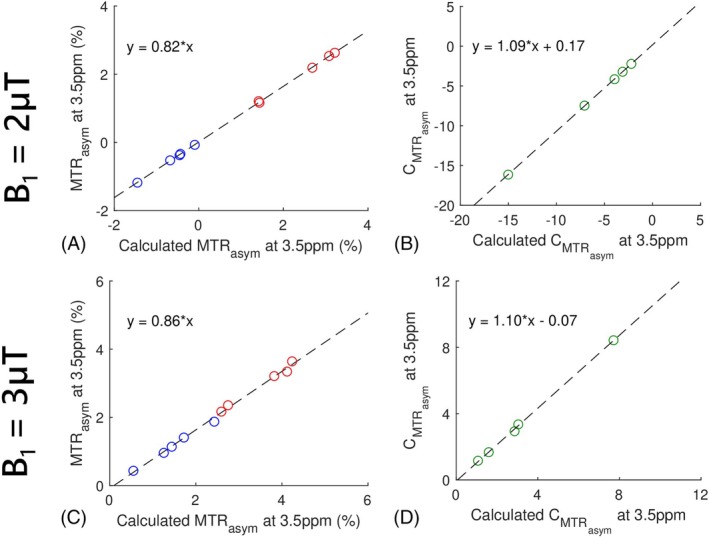
Scatter plots between the ${\mathrm{MTR}}_{\text{asym}}$ values at 3.5 ppm obtained using Eq. ([1) and the calculated ${\mathrm{MTR}}_{\text{asym}}$ values at 3.5 ppm using Eq. ([4) (A,C), as well as between the $C_{{\mathrm{MTR}}_{\text{asym}}}$ values at 3.5 ppm obtained using ${\mathrm{MTR}}_{\text{asym}\text{\_}t}/{\mathrm{MTR}}_{\text{asym}\text{\_}n}$ and the calculated $C_{{\mathrm{MTR}}_{\text{asym}}}$ values at 3.5 ppm using Eq. ([7) (B,D), from the tumors and contralateral normal tissues in the five rat brains with the B_1_ of 2 μT (A,B) and 3 μT (C,D), respectively, at 4.7 T. The dashed black lines represent the linear regression of all data points in each subfigure. The red circles represent the mean values of each tumor, the blue circles represent the mean values of each region of interest (ROI) of contralateral normal tissue, and the green circles denote the mean values of each corresponding tumor and contralateral normal tissue.

We first provide the in vivo validation of the signal model in Eq. ([4) and Eq. ([7) in Figure 2. This figure includes the scatter plots comparing the ${\mathrm{MTR}}_{\text{asym}}$ at 3.5 ppm obtained using Eq. ([1) and calculated ${\mathrm{MTR}}_{\text{asym}}$ at 3.5 ppm using Eq. ([4), as well as comparing $C_{{\mathrm{MTR}}_{\text{asym}}}$ at 3.5 ppm obtained using ${\mathrm{MTR}}_{\text{asym}\text{\_}t}/{\mathrm{MTR}}_{\text{asym}\text{\_}n}$ and calculated $C_{{\mathrm{MTR}}_{\text{asym}}}$ at 3.5 ppm using Eq. ([7), from the tumors and normal tissues in the five rats with B_1_ of 2 and 3 μT, respectively. The linear dependence of the ${\mathrm{MTR}}_{\text{asym}}$ and $C_{{\mathrm{MTR}}_{\text{asym}}}$ on the calculated ${\mathrm{MTR}}_{\text{asym}}$ and calculated $C_{{\mathrm{MTR}}_{\text{asym}}}$ further validate the approximate model in Eqs. ([4) and ([7).

**FIGURE 3:**
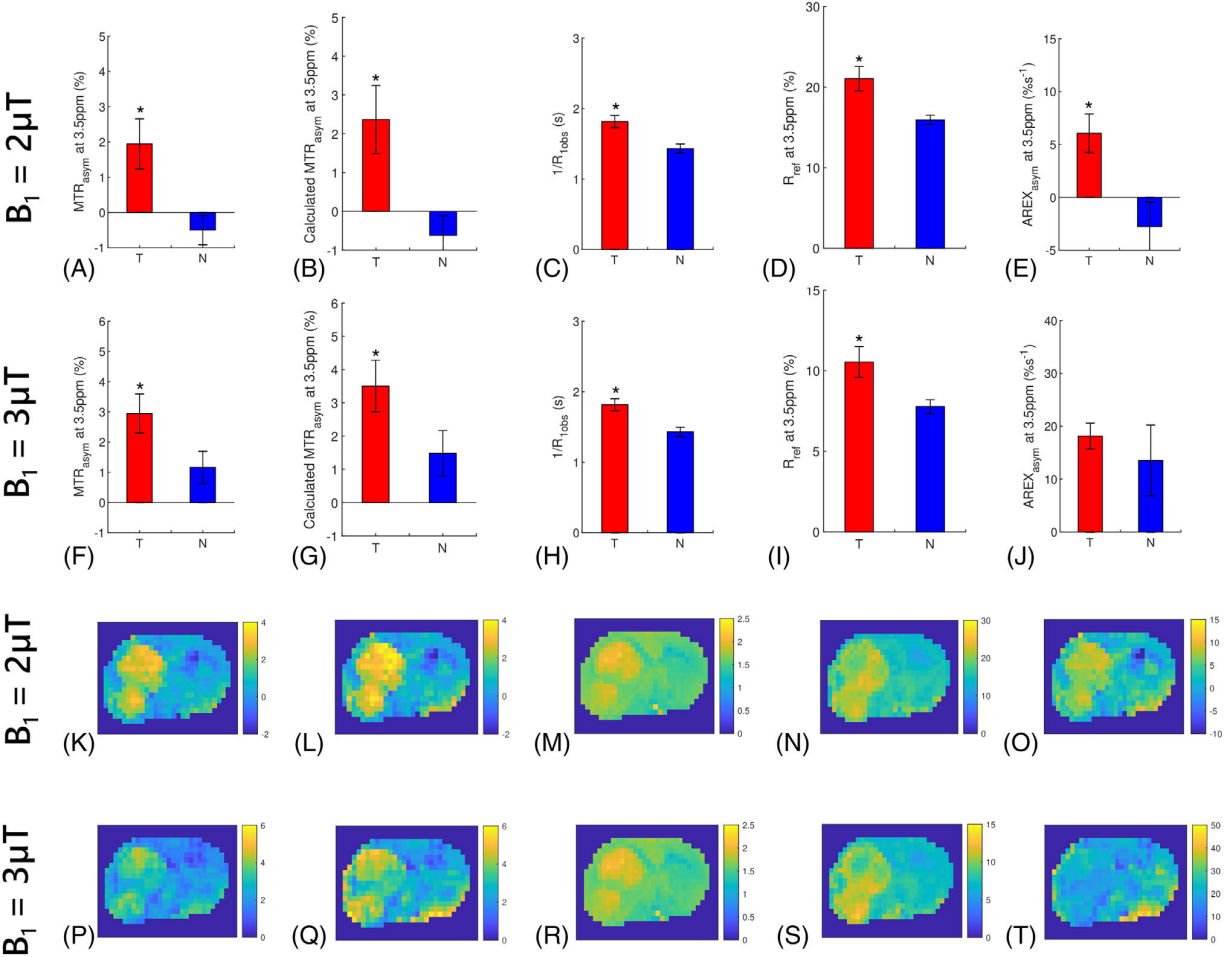
Statistical differences of the ${\mathrm{MTR}}_{\text{asym}}$ values at 3.5 ppm (A,F), calculated ${\mathrm{MTR}}_{\text{asym}}$ values at 3.5 ppm (B,G), $1/R_{1\mathrm{obs}}$ (C,H), $R_{\mathrm{ref}}$ at 3.5 ppm (D,I), and ${\text{AREX}}_{\text{asym}}$ values at 3.5 ppm (E,J) between the tumors (T) and contralateral normal tissues (N) with B_1_ of 2 μT and 3 μT at 4.7 T. The corresponding maps of the ${\mathrm{MTR}}_{\text{asym}}$ values at 3.5 ppm (K,P), calculated ${\mathrm{MTR}}_{\text{asym}}$ values at 3.5 ppm (L,Q), $1/R_{1\mathrm{obs}}$ (M,R), $R_{\mathrm{ref}}$ at 3.5 ppm (N,S), and ${\text{AREX}}_{\text{asym}}$ values at 3.5 ppm (O,T) with B_1_ of 2 and 3 μT from a representative rat at 4.7 T. (*p* < 0.05).

**FIGURE 4:**
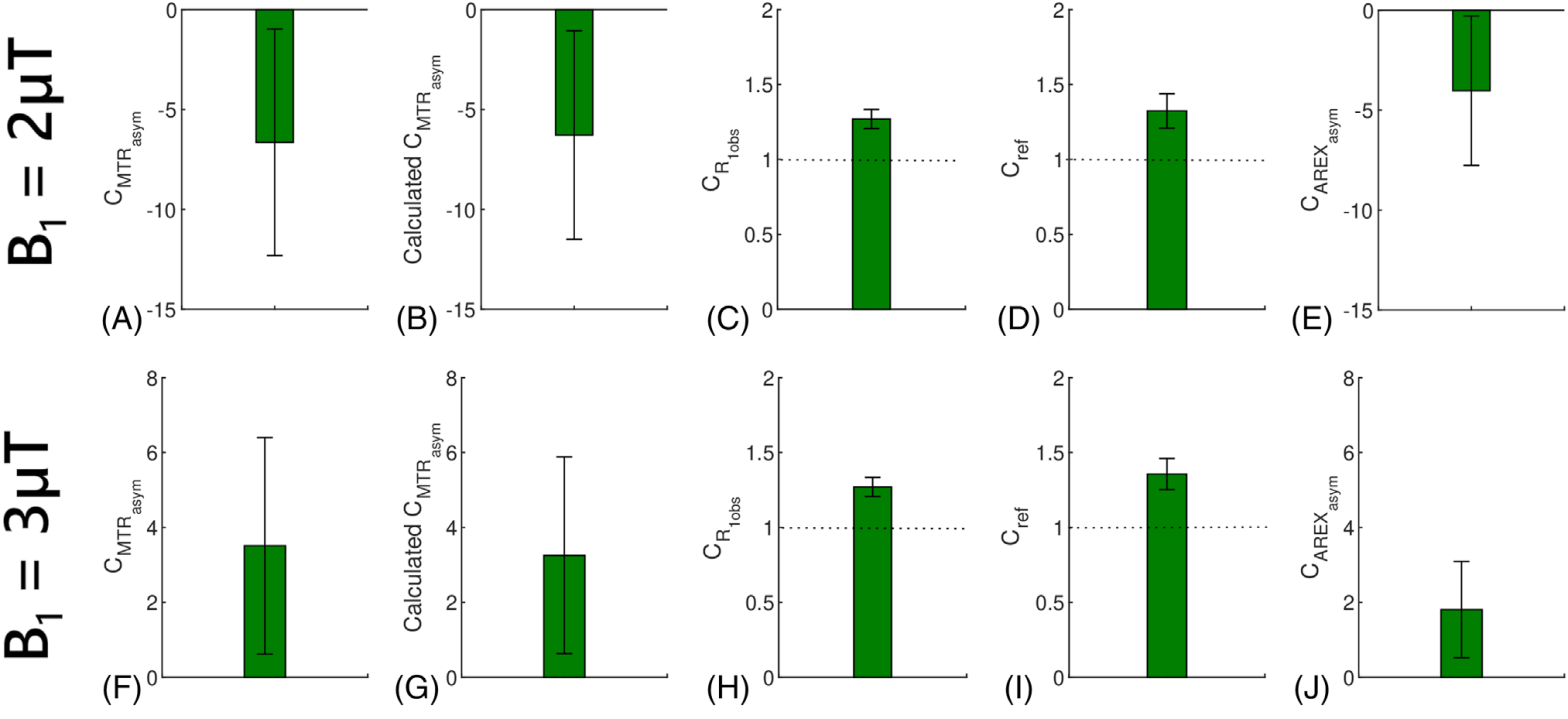
Mean and SD of $C_{{\mathrm{MTR}}_{\text{asym}}}$ (A,F), calculated $C_{{\mathrm{MTR}}_{\text{asym}}}$ (B,G), $C_{R_{1\mathrm{obs}}}$ (C,H), $C_{\mathrm{ref}}$ (D,I), and $\Delta {\text{AREX}}_{\text{asym}}$ (E,J) values with B_1_ of 2 μT and 3 μT at 4.7 T.

Figure 3 illustrates the statistical differences of the ${\mathrm{MTR}}_{\text{asym}}$ at 3.5 ppm, calculated ${\mathrm{MTR}}_{\text{asym}}$ at 3.5 ppm, $T_{1\mathrm{obs}}$ recovery‐related contributor, reference‐related contributor, and ${\text{AREX}}_{\text{asym}}$ at 3.5 ppm between the tumors and normal tissues, as well as their corresponding maps from a representative rat. Figure 4 presents the mean and SD of $C_{{\mathrm{MTR}}_{\text{asym}}}$, calculated $C_{{\mathrm{MTR}}_{\text{asym}}}$, $C_{R_{1\mathrm{obs}}}$, $C_{\mathrm{ref}}$, and $C_{{\text{AREX}}_{\text{asym}}}$ values. Notably, the calculated ${\mathrm{MTR}}_{\text{asym}}$ and calculated $C_{{\mathrm{MTR}}_{\text{asym}}}$ values closely approximate the ${\mathrm{MTR}}_{\text{asym}}$ and $C_{{\mathrm{MTR}}_{\text{asym}}}$ values, albeit with some bias. Significant differences are observed between tumors and normal tissues across all metrics, except for the ${\text{AREX}}_{\text{asym}}$ at 3.5 ppm using B_1_ of 3 μT. Despite the lack of significantdifference, the mean value of this metric is still higher in tumors compared to normal tissues. Therefore, according to Eq. ([7), this result suggests that the $T_{1\mathrm{obs}}$ recovery‐related contributor, reference‐related contributor, and ${\text{AREX}}_{\text{asym}}$ significantly influence the ${\mathrm{MTR}}_{\text{asym}}$ contrast between tumors and normal tissues. Moreover, $C_{{\mathrm{MTR}}_{\text{asym}}}$ values are greater than $C_{{\text{AREX}}_{\text{asym}}}$ values, because of the enhancement effect from $C_{R_{1\mathrm{obs}}}$ and $C_{\mathrm{ref}}$. Specifically, $C_{R_{1\mathrm{obs}}}$ and $C_{\mathrm{ref}}$ are 1.27 ± 0.06 and 1.32 ± 0.12, respectively, for B_1_ of 2 μT. Similarly, these values are 1.27 ± 0.06 and 1.36 ± 0.10, respectively, for B_1_ of 3 μT. The combined effect from both the $T_{1\mathrm{obs}}$ recovery‐related and the reference‐related contributors (i.e., $C_{R_{1\mathrm{obs}}}\cdot C_{\mathrm{ref}}$) enhances the ${\mathrm{MTR}}_{\text{asym}}$ contrast between tumor and normal tissues by approximately 1.68 and 1.73 times for B_1_ of 2 and 3 μT, respectively.

In our previous analysis using a two‐pool (solute and water) model, we found that ${\mathrm{MTR}}_{\text{asym}}$ is roughly insensitive to $T_{1\mathrm{obs}}$ when B_1_ is high.^59^ This is attributed to the presence of a $T_{1\mathrm{obs}}$‐related saturation effect in the reference signal (see Supporting Information discussion), which impacts DS and has an opposite influence on ${\mathrm{MTR}}_{\text{asym}}$. As the B_1_ value increases, this effect also intensifies, effectively nullifying the $T_{1\mathrm{obs}}$ recovery effect. This phenomenon can also be observed from our three‐pool (solute, water, and MT) model simulations in Figures S19 and S20, which show the insensitivity of ${\mathrm{MTR}}_{\text{asym}}$ to $T_{1\mathrm{obs}}$. Additionally, R_2w_ values from tumors and normal tissues were measured to be 14.5 ± 0.9 s^−1^ and 17.1 ± 0.4 s^−1^, respectively. These comparable values suggest that any variations in DS attributable to changes in R_2w_ in tumors are likely to be minimal. Therefore, these findings collectively suggest that MT is a primary element that contributes to $C_{R_{1\mathrm{obs}}}\cdot C_{\mathrm{ref}}$, which scales the ${\mathrm{MTR}}_{\text{asym}}$ contrast between tumors and normal tissues.

### 4.3 Auxiliary asymmetry analysis to evaluate the additive contributor (i.e., amine) to MTR_asym_ at 4.7 T

**FIGURE 5:**
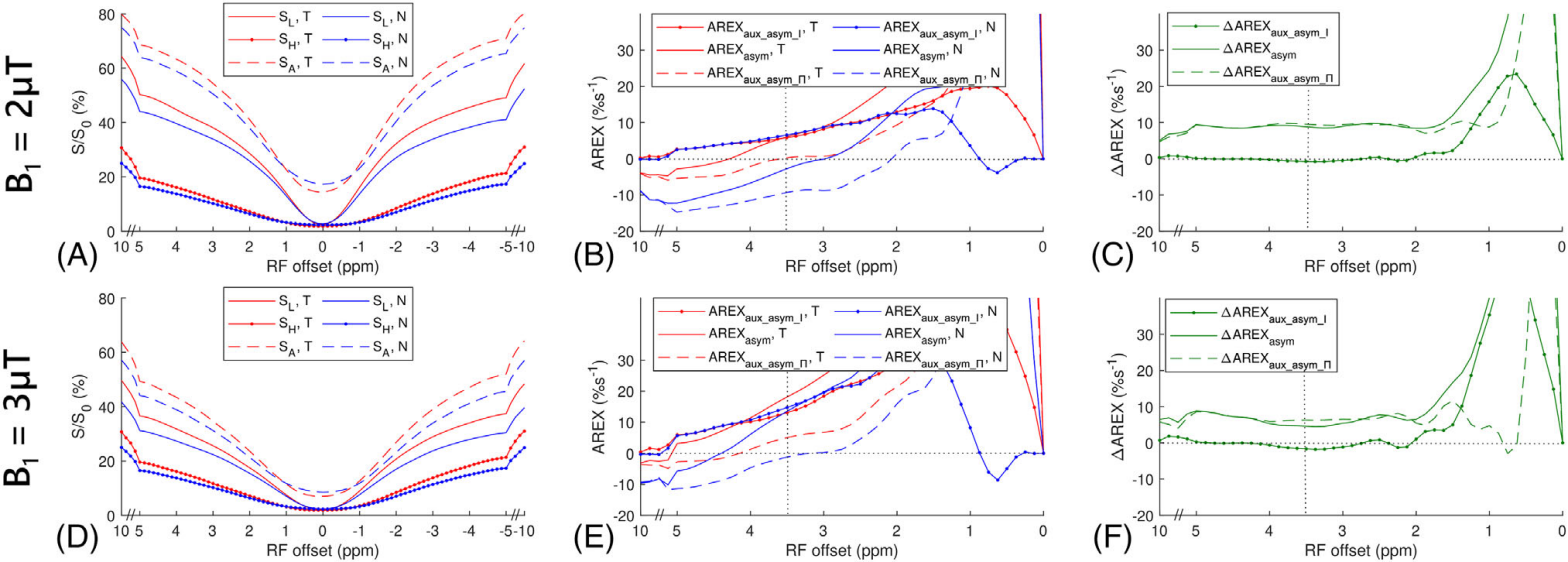
(A,D) Show the averaged CEST Z‐spectra from the tumors (T) and contralateral normal tissues (N) in rat brains with low B_1_ value (S_L_) and high B_1_ value (S_H_), along with the corresponding auxiliary Z‐spectra (S_A_) with $B_{1\text{\_}L}$ of 2 μT and 3 μT, respectively, at 4.7 T. (B,E) Show the corresponding averaged ${\text{AREX}}_{\mathrm{aux}\text{\_}\text{asym}\text{\_}І}$, ${\text{AREX}}_{\text{asym}}$, and ${\text{AREX}}_{\mathrm{aux}\text{\_}\text{asym}\text{\_}\Pi }$ spectra with $B_{1\text{\_}L}$ of 2 and 3 μT, respectively. (C,F) Show the corresponding averaged ${\text{ΔAREX}}_{\mathrm{aux}\text{\_}\text{asym}\text{\_}І}$, $\Delta {\text{AREX}}_{\text{asym}}$, and ${\text{ΔAREX}}_{\mathrm{aux}\text{\_}\text{asym}\text{\_}\Pi }$ spectra with $B_{1\text{\_}L}$ of 2 μT and 3 μT, respectively. Dotted lines in (B,C,E,F) represent the AREX values or ΔAREX values of 0%s^−1^ and RF offset of 3.5 ppm.

Figure 5A,D illustrate the Z‐spectra (S_L_ and S_H_) from the tumors and normal tissues, along with the corresponding S_A_, with $B_{1\text{\_}L}$ of 2 and 3 μT, respectively. Figure 5B,E display the corresponding ${\text{AREX}}_{\mathrm{aux}\text{\_}\text{asym}\text{\_}І}$, ${\text{AREX}}_{\text{asym}}$, and ${\text{AREX}}_{\mathrm{aux}\text{\_}\text{asym}\text{\_}\Pi }$ spectra. Similar to the simulations in Figure S3, the ${\text{AREX}}_{\mathrm{aux}\text{\_}\text{asym}\text{\_}І}$ spectra exhibit a gentler slope compared to the ${\text{AREX}}_{\text{asym}}$ spectra, and their values at 5 ppm transition from negative to positive or become higher than ${\text{AREX}}_{\text{asym}}$ at 5 ppm. This change is attributed to the capability of the ${\text{AREX}}_{\mathrm{aux}\text{\_}\text{asym}\text{\_}І}$ metric to reduce the contribution from the NOE/asymmetric MT effects. Additionally, the small peak at 3.5 pm in both ${\text{AREX}}_{\text{asym}}$ and ${\text{AREX}}_{\mathrm{aux}\text{\_}\text{asym}\text{\_}\Pi }$ spectra become weaker in the ${\text{AREX}}_{\mathrm{aux}\text{\_}\text{asym}\text{\_}І}$ spectra. This is because of the capability of the ${\text{AREX}}_{\mathrm{aux}\text{\_}\text{asym}\text{\_}І}$ metric to reduce APT. On comparison of the ${\text{AREX}}_{\mathrm{aux}\text{\_}\text{asym}\text{\_}\Pi }$ with ${\text{AREX}}_{\text{asym}}$ spectra, a significant reduction in the ${\text{AREX}}_{\mathrm{aux}\text{\_}\text{asym}\text{\_}\Pi }$ spectra was observed, suggesting that the amine CEST significantly contribute to ${\text{AREX}}_{\text{asym}}$ in both tumors and normal tissues.

**FIGURE 6:**
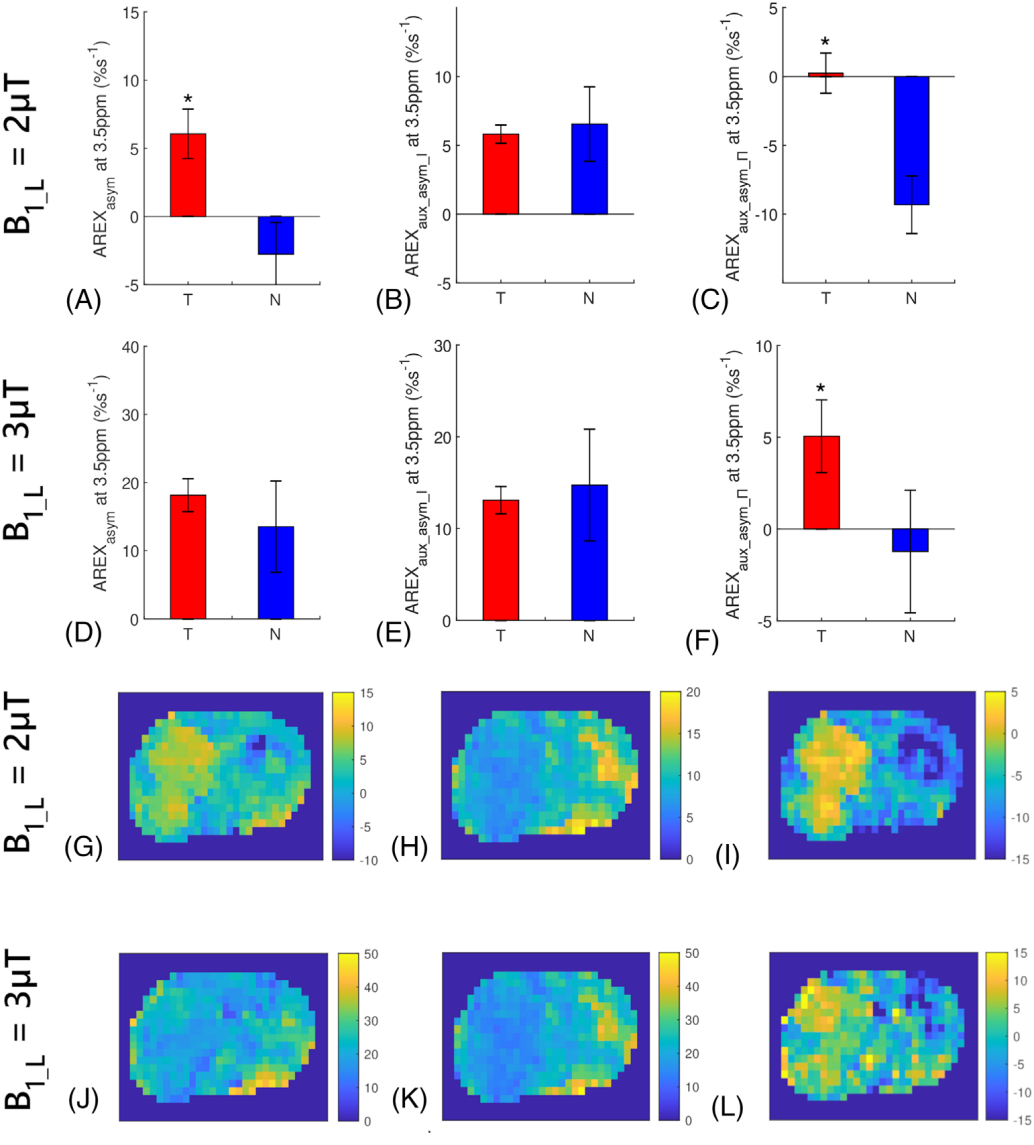
Statistical differences of the ${\text{AREX}}_{\text{asym}}$ values at 3.5 ppm (A,D), ${\text{AREX}}_{\mathrm{aux}\text{\_}\text{asym}\text{\_}І}$ values at 3.5 ppm (B,E), and ${\text{AREX}}_{\mathrm{aux}\text{\_}\text{asym}\text{\_}\Pi }$ values at 3.5 ppm (C,F) between the tumors (T) and contralateral normal tissues (N) with B_1_ of 2 μT and 3 μT at 4.7 T. The corresponding maps of the ${\text{AREX}}_{\text{asym}}$ values at 3.5 ppm (G,J), ${\text{AREX}}_{\mathrm{aux}\text{\_}\text{asym}\text{\_}І}$ values at 3.5 ppm (H,K), and ${\text{AREX}}_{\mathrm{aux}\text{\_}\text{asym}\text{\_}\Pi }$ values at 3.5 ppm (I,L) with B_1_ of 2 μT and 3 μT from a representative rat at 4.7 T. (*p* < 0.05).

Figure 5C,F displays the $\Delta {\text{AREX}}_{\mathrm{aux}\text{\_}\text{asym}\text{\_}І}$, ${\text{ΔAREX}}_{\text{asym}}$, and $\Delta {\text{AREX}}_{\mathrm{aux}\text{\_}\text{asym}\text{\_}\Pi }$ spectra. Both the ${\text{ΔAREX}}_{\text{asym}}$ and ${\text{ΔAREX}}_{\mathrm{aux}\text{\_}\text{asym}\text{\_}\Pi }$ spectra exhibit broad profiles extending up to 10 ppm without distinct peaks, suggesting a dominant contribution from the NOE/asymmetric MT and a minimal influence from the APT effect. Additionally, the$\;\;{\text{ΔAREX}}_{\mathrm{aux}\text{\_}\text{asym}\text{\_}І}$ spectra are much smaller than both the ${\text{ΔAREX}}_{\text{asym}}$ and ${\text{ΔAREX}}_{\mathrm{aux}\text{\_}\text{asym}\text{\_}\Pi }$ spectra, and they approach zero. In contrast, the ${\text{ΔAREX}}_{\mathrm{aux}\text{\_}\text{asym}\text{\_}\Pi }$ spectra are comparable to ${\text{ΔAREX}}_{\text{asym}}$ spectra. Figure 6 shows the statistical differences of ${\text{AREX}}_{\text{asym}}$, ${\text{AREX}}_{\mathrm{aux}\text{\_}\text{asym}\text{\_}І}$, and ${\text{AREX}}_{\mathrm{aux}\text{\_}\text{asym}\text{\_}\Pi }$ at 3.5 ppm between the tumors and normal tissues, as well as their corresponding maps from a representative rat. Significant differences in ${\text{AREX}}_{\mathrm{aux}\text{\_}\text{asym}\text{\_}\Pi }$ at 3.5 ppm were observed between tumors and normal tissues, whereas no significant differences were found in ${\text{AREX}}_{\mathrm{aux}\text{\_}\text{asym}\text{\_}І}$. These findings collectively suggest that variations in NOE/asymmetric MT predominantly contribute to ${\text{ΔAREX}}_{\text{asym}}$ at 3.5 ppm, whereas variations in APT and amine CEST appear to have minimal impact.

${\text{AREX}}_{\mathrm{aux}\text{\_}\text{asym}\text{\_}І}$ at 3.5 ppm are 5.82 ± 0.7%s^−1^ and 6.55 ± 2.71%s^−1^ for tumors and normal tissues, respectively, with $B_{1\text{\_}L}$ of 2 μT, and are 13.09 ± 1.49%s^−1^ and 14.74 ± 6.09%s^−1^ for tumors and normal tissues, respectively, with $B_{1\text{\_}L}$ of 3 μT. These values demonstrate the contributions from the amine CEST to ${\text{AREX}}_{\text{asym}}$ at 3.5 ppm. Notably, ${\text{AREX}}_{\mathrm{aux}\text{\_}\text{asym}\text{\_}І}$ at 3.5 ppm roughly increase with $B_{1}^{2}$, which aligns with our previous finding regarding the fast exchange CEST effect.^40^, ^44^, ^45^, ^46^ The small ${\text{AREX}}_{\mathrm{aux}\text{\_}\text{asym}\text{\_}\Pi }$ at 3.5 ppm in tumors and negative ${\text{AREX}}_{\mathrm{aux}\text{\_}\text{asym}\text{\_}\Pi }$ at 3.5 ppm in normal tissues with B_1_ of 2 μT indicate that the APT are comparable to the NOE/asymmetric MT effects in tumors and are smaller than NOE/asymmetric MT effects in normal tissues. Conversely, the positive ${\text{AREX}}_{\mathrm{aux}\text{\_}\text{asym}\text{\_}\Pi }$ at 3.5 ppm in tumors and small ${\text{AREX}}_{\mathrm{aux}\text{\_}\text{asym}\text{\_}\Pi }$ at 3.5 ppm in normal tissues with B_1_ of 3 μT demonstrate that the APT are larger than the NOE/asymmetric MT effects in tumors and are comparable to the NOE/asymmetric MT effects in normal tissues.

### 4.4 Multiple‐pool model Lorentzian fit to evaluate the additive contributor (i.e., APT, NOE) to MTR_asym_ at 15.2 T

**FIGURE 7:**
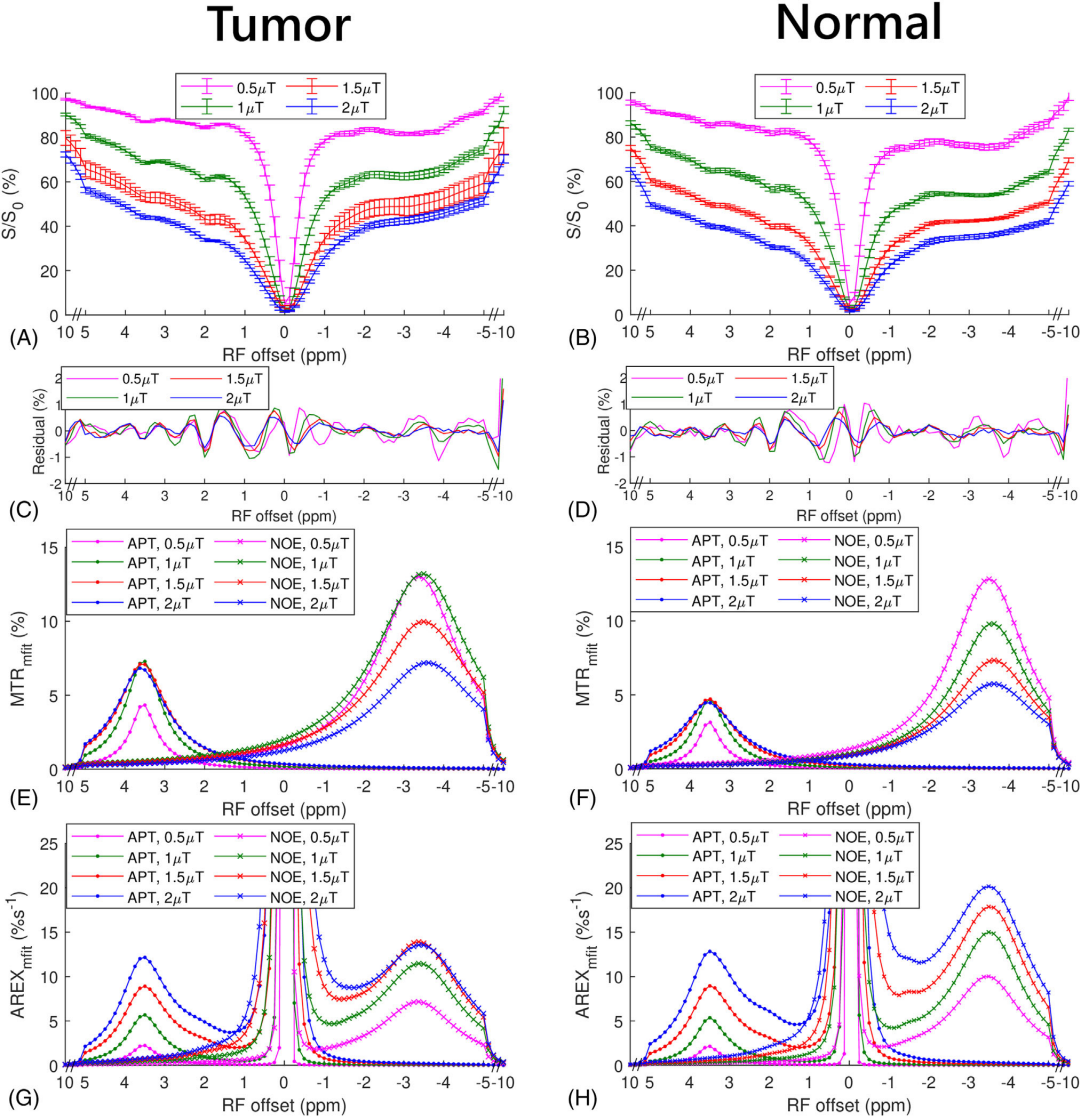
Mean and SD of the CEST Z‐spectra from tumors and contralateral normal tissues in rat brain with B_1_ of 0.5 μT, 1 μT, 1.5 μT, and 2 μT at 15.2 T (A,B), as well as the corresponding mean residual spectra (C,D). The corresponding averaged MTR_mfit_ spectra (E,F) and averaged AREX_mfit_ spectra (G,H) for amide proton transfer (APT) and nuclear Overhauser enhancement (NOE), respectively.

Figure 7A,B presents the Z‐spectra from tumors and normal tissues in the four rats, using B_1_ values of 0.5, 1, 1.5, and 2 μT at 15.2 T. Notably, the APT and NOE peaks are clearly observable on the Z‐spectra with B_1_ of 2 μT. In contrast, these peaks cannot be resolved on the Z‐spectra with the same B_1_ values at 4.7 T. Figure 7C,D show the residual spectra from the mfit method, which are minimal compared to the fitted APT and NOE effects, demonstrating the accuracy of the fitting. Figure 7E–H displays the corresponding ${\mathrm{MTR}}_{\text{mfit}}$ and ${\text{AREX}}_{\text{mfit}}$ spectra.

**FIGURE 8:**
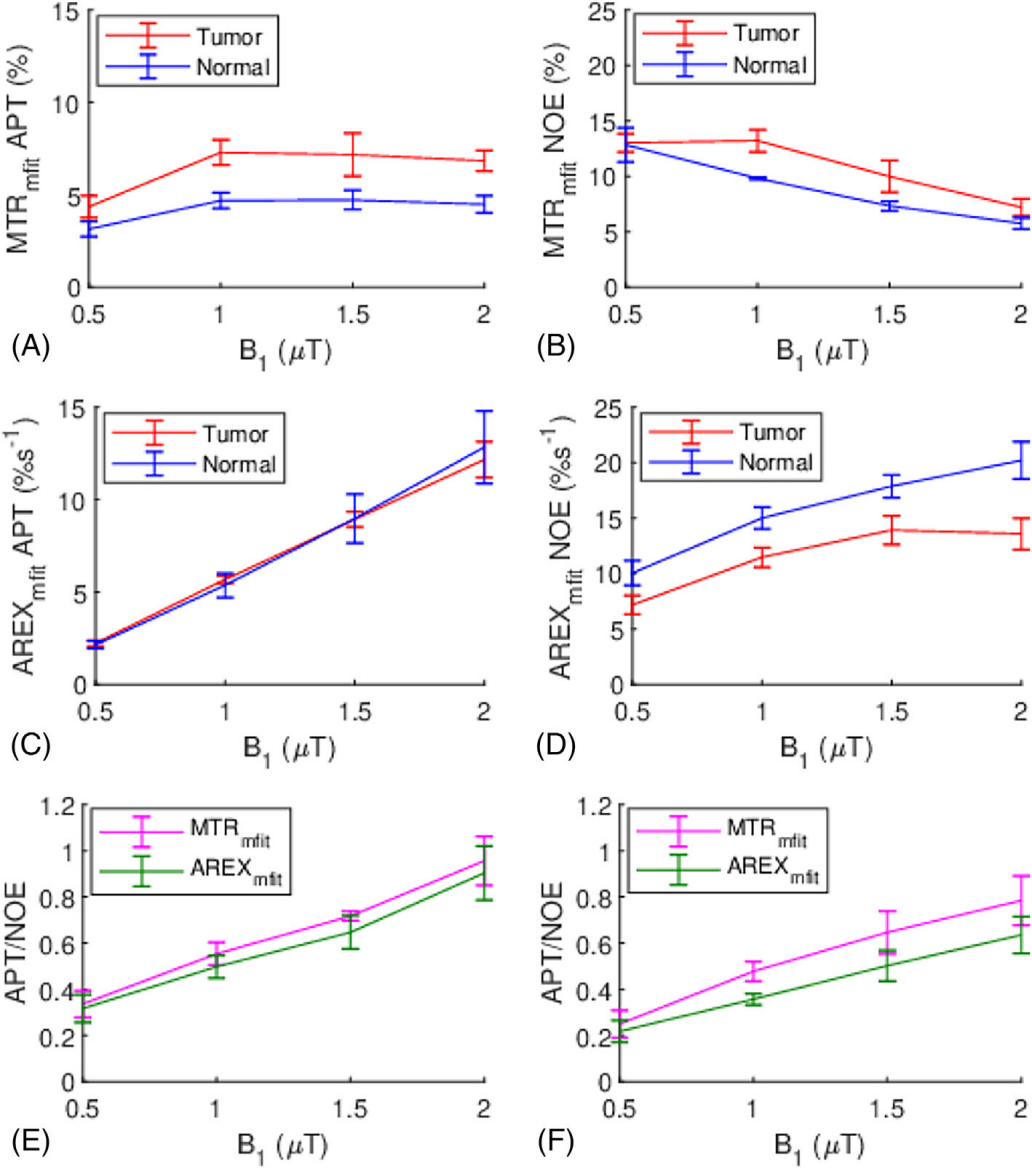
Mean and SD of the MTR_mfit_ and AREX_mfit_‐quantified amide proton transfer (APT) effects (A,C) and the MTR_mfit_ and AREX_mfit_‐quantified and nuclear Overhauser enhancement (NOE) effects (B,D) from tumors and contralateral normal tissues in rats acquired at 15.2 T against B_1_. Mean and SD of ratio of the APT to NOE effects (APT/NOE) quantified by MTR_mfit_ and AREX_mfit_, respectively, from tumors (E) and contralateral normal tissues (F) in rats acquired at 15.2 T against B_1_. The SD is obtained across subjects.

Figure 8A–D presents the ${\mathrm{MTR}}_{\text{mfit}}$ and ${\text{AREX}}_{\text{mfit}}$‐quantified APT and NOE effects in these rats, plotted against B_1_ values. The ${\mathrm{MTR}}_{\text{mfit}}$‐quantified APT effects increase from 0.5 μT and reach plateau from 1 to 2 μT. Additionally, the ${\mathrm{MTR}}_{\text{mfit}}$‐quantified NOE effects decrease from 0.5 to 2 μT. This demonstrates the presence of significant spillover‐dilution from the DS and MT effects, which are more pronounced at higher B_1_ values and reduce the APT and NOE effects. In contrast, using ${\text{AREX}}_{\text{mfit}}$, which removes the spillover‐dilution effect, reveals a gradual increase in both the APT and NOE effects. This demonstrates that the AREX metric is more specific to the CEST effect than the MTR metric. Specifically, the ${\text{AREX}}_{\text{mfit}}$‐quantified APT effects demonstrate a roughly linear increase with B_1_ values, whereas the ${\text{AREX}}_{\text{mfit}}$‐quantified NOE effects reach a plateau at 2 μT, particularly in tumors. This difference is because of their different exchange/coupling rates and suggests the feasibility of increasing the relative size of APT compared to NOE by increasing B_1_ values.

Figure 7E,F presents the ratio of the APT effect to the NOE effect in these rats, quantified by the ${\mathrm{MTR}}_{\text{mfit}}$ and ${\text{AREX}}_{\text{mfit}}$ metrics, plotted against B_1_ values. Notably, these ratios increase with B_1_ but do not reach 1, even at 2 μT. The ratios using ${\mathrm{MTR}}_{\text{mfit}}$ and ${\text{AREX}}_{\text{mfit}}$ metrics at 2 μT are 0.96 ± 0.11 and 0.90 ± 0.12, respectively, in tumors, and are 0.78 ± 0.11 and 0.64 ± 0.08, respectively, in normal tissues. This result is consistent with our study at 4.7 T, confirming that the APT remains comparable to the NOE in tumors and is less than the NOE in normal tissues, even at the high B_1_ of 2 μT. It is also noteworthy that although the dependences of the APT and NOE on B_1_ differ for the ${\mathrm{MTR}}_{\text{mfit}}$ and ${\text{AREX}}_{\text{mfit}}$ metrics, the dependence of their ratios on B_1_ is roughly similar. This is because the spillover‐dilution from the DS and MT effects at ±3.5 ppm are close and therefore, can be canceled by the ratio.

**FIGURE 9:**
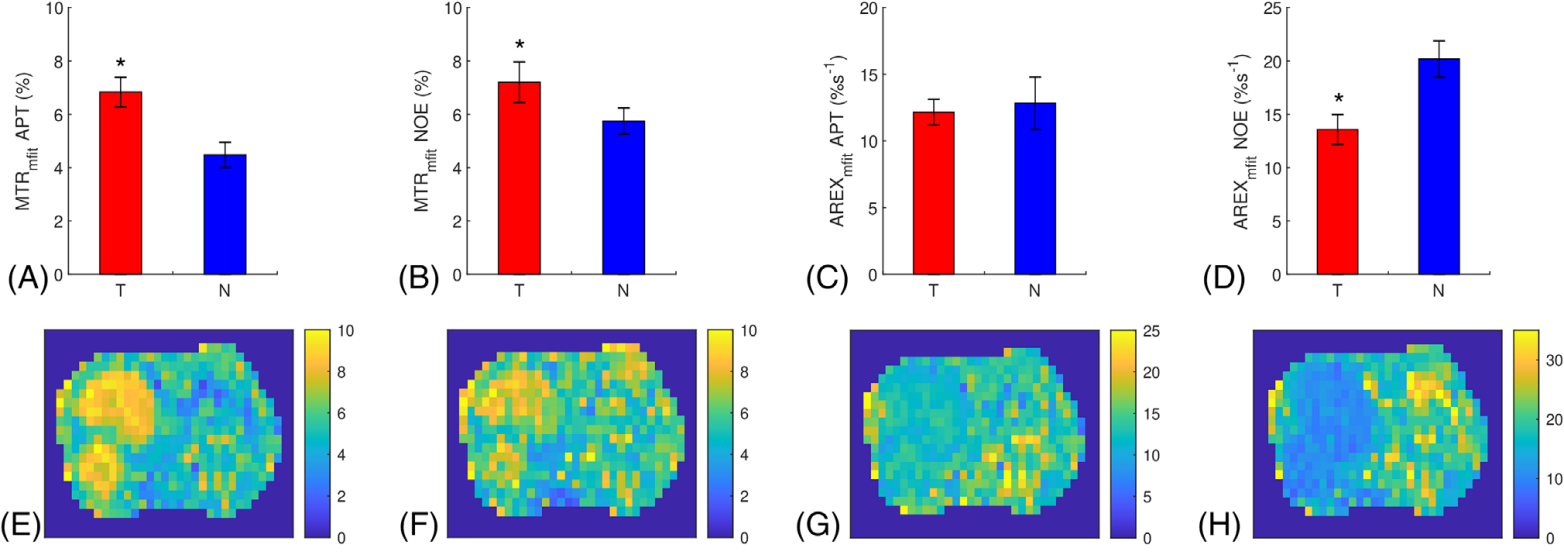
Statistical differences between tumors and contralateral normal tissues for MTR_mfit_‐quantified amide proton transfer (APT) (A), MTR_mfit_‐quantified and nuclear Overhauser enhancement (NOE) (B), AREX_mfit_‐quantified APT (C), AREX_mfit_‐quantified NOE (D), respectively, in rats acquired at 15.2 T. The corresponding maps of the MTR_mfit_‐quantified APT (E), MTR_mfit_‐quantified NOE (F), AREX_mfit_‐quantified APT (G), AREX_mfit_‐quantified NOE (H) from a representative rat acquired at 15.2 T (*p < 0.05).

Figure 9 shows the statistical differences of the ${\mathrm{MTR}}_{\text{mfit}}$‐ and ${\text{AREX}}_{\text{mfit}}$‐quantified APT and NOE effects between tumors and normal tissues with B_1_ of 2 μT, as well as their corresponding maps from a representative rat. Notably, the ${\mathrm{MTR}}_{\text{mfit}}$‐quantified APT effect in tumors is significantly higher than that in normal tissues, whereas the ${\text{AREX}}_{\text{mfit}}$‐quantified APT effect shows no significant difference between tumors and normal tissues. Additionally, the ${\mathrm{MTR}}_{\text{mfit}}$‐quantified NOE effect in tumors is significantly higher than that in normal tissues, whereas the ${\text{AREX}}_{\text{mfit}}$‐quantified NOE effect in tumors is significantly lower than that in normal tissues. This contrasting observation is attributed to the contaminations from the different spillover‐dilution effects in tumors and normal tissues to the ${\mathrm{MTR}}_{\text{mfit}}$ values. The ${\text{AREX}}_{\text{mfit}}$‐quantified APT effects at 3.5 ppm are 12.16 ± 0.96%s^−1^ for tumors and 12.83 ± 1.97%s^−1^ for normal tissues, whereas the ${\text{AREX}}_{\text{mfit}}$‐quantified NOE effects at 3.5 ppm are 13.57 ± 1.41%s^−1^ for tumors and 20.19 ± 1.69%s^−1^ for normal tissues.

### 4.5 Comprehensive analysis of the relative contributions to MTR_asym_ at 4.7 T

At different B_0_, T_2s_ values vary, which is the only factor influencing the dependence of the AREX‐quantified CEST effects on the magnetic fields.^34^ Figure S22 shows that the relative changes in the AREX‐quantified APT and NOE effects with different T_2s_ within our simulation ranges are small (<15%). Therefore, the ${\text{AREX}}_{\text{mfit}}$‐quantified APT and NOE effects with B_1_ of 2 μT at 15.2 T can provide an approximate estimation of the contributions from the APT and NOE effects to ${\text{AREX}}_{\text{asym}}$ at 3.5 ppm with the same B_1_ values at lower B_0_. By evaluating the ratio of each contributor to the sum of the absolute values of the ${\text{AREX}}_{\text{mfit}}$‐quantified APT and NOE effects at 15.2 T and the ${\text{AREX}}_{\mathrm{aux}\text{\_}\text{asym}\text{\_}І}$ values at 3.5 ppm at 4.7 T, we found that the relative contributions for tumors are approximately 39%, 43%, and 18% for the APT, NOE, and amine CEST effects, respectively. For normal tissues, the relative contributions are approximately 32%, 51%, and 17% for the APT, NOE, and amine CEST effects, respectively. When considering the asymmetric MT effect, the relative contribution from APT may be lower, however, this reduction should be minimal because the asymmetric MT is not pronounced at low B_0_. At 3 μT, the APT is higher than the NOE/asymmetric MT effects in tumors and comparable to the NOE/asymmetric MT effects in normal tissues, as demonstrated by the positive ${\text{AREX}}_{\mathrm{aux}\text{\_}\text{asym}\text{\_}\Pi }$ at 3.5 ppm in tumors and the relatively small ${\text{AREX}}_{\mathrm{aux}\text{\_}\text{asym}\text{\_}\Pi }$ at 3.5 ppm in normal tissues in Figure 6. However, the relative contribution from the amine CEST would be higher at 3 μT because its increase with B_1_ is more substantial than the increases for the APT and NOE. This is because of the fact that the amine CEST exhibits a quadratic increase with B_1_, whereas the APT and NOE increase at a lower power. Regarding the APTw contrast between tumors and normal tissues, the lack of significant difference in the ${\text{AREX}}_{\text{mfit}}$‐quantified APT effects between tumors and normal tissues with 2 μT in Figure 9C confirms the finding in Figure 5C that the APT effect has no significant influence on ${\text{ΔAREX}}_{\text{asym}}$.

## 5 DISCUSSION

By using the signal model to investigate the contribution from $T_{1\mathrm{obs}}$ and the spillover‐dilution from the DS and MT effects, using the inverse asymmetry analysis to isolate the amine CEST effect at 4.7 T, and applying the mfit method to separate the APT and NOE effects at 15.2 T, we were able to provide an approximate estimation of relative magnitude of each contributor to the APTw signal and its contrast between tumors and normal tissues at 4.7 T. Our findings indicate that at 2 μT, the APT effect is comparable to the NOE/asymmetric MT effects in tumors. Whereas at 3 μT, the APT effect becomes greater than the NOE/asymmetric MT effects in tumors. These results should also be applicable to 3 T, as the relative magnitudes of the specifically quantified APT and NOE effects should not be significantly influenced by B_0_. Additionally, we found that at these two B_1_ levels, the contribution from the amine CEST cannot be ignored at 4.7 T. At 3 T, the amine CEST effect is expected to be reduced because of the coalescence effect. Nevertheless, the presence of a positive APTw value with B_1_ of 2 μT suggests that the amine contribution may still be non‐negligible.^58^ Furthermore, the APTw contrast between tumors and normal tissues primarily arises from a decreased NOE/asymmetric MT effect in tumors, which is further enhanced by the spillover‐dilution from the reduced MT effect in tumors. Finally, $T_{1\mathrm{obs}}$ does not significantly influence the APTw contrast between tumors and normal tissues.

In our previous study with low B_1_ values at 9.4 T,^41^ the signal model is similar to those in Eqs. ([4) and ([7). However, in this previous model, the three factors, including $\frac{1}{R_{1\mathrm{obs}}}$, $R_{\mathrm{ref}}$, and AREX, were roughly independent because of the minor DS effect. Our findings suggest that the MTR metric depends on $T_{1\mathrm{obs}}$, MT, and clean CEST effect in this previous study. In contrast, in the present study, the DS effect is more pronounced, resulting in a coherence between $\frac{1}{R_{1\mathrm{obs}}}$ and $R_{\mathrm{ref}}$ because of the presence of the $T_{1\mathrm{obs}}$‐related saturation effect in the latter term. The $T_{1\mathrm{obs}}$‐recovery effect and the $T_{1\mathrm{obs}}$‐related saturation effect exert opposing influences on the ${\mathrm{MTR}}_{\text{asym}}$ metric, which effectively cancel each other out, leaving the ${\mathrm{MTR}}_{\text{asym}}$ dependent solely on the MT effect. Consequently, the MTR‐quantified APT contrast with low B_1_ and high B_0_ values has different contributors from the APTw imaging with high B_1_ values. In another study, where we assessed ${\mathrm{MTR}}_{\text{asym}}$ with high B_1_ and low B_0_ values using a two‐pool (solute and water) model analysis,^59^ we also found that ${\mathrm{MTR}}_{\text{asym}}$ was not sensitive to $T_{1\mathrm{obs}}$. However, this previous study is not applicable to tissues because of the absence of multiple major components in the signal model. Therefore, the signal model in the present study is an extension of these two previous studies, aiming to model the APTw signal in tissues with high B_1_ and low B_0_ values.

In this article, we used steady‐state CW‐CEST imaging with a long saturation time, because the AREX metric is only applicable in steady‐state conditions. In clinical APTw imaging, non‐steady‐state CEST imaging is typically used.^7^, ^38^ Figures S23 and S24 show plots of ${\mathrm{MTR}}_{\text{asym}}$ at 3.5 ppm for each pool as a function of the saturation time. We found that the ratios of ${\mathrm{MTR}}_{\text{asym}}$ at 3.5 ppm from the amine, guanidine, and NOE effects to that from the APT effect exhibit minor variations (<5%) when changing from a 5 s saturation to a 2 s saturation. Additionally, these variations are 15.4% and 5.8% for the MT effect with B_1_ of 2 and 3 μT, respectively. These ratios reflect the relative contributions from various pools, which imply that our analysis regarding the contributors to the APTw signals under steady‐state conditions is also applicable under non–steady‐state conditions, without significant bias. Additionally, pulsed‐CEST imaging is commonly used in clinical settings, which may lead to a broader DS effect near the water peak, therefore, potentially resulting in a dependence on T_2w_. We used a six‐pool Lorentzian fit, which accounts for fewer pools compared to the major pools in tissues at high B_0_. For instance, CEST effect at 2.6 ppm, potentially originating from phosphocreatine, has been reported at high field.^60^ Although this effect is significant in muscle, it is notably weak in the brain and is not clearly observable in the Z‐spectra shown in Figure 7. Therefore, excluding this pool is unlikely to influence the fitting accuracy significantly. The amine CEST effect at 3 ppm is prominent in the brain and may originate from both glutamate and proteins, as demonstrated by experiments on phantoms containing glutamate and egg white albumin at physiological concentrations and pH.^61^, ^62^ However, its peak shifts toward the water resonance because of the coalescence effect. To simplify the modeling, we used a single pool to model both the amine and guanidine pools. Figure S25 demonstrates that the fitted amine/guanidine CEST effect is broad and that its amplitude at 3 ppm increases quadratically with B_1_, confirming the successful inclusion of amine. Although omitting certain pools in the six‐pool model may affect fitting accuracy, it tends to overestimate the APT effect. This would suggest that the APTw imaging has less contribution from the APT effect than previously assumed, underscoring the need for careful interpretation of APTw imaging results.

In APTw imaging using the ${\mathrm{MTR}}_{\text{asym}}$ analysis, $T_{1\mathrm{obs}}$ normalization was not suggested, considering that the increase in $T_{1\mathrm{obs}}$ and water content may be canceled out partially.^38^ As a result, the APTw imaging provides a measurement of the solute content, but not its concentration (solute content/water content). In contrast, the AREX metric provides a measure of the solute concentration.^34^ To align with the APTw metric, we can combine ${\text{AREX}}_{\text{asym}}$ and $\frac{1}{R_{1\mathrm{obs}}}$ in Eq. ([4) so that it reflects the solute content. In this scenario, the relative contributions from all multiplicative contributors would remain unchanged because $\frac{1}{R_{1\mathrm{obs}}}$ impacts all CEST effects to the same extent.

In this study, our focus was solely on assessing the contributors to the APTw contrast between tumors and normal tissues. However, in other applications, such as grading tumors, distinguishing active glioma from treatment effects, and so forth, understanding the contributors to the APTw contrast remains a necessity. Our method to separate various contributors can serve as a platform for evaluating these other applications. Additionally, our study used only one tumor model (i.e., 9 L), which has been used previously to validate the relative magnitude of the APT and NOE effects in the APTw imaging.^57^ In other tumor models, further validation is required. Furthermore, this study provides only an approximate estimation of the relative contribution of each effect to the APTw signal and contrast because of the use of simplified models. Despite this limitation, our findings provide a rough overview of the components of the APTw signal and its contrast between tumors and normal tissues, aiding in investigating its molecular mechanisms.

## 6 CONCLUSION

The APTw signal in both tumors and normal tissues is influenced by contributions from APT, NOE/asymmetric MT, and amine CEST effects. The APT effect becomes greater than the NOE/asymmetric MT effects in tumors only at higher B_1_ values (e.g., 3 μT), rather than at the B_1_ of 2 μT. Additionally, the APTw contrast between tumors and normal tissues primarily reflects the decreased NOE/asymmetric MT effects in tumors, with an additional scaling influence from the spillover‐dilution effect of the reduced MT effect in tumors.

## FUNDING INFORMATION

National Institute of Biomedical Imaging and Bioengineering, Grant/Award Numbers: R01 EB029443.

## Supporting information


**Figure S1.** Simulated MTR_asym_ at 3.5 ppm (red) and calculated MTR_asym_ at 3.5 ppm using Equation ([4) (blue) versus APT f_s_ and APT k_sw_ (a, b), T_1w_ and T_2w_ (c, d), f_m_ and k_mw_ (e, f), as well as APT T_2s_ and T_2m_ (g, h) with B_1_ of 2 μT (a, c, e, g) and 3 μT (b, d, f, h), respectively. The normalized root mean square error (NRMSE) between the calculated and the simulated MTR_asym_ values at 3.5 ppm are 7.42%, 2.53%, 5.74%, 2.25%, 5.43%, 2.29%, 4.72%, 2.99%, respectively, in (a–h). The close alignment between the calculated and the simulated MTR_asym_ values at 3.5 ppm validates the approximate model in Equation ([4).
**Figure S2.** Scatter plots between the simulated ${\mathrm{MTR}}_{\text{asym}}/R_{\mathrm{ref}}$ values at 3.5 ppm and 1/R_1obs_ values (a, b), between the simulated ${\mathrm{MTR}}_{\text{asym}}$ values at 3.5 ppm and $R_{\mathrm{ref}}$ values at 3.5 ppm with varied T_2w_ (c, d), between the simulated ${\mathrm{MTR}}_{\text{asym}}$ values at 3.5 ppm and $R_{\mathrm{ref}}$ values at 3.5 ppm with varied f_m_ and k_mw_ (e, f), as well as between the simulated ${\mathrm{MTR}}_{\text{asym}}$ values at 3.5 ppm and ${\text{AREX}}_{\text{asym}}$ values at 3.5 ppm (g, h) with B_1_ of 2 μT (a, c, e, g) and 3 μT (b, d, f, h), respectively. The dashed black lines represent the linear regression of all data points in each subfigure. This simulation suggests that the MTR_asym_ values at 3.5 ppm has a roughly linear dependence on R_1obs_, $R_{\mathrm{ref}}$, and AREX_asym_, respectively. Thus, MTR_asym_ can be approximated as the multiplication of these three terms shown in Equation ([4]). Data in (a, b) were from Figure S1c,d with a series of T_1w_ and a constant T_2w_ of 70 ms. Data in (c, d) were from Figure S1c,d with a series of T_2w_ and a constant T_1w_ of 1.8 s. Data in (e–f) were from Figure S1e,f with a series of f_m_ and k_mw_. Data in (g, h) were from Figure S1a,b with a series of f_s_ and k_sw_.
**Figure S3.** (a, c) show the six‐pool model simulated CEST Z‐spectra with low B_1_ (S_L_) and high B_1_ (S_H_), along with the corresponding auxiliary Z‐spectra (S_A_) with $B_{1\text{\_}L}$ of 2 μT and 3 μT, respectively. (b, d) show the six‐pool model simulated ${\text{AREX}}_{\mathrm{aux}\text{\_}\text{asym}\text{\_}І}$, ${\text{AREX}}_{\text{asym}}$, and ${\text{AREX}}_{\mathrm{aux}\text{\_}\text{asym}\text{\_}\Pi }$ spectra (solid), and the two‐pool (amine and water) and five‐pool (amide, guanidine, NOE, MT, and water) model simulated ${\text{AREX}}_{\text{asym}}$ (dashed), with the corresponding $B_{1\text{\_}L}$. Dotted lines in (b, d) represent the AREX values of 0%s^−1^ and RF offset of 3.5 ppm. The RMSE between the ${\text{AREX}}_{\mathrm{aux}\text{\_}\text{asym}\text{\_}І}$ metric from the six‐pool model simulation and the ${\text{AREX}}_{\text{asym}}$ metric from the two‐pool within the frequency range of 3 and 4 ppm are 0.0020, and 0.0071 for $B_{1\text{\_}L}$ of 2 and 3 μT, respectively. In addition, the RMSE between the ${\text{AREX}}_{\mathrm{aux}\text{\_}\text{asym}\text{\_}\Pi }$ metric from the six‐pool model simulation and the ${\text{AREX}}_{\text{asym}}$ metric from the five‐pool within the frequency range of 3 and 4 ppm are 0.0048 and 0.0127 for $B_{1\text{\_}L}$ of 2 and 3 μT, respectively. These RMSE values are significantly lower than the six‐pool model simulated ${\text{AREX}}_{\text{asym}}$ values at 3.5 ppm, which are 0.0700 and 0.2056 for $B_{1\text{\_}L}$ of 2 μT, and 3 μT, respectively, suggesting close alignment at 3.5 ppm. This result suggests that the ${\text{AREX}}_{\mathrm{aux}\text{\_}\text{asym}\text{\_}І}$ metric can approximately isolate the asymmetry analysis of the amine CEST effect from that of all other effects, whereas the ${\text{AREX}}_{\mathrm{aux}\text{\_}\text{asym}\text{\_}\Pi }$ metric can effectively remove the asymmetry analysis of amine CEST effect from that of all other effects. The ${\text{AREX}}_{\mathrm{aux}\text{\_}\text{asym}\text{\_}І}$ spectra exhibit a gentler slope compared to the ${\text{AREX}}_{\text{asym}}$ spectra, and their values at 5 ppm transition from negative to positive or become higher than ${\text{AREX}}_{\text{asym}}$ values at 5 ppm, attributed to the reduction in the NOE/asymmetric MT effect. Additionally, the small peak at around 3.5 pm in both ${\text{AREX}}_{\text{asym}}$ and ${\text{AREX}}_{\mathrm{aux}\text{\_}\text{asym}\text{\_}\Pi }$ spectra become weaker in the ${\text{AREX}}_{\mathrm{aux}\text{\_}\text{asym}\text{\_}І}$ spectra especially for $B_{1\text{\_}L}$ of 2 μT, due to the capability of ${\text{AREX}}_{\mathrm{aux}\text{\_}\text{asym}\text{\_}І}$ to reduce the APT effect. The differences between the six‐pool model simulated ${\text{AREX}}_{\mathrm{aux}\text{\_}\text{asym}\text{\_}І}$ and the two‐pool model simulated ${\text{AREX}}_{\text{asym}}$, as well as between the six‐pool model simulated ${\text{AREX}}_{\mathrm{aux}\text{\_}\text{asym}\text{\_}\Pi }$ and the five‐pool model simulated ${\text{AREX}}_{\text{asym}}$, at around 2 ppm, are attributed the guanidine CEST effect, which cannot be fully separated using this auxiliary asymmetry analysis method since it is in the intermediate‐exchange regime. However, this difference is minimal at 3.5 ppm because of the relatively narrow peak of the guanidine CEST effect.
**Figure S4.** Six‐pool (amide, amine, guanidine, NOE, MT, and water) model simulated ${\text{AREX}}_{\mathrm{aux}\text{\_}\text{asym}\text{\_}І}$, ${\text{AREX}}_{\text{asym}}$, and ${\text{AREX}}_{\mathrm{aux}\text{\_}\text{asym}\text{\_}\Pi }$ values at 3.5 ppm (solid), and the two‐pool (amine and water) and five‐pool (amide, guanidine, NOE, MT, and water) model simulated ${\text{AREX}}_{\text{asym}}$ values at 3.5 ppm (dashed), as a function of the amide concentration with amide k_sw_ of 40s^−1^ (a), 80s^−1^ (c), and 120 s^−1^ (e), and amide T_2s_ of 2 ms (b), 4 ms (d), and 6 ms (f). $B_{1\text{\_}L}=2\;\mathrm{\mu T}$ and $B_{1\text{\_}H}=6\;\mathrm{\mu T}$. The amide pool concentration was gradually increased to cause different relative size of APT to other effects. Notably, the differences between the six‐pool model simulated ${\text{AREX}}_{\mathrm{aux}\text{\_}\text{asym}\text{\_}І}$ and the two‐pool model simulated ${\text{AREX}}_{\text{asym}}$, as well as between the six‐pool model simulated ${\text{AREX}}_{\mathrm{aux}\text{\_}\text{asym}\text{\_}\Pi }$ and the five‐pool model simulated ${\text{AREX}}_{\text{asym}}$, are much smaller than the six‐pool model simulated ${\text{AREX}}_{\mathrm{aux}\text{\_}\text{asym}\text{\_}І}$, indicating that these two auxiliary asymmetry analysis metrics can effectively separate the amine CEST effect from other effects. Furthermore, the increase in both ${\text{AREX}}_{\text{asym}}$ and ${\text{AREX}}_{\mathrm{aux}\text{\_}\text{asym}\text{\_}\Pi }$ values at 3.5 ppm corresponds with higher amide concentration is due to the increased APT effect. On the other hand, the ${\text{AREX}}_{\mathrm{aux}\text{\_}\text{asym}\text{\_}І}$ values at 3.5 ppm remain relatively stable as they are less affected by the APT effect. The black dashed lines represent the AREX values of 0%s^−1^.
**Figure S5.** Six‐pool (amide, amine, guanidine, NOE, MT, and water) model simulated ${\text{AREX}}_{\mathrm{aux}\text{\_}\text{asym}\text{\_}І}$, ${\text{AREX}}_{\text{asym}}$, and ${\text{AREX}}_{\mathrm{aux}\text{\_}\text{asym}\text{\_}\Pi }$ values at 3.5 ppm (solid), and the two‐pool (amine and water) and five‐pool (amide, guanidine, NOE, MT, and water) model simulated ${\text{AREX}}_{\text{asym}}$ values at 3.5 ppm (dashed), as a function of the amide concentration with amine *k*_sw_ of 3000 s^−1^ (a) and 7000 s^−1^ (c), and amine T_2s_ of 10 ms (b) and 30 ms (d). $B_{1\text{\_}L}=2\;\mathrm{\mu T}$ and $B_{1\text{\_}H}=6\;\mathrm{\mu T}$. The amide pool concentration was gradually increased to cause different relative size of APT to other effects. Notably, the differences between the six‐pool model simulated ${\text{AREX}}_{\mathrm{aux}\text{\_}\text{asym}\text{\_}І}$ and the two‐pool model simulated ${\text{AREX}}_{\text{asym}}$, as well as between the six‐pool model simulated ${\text{AREX}}_{\mathrm{aux}\text{\_}\text{asym}\text{\_}\Pi }$ and the five‐pool model simulated ${\text{AREX}}_{\text{asym}}$, are much smaller than the six‐pool model simulated ${\text{AREX}}_{\mathrm{aux}\text{\_}\text{asym}\text{\_}І}$, indicating that these two auxiliary asymmetry analysis metrics can effectively separate the amine CEST effect from other effects. Furthermore, the increase in both ${\text{AREX}}_{\text{asym}}$ and ${\text{AREX}}_{\mathrm{aux}\text{\_}\text{asym}\text{\_}\Pi }$ values at 3.5 ppm corresponds with higher amide concentration is due to the increased APT effect. On the other hand, the ${\text{AREX}}_{\mathrm{aux}\text{\_}\text{asym}\text{\_}І}$ values at 3.5 ppm remain relatively stable as they are less affected by the APT effect. The black dashed lines represent the AREX values of 0%s^−1^.
**Figure S6.** Six‐pool (amide, amine, guanidine, NOE, MT, and water) model simulated ${\text{AREX}}_{\mathrm{aux}\text{\_}\text{asym}\text{\_}І}$, ${\text{AREX}}_{\text{asym}}$, and ${\text{AREX}}_{\mathrm{aux}\text{\_}\text{asym}\text{\_}\Pi }$ values at 3.5 ppm (solid), and the two‐pool (amine and water) and five‐pool (amide, guanidine, NOE, MT, and water) model simulated ${\text{AREX}}_{\text{asym}}$ values at 3.5 ppm (dashed), as a function of the amide concentration with guanidine k_sw_ of 200 s^−1^ (a) and 800 s^−1^ (c), and guanidine T_2s_ of 10 ms (b) and 30 ms (d). $B_{1\text{\_}L}=2\;\mathrm{\mu T}$ and $B_{1\text{\_}H}=6\;\mathrm{\mu T}$. The amide pool concentration was gradually increased to cause different relative size of APT to other effects. Notably, the differences between the six‐pool model simulated ${\text{AREX}}_{\mathrm{aux}\text{\_}\text{asym}\text{\_}І}$ and the two‐pool model simulated ${\text{AREX}}_{\text{asym}}$, as well as between the six‐pool model simulated ${\text{AREX}}_{\mathrm{aux}\text{\_}\text{asym}\text{\_}\Pi }$ and the five‐pool model simulated ${\text{AREX}}_{\text{asym}}$, are much smaller than the six‐pool model simulated ${\text{AREX}}_{\mathrm{aux}\text{\_}\text{asym}\text{\_}І}$, indicating that these two auxiliary asymmetry analysis metrics can effectively separate the amine CEST effect from other effects. Furthermore, the increase in both ${\text{AREX}}_{\text{asym}}$ and ${\text{AREX}}_{\mathrm{aux}\text{\_}\text{asym}\text{\_}\Pi }$ values at 3.5 ppm corresponds with higher amide concentration is due to the increased APT effect. On the other hand, the ${\text{AREX}}_{\mathrm{aux}\text{\_}\text{asym}\text{\_}І}$ values at 3.5 ppm remain relatively stable as they are less affected by the APT effect. The black dashed lines represent the AREX values of 0%s^−1^.
**Figure S7.** Six‐pool (amide, amine, guanidine, NOE, MT, and water) model simulated ${\text{AREX}}_{\mathrm{aux}\text{\_}\text{asym}\text{\_}І}$, ${\text{AREX}}_{\text{asym}}$, and ${\text{AREX}}_{\mathrm{aux}\text{\_}\text{asym}\text{\_}\Pi }$ values at 3.5 ppm (solid), and the two‐pool (amine and water) and five‐pool (amide, guanidine, NOE, MT, and water) model simulated ${\text{AREX}}_{\text{asym}}$ values at 3.5 ppm (dashed), as a function of the amide concentration with NOE *k*_sw_ of 10s^−1^ (a) and 30s^−1^ (c), and NOE T_2s_ of 0.5 ms (b) and 0.9 ms (d). $B_{1\text{\_}L}=2\;\mathrm{\mu T}$ and $B_{1\text{\_}H}=6\;\mathrm{\mu T}$. The amide pool concentration was gradually increased to cause different relative size of APT to other effects. Notably, the differences between the six‐pool model simulated ${\text{AREX}}_{\mathrm{aux}\text{\_}\text{asym}\text{\_}І}$ and the two‐pool model simulated ${\text{AREX}}_{\text{asym}}$, as well as between the six‐pool model simulated ${\text{AREX}}_{\mathrm{aux}\text{\_}\text{asym}\text{\_}\Pi }$ and the five‐pool model simulated ${\text{AREX}}_{\text{asym}}$, are much smaller than the six‐pool model simulated ${\text{AREX}}_{\mathrm{aux}\text{\_}\text{asym}\text{\_}І}$, indicating that these two auxiliary asymmetry analysis metrics can effectively separate the amine CEST effect from other effects. Furthermore, the increase in both ${\text{AREX}}_{\text{asym}}$ and ${\text{AREX}}_{\mathrm{aux}\text{\_}\text{asym}\text{\_}\Pi }$ values at 3.5 ppm corresponds with higher amide concentration is due to the increased APT effect. On the other hand, the ${\text{AREX}}_{\mathrm{aux}\text{\_}\text{asym}\text{\_}І}$ values at 3.5 ppm remain relatively stable as they are less affected by the APT effect. The black dashed lines represent the AREX values of 0%s^−1^.
**Figure S8.** Six‐pool (amide, amine, guanidine, NOE, MT, and water) model simulated ${\text{AREX}}_{\mathrm{aux}\text{\_}\text{asym}\text{\_}І},{\text{AREX}}_{\text{asym}}$, and ${\text{AREX}}_{\mathrm{aux}\text{\_}\text{asym}\text{\_}\Pi }$ values at 3.5 ppm (solid), and the two‐pool (amine and water) and five‐pool (amide, guanidine, NOE, MT, and water) model simulated ${\text{AREX}}_{\text{asym}}$ values at 3.5 ppm (dashed), as a function of the amide concentration with MT k_sw_ of 15 s^−1^ (a) and 35 s^−1^ (c), and MT T_2s_ of 30 μs (b) and 70 μs (d). $B_{1\text{\_}L}=2\;\mathrm{\mu T}$ and $B_{1\text{\_}H}=6\;\mathrm{\mu T}$. The amide pool concentration was gradually increased to cause different relative size of APT to other effects. Notably, the differences between the six‐pool model simulated ${\text{AREX}}_{\mathrm{aux}\text{\_}\text{asym}\text{\_}І}$ and the two‐pool model simulated ${\text{AREX}}_{\text{asym}}$, as well as between the six‐pool model simulated ${\text{AREX}}_{\mathrm{aux}\text{\_}\text{asym}\text{\_}\Pi }$ and the five‐pool model simulated ${\text{AREX}}_{\text{asym}}$, are much smaller than the six‐pool model simulated ${\text{AREX}}_{\mathrm{aux}\text{\_}\text{asym}\text{\_}І}$, indicating that these two auxiliary asymmetry analysis metrics can effectively separate the amine CEST effect from other effects. Furthermore, the increase in both ${\text{AREX}}_{\text{asym}}$ and ${\text{AREX}}_{\mathrm{aux}\text{\_}\text{asym}\text{\_}\Pi }$ values at 3.5 ppm corresponds with higher amide concentration is due to the increased APT effect. On the other hand, the ${\text{AREX}}_{\mathrm{aux}\text{\_}\text{asym}\text{\_}І}$ values at 3.5 ppm remain relatively stable as they are less affected by the APT effect. The black dashed lines represent the AREX values of 0%s^−1^.
**Figure S9.** Six‐pool (amide, amine, guanidine, NOE, MT, and water) model simulated ${\text{AREX}}_{\mathrm{aux}\text{\_}\text{asym}\text{\_}І}$, ${\text{AREX}}_{\text{asym}}$, and ${\text{AREX}}_{\mathrm{aux}\text{\_}\text{asym}\text{\_}\Pi }$ values at 3.5 ppm (solid), and the two‐pool (amine and water) and five‐pool (amide, guanidine, NOE, MT, and water) model simulated ${\text{AREX}}_{\text{asym}}$ values at 3.5 ppm (dashed), as a function of the amide concentration with amide k_sw_ of 40s^−1^ (a), 80s^−1^ (c), and 120 s^−1^ (e), and amide T_2s_ of 2 ms (b), 4 ms (d), and 6 ms (f). $B_{1\text{\_}L}=3\;\mathrm{\mu T}$ and $B_{1\text{\_}H}=6\;\mathrm{\mu T}$. The amide pool concentration was gradually increased to cause different relative size of APT to other effects. Notably, the differences between the six‐pool model simulated ${\text{AREX}}_{\mathrm{aux}\text{\_}\text{asym}\text{\_}І}$ and the two‐pool model simulated ${\text{AREX}}_{\text{asym}}$, as well as between the six‐pool model simulated ${\text{AREX}}_{\mathrm{aux}\text{\_}\text{asym}\text{\_}\Pi }$ and the five‐pool model simulated ${\text{AREX}}_{\text{asym}}$, are much smaller than the six‐pool model simulated ${\text{AREX}}_{\mathrm{aux}\text{\_}\text{asym}\text{\_}І}$, indicating that these two auxiliary asymmetry analysis metrics can effectively separate the amine CEST effect from other effects. Furthermore, the increase in both ${\text{AREX}}_{\text{asym}}$ and ${\text{AREX}}_{\mathrm{aux}\text{\_}\text{asym}\text{\_}\Pi }$ values at 3.5 ppm corresponds with higher amide concentration is due to the increased APT effect. On the other hand, the ${\text{AREX}}_{\mathrm{aux}\text{\_}\text{asym}\text{\_}І}$ values at 3.5 ppm remain relatively stable as they are less affected by the APT effect. The black dashed lines represent the AREX values of 0%s^−1^.
**Figure S10.** Six‐pool (amide, amine, guanidine, NOE, MT, and water) model simulated ${\text{AREX}}_{\mathrm{aux}\text{\_}\text{asym}\text{\_}І}$, ${\text{AREX}}_{\text{asym}}$, and ${\text{AREX}}_{\mathrm{aux}\text{\_}\text{asym}\text{\_}\Pi }$ values at 3.5 ppm (solid), and the two‐pool (amine and water) and five‐pool (amide, guanidine, NOE, MT, and water) model simulated ${\text{AREX}}_{\text{asym}}$ values at 3.5 ppm (dashed), as a function of the amide concentration with amine k_sw_ of 3000 s^−1^ (a) and 7000 s^−1^ (c), and amine T_2s_ of 10 ms (b) and 30 ms (d). $B_{1\text{\_}L}=3\;\mathrm{\mu T}$ and $B_{1\text{\_}H}=6\;\mathrm{\mu T}$. The amide pool concentration was gradually increased to cause different relative size of APT to other effects. Notably, the differences between the six‐pool model simulated ${\text{AREX}}_{\mathrm{aux}\text{\_}\text{asym}\text{\_}І}$ and the two‐pool model simulated ${\text{AREX}}_{\text{asym}}$, as well as between the six‐pool model simulated ${\text{AREX}}_{\mathrm{aux}\text{\_}\text{asym}\text{\_}\Pi }$ and the five‐pool model simulated ${\text{AREX}}_{\text{asym}}$, are much smaller than the six‐pool model simulated ${\text{AREX}}_{\mathrm{aux}\text{\_}\text{asym}\text{\_}І}$, indicating that these two auxiliary asymmetry analysis metrics can effectively separate the amine CEST effect from other effects. Furthermore, the increase in both ${\text{AREX}}_{\text{asym}}$ and ${\text{AREX}}_{\mathrm{aux}\text{\_}\text{asym}\text{\_}\Pi }$ values at 3.5 ppm corresponds with higher amide concentration is due to the increased APT effect. On the other hand, the ${\text{AREX}}_{\mathrm{aux}\text{\_}\text{asym}\text{\_}І}$ values at 3.5 ppm remain relatively stable as they are less affected by the APT effect. The black dashed lines represent the AREX values of 0%s^−1^.
**Figure S11.** Six‐pool (amide, amine, guanidine, NOE, MT, and water) model simulated ${\text{AREX}}_{\mathrm{aux}\text{\_}\text{asym}\text{\_}І}$, ${\text{AREX}}_{\text{asym}}$, and ${\text{AREX}}_{\mathrm{aux}\text{\_}\text{asym}\text{\_}\Pi }$ values at 3.5 ppm (solid), and the two‐pool (amine and water) and five‐pool (amide, guanidine, NOE, MT, and water) model simulated ${\text{AREX}}_{\text{asym}}$ values at 3.5 ppm (dashed), as a function of the amide concentration with guanidine k_sw_ of 200 s^−1^ (a) and 800 s^−1^ (c), and guanidine T_2s_ of 10 ms (b) and 30 ms (d). $B_{1\text{\_}L}=3\;\mathrm{\mu T}$ and $B_{1\text{\_}H}=6\;\mathrm{\mu T}$. The amide pool concentration was gradually increased to cause different relative size of APT to other effects. Notably, the differences between the six‐pool model simulated ${\text{AREX}}_{\mathrm{aux}\text{\_}\text{asym}\text{\_}І}$ and the two‐pool model simulated ${\text{AREX}}_{\text{asym}}$, as well as between the six‐pool model simulated ${\text{AREX}}_{\mathrm{aux}\text{\_}\text{asym}\text{\_}\Pi }$ and the five‐pool model simulated ${\text{AREX}}_{\text{asym}}$, are much smaller than the six‐pool model simulated ${\text{AREX}}_{\mathrm{aux}\text{\_}\text{asym}\text{\_}І}$, indicating that these two auxiliary asymmetry analysis metrics can effectively separate the amine CEST effect from other effects. Furthermore, the increase in both ${\text{AREX}}_{\text{asym}}$ and ${\text{AREX}}_{\mathrm{aux}\text{\_}\text{asym}\text{\_}\Pi }$ values at 3.5 ppm corresponds with higher amide concentration is due to the increased APT effect. On the other hand, the ${\text{AREX}}_{\mathrm{aux}\text{\_}\text{asym}\text{\_}І}$ values at 3.5 ppm remain relatively stable as they are less affected by the APT effect. The black dashed lines represent the AREX values of 0%s^−1^.
**Figure S12.** Six‐pool (amide, amine, guanidine, NOE, MT, and water) model simulated ${\text{AREX}}_{\mathrm{aux}\text{\_}\text{asym}\text{\_}І}$, ${\text{AREX}}_{\text{asym}}$, and ${\text{AREX}}_{\mathrm{aux}\text{\_}\text{asym}\text{\_}\Pi }$ values at 3.5 ppm (solid), and the two‐pool (amine and water) and five‐pool (amide, guanidine, NOE, MT, and water) model simulated ${\text{AREX}}_{\text{asym}}$ values at 3.5 ppm (dashed), as a function of the amide concentration with NOE k_sw_ of 10s^−1^ (a) and 30s^−1^ (c), and NOE T_2s_ of 0.5 ms (b) and 0.9 ms (d). $B_{1\text{\_}L}=3\;\mathrm{\mu T}$ and $B_{1\text{\_}H}=6\;\mathrm{\mu T}$. The amide pool concentration was gradually increased to cause different relative size of APT to other effects. Notably, the differences between the six‐pool model simulated ${\text{AREX}}_{\mathrm{aux}\text{\_}\text{asym}\text{\_}І}$ and the two‐pool model simulated ${\text{AREX}}_{\text{asym}}$, as well as between the six‐pool model simulated ${\text{AREX}}_{\mathrm{aux}\text{\_}\text{asym}\text{\_}\Pi }$ and the five‐pool model simulated ${\text{AREX}}_{\text{asym}}$, are much smaller than the six‐pool model simulated ${\text{AREX}}_{\mathrm{aux}\text{\_}\text{asym}\text{\_}І}$, indicating that these two auxiliary asymmetry analysis metrics can effectively separate the amine CEST effect from other effects. Furthermore, the increase in both ${\text{AREX}}_{\text{asym}}$ and ${\text{AREX}}_{\mathrm{aux}\text{\_}\text{asym}\text{\_}\Pi }$ values at 3.5 ppm corresponds with higher amide concentration is due to the increased APT effect. On the other hand, the ${\text{AREX}}_{\mathrm{aux}\text{\_}\text{asym}\text{\_}І}$ values at 3.5 ppm remain relatively stable as they are less affected by the APT effect. The black dashed lines represent the AREX values of 0%s^−1^.
**Figure S13.** Six‐pool (amide, amine, guanidine, NOE, MT, and water) model simulated ${\text{AREX}}_{\mathrm{aux}\text{\_}\text{asym}\text{\_}І}$, ${\text{AREX}}_{\text{asym}}$, and ${\text{AREX}}_{\mathrm{aux}\text{\_}\text{asym}\text{\_}\Pi }$ values at 3.5 ppm (solid), and the two‐pool (amine and water) and five‐pool (amide, guanidine, NOE, MT, and water) model simulated ${\text{AREX}}_{\text{asym}}$ values at 3.5 ppm (dashed), as a function of the amide concentration with MT k_sw_ of 15 s^−1^ (a) and 35 s^−1^ (c), and MT T_2s_ of 30 μs (b) and 70 μs (d). $B_{1\text{\_}L}=3\;\mathrm{\mu T}$ and $B_{1\text{\_}H}=6\;\mathrm{\mu T}$. The amide pool concentration was gradually increased to cause different relative size of APT to other effects. Notably, the differences between the six‐pool model simulated ${\text{AREX}}_{\mathrm{aux}\text{\_}\text{asym}\text{\_}І}$ and the two‐pool model simulated ${\text{AREX}}_{\text{asym}}$, as well as between the six‐pool model simulated ${\text{AREX}}_{\mathrm{aux}\text{\_}\text{asym}\text{\_}\Pi }$ and the five‐pool model simulated ${\text{AREX}}_{\text{asym}}$, are much smaller than the six‐pool model simulated ${\text{AREX}}_{\mathrm{aux}\text{\_}\text{asym}\text{\_}І}$, indicating that these two auxiliary asymmetry analysis metrics can effectively separate the amine CEST effect from other effects. Furthermore, the increase in both ${\text{AREX}}_{\text{asym}}$ and ${\text{AREX}}_{\mathrm{aux}\text{\_}\text{asym}\text{\_}\Pi }$ values at 3.5 ppm corresponds with higher amide concentration is due to the increased APT effect. On the other hand, the ${\text{AREX}}_{\mathrm{aux}\text{\_}\text{asym}\text{\_}І}$ values at 3.5 ppm remain relatively stable as they are less affected by the APT effect. The black dashed lines represent the AREX values of 0%s^−1^.
**Figure S14.** Simulated $C_{{\mathrm{MTR}}_{\text{asym}}}$ values at 3.5 ppm (red) and calculated $C_{{\mathrm{MTR}}_{\text{asym}}}$ values at 3.5 ppm using Equation ([7) (blue) versus APT f_s_ and APT k_sw_ (a, b), T_1w_ and T_2w_ (c, d), f_m_ and k_mw_ (e, f), as well as APT T_2s_ and T_2m_ (g, h) with B_1_ of 2 μT (a, c, e, g) and 3 μT (b, d, f, h), respectively. The NRMSE between the calculated and the simulated $C_{{\mathrm{MTR}}_{\text{asym}}}$ values at 3.5 ppm are 2.98%, 2.32%, 0.66%, 0.29%, 0.71%, 1.79%, 0.97%, 1.00%, respectively, in (a–h). The close alignment between the calculated and the simulated $C_{{\mathrm{MTR}}_{\text{asym}}}$ values at 3.5 ppm validates the approximate model in Equation ([7]).
**Figure S15.** Scatter plots between the simulated $C_{{\mathrm{MTR}}_{\text{asym}}}/C_{\mathrm{ref}}$ at 3.5 ppm and $C_{R_{1\mathrm{obs}}}$ (a, b), between the simulated $C_{{\mathrm{MTR}}_{\text{asym}}}$ at 3.5 ppm and $C_{\mathrm{ref}}$ at 3.5 ppm with varied T_2w_ (c, d), between the simulated $C_{{\mathrm{MTR}}_{\text{asym}}}$ at 3.5 ppm and $C_{\mathrm{ref}}$ at 3.5 ppm with varied f_m_ and k_mw_ (e, f), as well as between the simulated $C_{{\mathrm{MTR}}_{\text{asym}}}$ at 3.5 ppm and $C_{{\text{AREX}}_{\text{asym}}}$ at 3.5 ppm (g, h) with B_1_ of 2 μT (a, c, e, g) and 3 μT (b, d, f, h), respectively. The dashed black lines represent the linear regression of all data points in each subfigure. This simulation suggests that the $C_{{\mathrm{MTR}}_{\text{asym}}}$ at 3.5 ppm has a roughly linear dependence on $C_{R_{1\mathrm{obs}}}$, $C_{\mathrm{ref}}$, and $C_{{\text{AREX}}_{\text{asym}}}$, respectively. Thus, $C_{{\mathrm{MTR}}_{\text{asym}}}$ can be approximated as the multiplication of these three terms shown in Equation ([7). Data in (a, b) were from Figure S14c,d with a series of T_1w_ and a constant T_2w_ of 70 ms. Data in (c, d) were from Figure S14c,d with a series of T_2w_ and a constant T_1w_ of 1.8 s. Data in (e–f) were from Figure S14e,f with a series of *f*_m_ and *k*_mw_. Data in (g–h) were from Figure S14a,b with a series of *f*_s_ and *k*_sw_.
**Figure S16.** T_1obs_ maps from five rats acquired at 4.7 T (a–e) and from four rats acquired at 15.2 T (f–i). The T_1obs_ values are 1.9, 1.8, 1.9, 1.9, and 1.7 s in tumors, and 1.5, 1.5, 1.4, 1.4, and 1.4 s in normal tissues of the five rats acquired at 4.7 T. The T_1obs_ values are 2.3, 2.7, 2.4, and 2.3 s in tumors, and 1.9, 2.1, 1.9, and 1.9s in normal tissues of the four rats acquired at 15.2 T. The rats depicted in (a, f), as well as in (b, g), are the same individuals, with images acquired at different scanners within a 24‐h period. For each scanner, four Z‐spectra with varying saturation field strengths and a corresponding T_1_ map were acquired, with the entire imaging session lasting approximately 1 h. Due to the high mortality rate associated with prolonged scanning sessions, other rats scanned at 4.7 and 15.2 T are different individuals.
**Figure S17.** Raw and smoothed CEST Z‐spectra from the tumors (T) and contralateral normal tissues (N) in each rat brain as well as the mean of all brains with B_1_ of 2 μT (a–f), 3 μT (g–l), and 6 μT (m–r), respectively, at 4.7 T.
**Figure S18.** MTR_asym_ (a, c) and AREX_asym_ (b, d) spectra from six‐pool model simulations, along with the simulations without amine/guanidinium CEST, without NOE/asymmetric MT, and without APT, with B_1_ of 2 μT (a, b) and 3 μT (c, d). Notably, the APT effect at 3.5 ppm overlays a sloping baseline on the six‐pool model simulated MTR_asym_ and AREX_asym_ spectra. This sloping baseline decreases in simulations excluding the amine/guanidinium CEST, suggesting its origination from the amine/guanidinium CEST effects. Moreover, the negative values on the six‐pool model simulated MTR_asym_ and AREX_asym_ spectra turn positive in the simulations without the NOE/asymmetric MT effects, indicating that these negative values are attributed to the NOE/asymmetric MT effects.
**Figure S19.** MTR_asym_ (a, e, i, m), AREX_asym_ (b, f, j, n), ΔMTR_asym_ (c, g, k, o), and ΔAREX_asym_ (d, h, l, p) spectra from three‐pool (amide, water, and MT) model simulations with varied APT f_s_ (a–d), T_1w_ (e–h), T_2w_ (i–l), or f_m_ (m–p), while other parameters remained constant. MT pool has no frequency offset. B_1_ is 2 μT.
**Figure S20.** MTR_asym_ (a, e, I, m), AREX_asym_ (b, f, j, n), ΔMTR_asym_ (c, g, k, o), and ΔAREX_asym_ (d, h, l, p) spectra from three‐pool (amide, water, and MT) model simulations with varied APT f_s_ (a–d), T_1w_ (e–h), T_2w_ (i–l), or f_m_ (m–p), while other parameters remained constant. MT pool has no frequency offset. B_1_ is 3 μT.
**Figure S21.** MTR_asym_ (a, e), AREX_asym_ (b, f), ΔMTR_asym_ (c, g), and ΔAREX_asym_ (d, h) spectra from four‐pool (amide, water, NOE(−3.5), and MT) model simulations with variation in both NOE(−3.5) *f*_s_ and MT *f*_m_, while other parameters remained constant. Asymmetric MT was set in this simulation. B_1_ are 2 and 3 μT.
**Figure S22.** Relative change of the APT (a, c) and NOE (b, d) effects with variation in the solute T_2s_ values for a few solute k_sw_ values with B_1_ of 2 μT (a, b) and 3 μT (c, d). The APT and NOE effects were calculated using the formula $f_{s}k_{\mathrm{sw}}\omega_{1}^{2}/\left(\omega_{1}^{2}+k_{\mathrm{sw}}\left(R_{2s}+k_{\mathrm{sw}}\right)\right)$ which represents the AREX quantified CEST effects.^34^ The relative changes of the APT effect were calculated by subtracting the APT effect with an APT T_2_ of 4 ms from those with other APT T_2_ values and normalizing by the APT effect with the APT T_2_ of 4 ms. Similarly, the relative changes of the NOE effect were calculated by subtracting the NOE effect with a NOE T_2_ of 0.7 ms from those with other NOE T_2_ values and normalizing by the NOE effect with the NOE T_2_ of 0.7 ms.
**Figure S23.** MTR_asym_ values at 3.5 ppm against saturation time, with B_1_ of 2 μT from a variety of two‐pool model simulations including amide and water (a), amine and water (b), guanidine and water (c), NOE and water (d), and MT and water (e), respectively. The simulations were conducted for a series of sample parameters for each pool. Dashed line indicates the saturation time of 2 s. The ratios of MTR_asym_ values at 3.5 ppm from each pool to that from APT (ratio_*MTR*_*asym*_), representing their relative contributions, for all combination of sample parameters were first calculated. The variation of this ratio with the saturation time of 2 s (ratio_*MTR*_*asym*__2s) and 5 s (ratio_*MTR*_*asym*__5s) was then calculated by the absolute value of (ratio_*MTR*_*asym*__2s − ratio_*MTR*_*asym*__5s)/ratio_*MTR*_*asym*__5s. The maximum variation of these ratios for all combination of sample parameters are 5.0% for amine, 3.0% for guanidine, 3.9% for NOE, and 15.4% for MT, respectively.
**Figure S24.** *MTR*_*asym*_ values at 3.5 ppm with B_1_ of 3 μT from a variety of two‐pool model simulations including amide and water (a), amine and water (b), guanidine and water (c), NOE (−3.5 ppm) and water (d), and MT and water (e), respectively. The simulations were conducted for a series of sample parameters for each pool. Dashed line indicates the saturation time of 2 s. The ratios of *MTR*_*asym*_ values at 3.5 ppm from each pool to that from APT (ratio_*MTR*_*asym*_), representing their relative contributions, for all combination of sample parameters were first calculated. The variation of this ratio with the saturation time of 2 s (ratio_*MTR*_*asym*__2s) and 5 s (ratio_*MTR*_*asym*__5s) was then calculated by the absolute value of (ratio_*MTR*_*asym*__2s − ratio_*MTR*_*asym*__5s) / ratio_*MTR*_*asym*__5s. The maximum variation of these ratios for all combination of sample parameters are 3.9% for amine, 1.4% for guanidine, 2.1% for NOE, and 5.8% for MT, respectively.
**Figure S25.** Averaged *AREX*_*mfit*_ spectra for APT, amine/guanidine CEST, and NOE effects from tumors (a) and contralateral normal tissues (b) in rat brain with B_1_ of 0.5, 1, 1.5, and 2 μT at 15.2 T. Mean and standard deviation of the *AREX*_*mfit*_‐quantified amine/guanidine CEST effects at 3 ppm, from tumor and normal tissues, as a function of B_1_ values. Guan is the abbreviation of guanidine.
**Table S1.** Starting points and boundaries of the extrapolated MT reference (EMR) fitting parameters.
**Table S2.** Extrapolated MT reference (EMR) fitted MT parameters from the tumors and the contralateral normal tissues in the five rats measured at 4.7 T.
**Table S3.** Starting points and boundaries of the amplitude, width, and offset of the exchange/coupling pools in the Lorentzian fit. The unit of peak width and offset is ppm. The NOE effect at −1.6 ppm was termed NOE (−1.6), while the NOE effect at −3.5 ppm was termed NOE (−3.5).
