# Supplementary material for: Interpreting amide proton transfer‐weighted imaging contrast between normal and tumor brain tissues using the asymmetry analysis method at 4.7 T
Source: Magn Reson Med. 2025 Aug 24;95(1):485–505. doi: 10.1002/mrm.70041 (PMC12620183; doi:10.1002/mrm.70041)
Supplement: Supplementary file 1 — Figure S1. Simulated MTRasym at 3.5 ppm (red) and calculated MTRasym at 3.5 ppm using Equation ([4) (blue) versus APT fs and APT ksw (a, b), T1w and T2w (c, d), fm and kmw (e, f), as well as APT T2s and T2m (g, h) with B1 of 2 μT (a, c, e, g) and 3 μT (b, d, f, h), respectively. The normalized root mean square error (NRMSE) between the calculated and the simulated MTRasym values at 3.5 ppm are 7.42%, 2.53%, 5.74%, 2.25%, 5.43%, 2.29%, 4.72%, 2.99%, respectively, in (a–h). The close alignment between the calculated and the simulated MTRasym values at 3.5 ppm validates the approximate model in Equation ([4). Figure S2. Scatter plots between the simulated MTRasym/Rref values at 3.5 ppm and 1/R1obs values (a, b), between the simulated MTRasym values at 3.5 ppm and Rref values at 3.5 ppm with varied T2w (c, d), between the simulated MTRasym values at 3.5 ppm and Rref values at 3.5 ppm with varied fm and kmw (e, f), as well as between the simulated MTRasym values at 3.5 ppm and AREXasym values at 3.5 ppm (g, h) with B1 of 2 μT (a, c, e, g) and 3 μT (b, d, f, h), respectively. The dashed black lines represent the linear regression of all data points in each subfigure. This simulation suggests that the MTRasym values at 3.5 ppm has a roughly linear dependence on R1obs, Rref, and AREXasym, respectively. Thus, MTRasym can be approximated as the multiplication of these three terms shown in Equation ([4]). Data in (a, b) were from Figure S1c,d with a series of T1w and a constant T2w of 70 ms. Data in (c, d) were from Figure S1c,d with a series of T2w and a constant T1w of 1.8 s. Data in (e–f) were from Figure S1e,f with a series of fm and kmw. Data in (g, h) were from Figure S1a,b with a series of fs and ksw. Figure S3. (a, c) show the six‐pool model simulated CEST Z‐spectra with low B1 (SL) and high B1 (SH), along with the corresponding auxiliary Z‐spectra (SA) with B1_L of 2 μT and 3 μT, respectively. (b, d) show the six‐pool model simulated AREXaux_asym_І, AREXasym, and AREX [file MRM-95-485-s001.docx]

**Supporting information**

**Supporting information Method**

Numerical simulation of coupled Bloch equations were performed utilizing the ordinary differential equation solver (ODE45) in MATLAB (Math works, Natick, MA, USA) to evaluate the accuracy of the proposed approximate models in Eq. (4) and Eq. (7), as well as the capability of the auxiliary asymmetry analysis metric to separate the amine CEST effect from other effects. The simulation model contains six pools including amide at 3.5ppm, amine at 3ppm, guanidine at 2ppm, water at 0mm, NOE at -3.5ppm, and MT at -2.3ppm(1), representing major tissue components. Sample parameters were from the following table, except where it was noted. The MT pool was modeled using a Lorentzian line shape.

|  | water | amide | amine | Guanidine | NOE(-3.5ppm) | MT |
| --- | --- | --- | --- | --- | --- | --- |
| f_s_ (%) | 100 | 0.1 | 0.3 | 0.1 | 0.5 | 5 |
| k_sw_ (s^-1^) | - | 80^a^ | 5000^b^ | 500^c^ | 20 | 25 |
| T_1_ (s) | 1.5 | 1.5 | 1.5 | 1.5 | 1.5 | 1.5 |
| T_2_ (ms) | 60 | 4 | 20 | 20 | 0.7 | 0.05 |
| Δ (ppm) | 0 | 3.5 | 3 | 2 | -3.5 | -2.3 |

^a^ _ 30s^-1^ to 300s^-1^ in Ref (2-7),

^b^ _ 5000s^-1^ in Ref (8,9)

^c^ _ 240s^-1^ to 1000s^-1^ in Ref (10-12)

**1.** To evaluate the accuracy of the approximate model in Eq. (4) in Supporting information Figure S1, we compared the calculated MTR_asym_ values at 3.5ppm using Eq. (4) with the simulated MTR_asym_ values at 3.5ppm for a series of varied sample parameters including including f_s_ and k_sw_, T_1w_ and T_2w_, f_m_ and MT coupling rate (k_mw_), or T_2s_ and MT pool transverse relaxation time (T_2m_). To calculate MTR_asym_ values at 3.5ppm using Eq. (4), R_1obs_ was obtained using (R_1w_+f_m_R_1m_)/(1+f_m_), in which R_1m_ is the longitudinal relaxation rate of the MT pool(13); S_ref_ was obtained from the simulations by setting the corresponding APT pool concentration to 0; AREX_asym_ at 3.5ppm was obtained by the inverse asymmetry analysis of the simulated CEST signals ±3.5ppm.

To further evaluate the separate dependence of the MTR_asym_ metric on each contributor (shown in Eq. (4)) in Supporting information Figure S2, we performed scatter plots between ${MTR}_{asym}/R_{ref}$ at 3.5ppm and $\frac{1}{R_{1obs}}$, between MTR_asym_ at 3.5ppm and $R_{ref}$ at 3.5ppm, as well as between MTR_asym_ at 3.5ppm and AREX_asym_ at 3.5ppm. Since R_1obs_ also influences S_ref_, which we called the R_1obs_-related saturation effect(14), we cannot provide a completely separate evaluation of the dependence of the MTR_asym_ values at 3.5ppm on $\frac{1}{R_{1obs}}$ through a scatter plot between them. Thus, we first normalized MTR_asym_ values at 3.5ppm by $R_{ref}$ at 3.5ppm to remove its contribution, and then performed a scatter plot between the normalized MTR_asym_ values at 3.5ppm and $\frac{1}{R_{1obs}}$ to evaluate their relationship. Specifically, to evaluate the separate dependence of MTR_asym_ on $\frac{1}{R_{1obs}}$, ${MTR}_{asym}/R_{ref}$ values were simulated with varied T_1w_ but other parameters kept constant which should change both the $\frac{1}{R_{1obs}}$ effect and $R_{ref}$ effect but not the AREX_asym_ effect. To evaluate the separate dependence of MTR_asym_ on the $R_{ref}$ effect, MTR_asym_ values were simulated with either varied T_2w_, causing varied DS effect, or varied f_m_ and k_mw_, causing varied MT effect, but other parameters kept constant which should change the $R_{ref}$ effect but have weak or no influence on the other two contributors. It is worth noting that to avoid the influence of the asymmetric MT effect on AREX_asym_ in evaluating the dependence of MTR_asym_ on MT-related $R_{ref}$ effect, the frequency offset of the MT pool was set to 0 for simulations with varied f_m_ and k_mw_. To evaluate the separate dependence of MTR_asym_ on the AREX_asym_ effect, MTR_asym_ values were simulated with varied f_s_ and k_sw_ but other parameters kept constant which should change the AREX_asym_ effect but have no influence on the other two contributors.

**2.** To evaluate the accuracy of the approximate model in Eq. (7) in Supporting information Figure S14, we compared the calculated $C_{{MTR}_{asym}}$ values at 3.5ppm using this equation with the simulated $C_{{MTR}_{asym}}$ values at 3.5ppm for a series of varied sample parameters. $C_{{MTR}_{asym}}$ values were obtained using ${MTR}_{asym\_t}$/${MTR}_{asym\_n}$ in which ${MTR}_{asym\_t}$ values were obtained with a series of varied sample parameters, and ${MTR}_{asym\_n}$ values were obtained with a constant set of sample parameters.

To further evaluate the separate dependence of $C_{{MTR}_{asym}}$ on each contributors including $C_{R_{1obs}}$, $C_{ref}$, and $C_{{AREX}_{asym}}$ in Eq. (7) in Supporting information Figure S15, we performed scatter plots between $C_{{MTR}_{asym}} /C_{ref}$ values at 3.5ppm and $C_{R_{1obs}}$ values, between $C_{{MTR}_{asym}}$ values at 3.5ppm and $C_{ref}$ values at 3.5ppm with either varied T_2w_ or varied f_m_ and k_mw_ (the frequency offset of the MT pool was set to 0 for simulations with varied f_m_ and k_mw_), as well as between $C_{{MTR}_{asym}}$ values at 3.5ppm and $C_{{AREX}_{asym}}$ values at 3.5ppm, similar to that for evaluating MTR_asym_.

**3.** To evaluate the effectiveness of the ${AREX}_{aux\_asym\_І}$ metric in reflecting the asymmetry analysis of the amine CEST effects, and the ${AREX}_{aux\_asym\_\Pi}$ metric in removing the asymmetry analysis of the amine CEST effect, we compared these two asymmetric auxiliary metrics derived from the six-pool model simulations with ${AREX}_{asym}$ values derived from simulations that either included only the amine CEST effect or excluded it. Specifically, CEST Z-spectra were simulated using $B_{1\_L}$ values of 2µT, and 3 µT, along with $B_{1\_H}$ value of 6 µT. Initially, simulations were conducted using the six-pool model to obtain ${AREX}_{aux\_asym\_І}$, ${AREX}_{asym}$, and ${AREX}_{aux\_asym\_\Pi}$ values. Subsequently, simulations were performed using a two-pool model (amine and water) to solely display the contributions from the amine CEST effect. Additionally, a five-pool model (amide, guanidine, NOE at -3.5ppm, MT, and water) simulation was used to solely show the contributions from all other effects except for the amine. The ${AREX}_{asym}$ values were then calculated from these two-pool and five-pool model simulated data. The ${AREX}_{aux\_asym\_І}$ values from the six-pool simulation were compared with the ${AREX}_{asym}$ values from the two-pool model simulations to determine whether the ${AREX}_{aux\_asym\_І}$ metric can effectively assess the contributions from the asymmetry analysis of the amine CEST effects. Similarly, the ${AREX}_{aux\_asym\_\Pi}$ values from the six-pool model simulations were compared with the ${AREX}_{asym}$ values from the five-pool simulations to evaluate whether the ${AREX}_{aux\_asym\_\Pi}$ metric can assess the contributions from all other effects. In Supporting information Figure S3, we compared these AREX spectra obtained using the simulation parameters listed in Supporting Information Table S4. In Supporting information Figures S4-S13, we compared their AREX values at 3.5ppm for a range of sample parameter values.

**4.** To evaluate the separate contribution from the amine/guanidinium CEST effects, NOE/asymmetric MT effects, and APT effect to the MTR_asym_ and AREX_asym_ spectra in Supporting Information Figure S18, a six-pool model simulation with/without the corresponding pools were conducted.

**5.** To show how the APT f_s_, T_1w_, T_2w_, and f_m_ influence the APT peak in the MTR_asym_, AREX_asym_, ΔMTR_asym_, ΔAREX_asym_ spectra in Supporting information Figures S19 and S20, a three-pool (APT, water, and MT) model simulation was performed with variations in these parameters while keeping other parameters constant. Notably, the MT frequency offset was set to 0 to avoid the influence from the asymmetric MT effect. To show how the NOE/asymmetric MT effects influence the APT peak in the MTR_asym_, AREX_asym_, ΔMTR_asym_, ΔAREX_asym_ spectra in Supporting information Figure S21, a four-pool (APT, water, NOE, and MT) model simulation was performed with variations in both the NOE f_s_ and MT f_m_ while keeping other parameters constant. Asymmetric MT was set in this simulation.

**6.** To evaluate the dependence of the MTR_asym_ values at 3.5ppm from each pool on the saturation time in Supporting Information Figures S23 and S24, we performed a variety of two-pool model simulations including amide + water, amine + water, guanidine + water, NOE(-3.5) + water, and MT + water.

**Supporting information Discussion**

For a two-pool (solute and water) model, Eq. (4) can be expressed as,

${MTR}_{asym}\text{(Δω)=}\frac{1}{\underset{T_{1w}\mathrm{recovery}}{\underbrace{R_{1w}}}}\cdot\underset{T_{1w}-related saturation}{\underbrace{{(\frac{\text{ω}_{1}^{2}+\text{Δω}^{2}}{\frac{R_{2w}}{R_{1w}}\text{ω}_{1}^{2}+\text{Δω}^{2}})}^{2}}}\cdot{AREX}_{asym}\text{(Δω)}$ (S1)

When there is no DS effect ($\omega_{1}\ll\text{Δω}$), ${AREX}_{asym}$ is scaled only by $R_{1w}$ in the first item in Eq. (S1), which we refer to as $T_{1w}$ recovery effect. However, in the presence of the DS effect, ${AREX}_{asym}$ is scaled by $R_{1w}$ in both the first and second items in Eq. (S1). Since the second item is related the DS effect, we refer to $R_{1w}$ in the second item as the T_1w_-related saturation effect. Notably, these two $R_{1w}$-related effects exert opposing influences on ${MTR}_{asym}$. Thus, with appropriate $\omega_{1}$ and $\text{Δω}$, these opposing $R_{1w}$-related effects can cancel each other out, resulting in ${MTR}_{asym}$ becoming insensitive to $R_{1w}$.

**Supporting information Table S1.** Starting points and boundaries of the EMR fitting parameters.

|  | Start | Lower | Upper |
| --- | --- | --- | --- |
| k_mw_ (s^-1^) | 25 | 0 | 100 |
| T_2m_ (μs) | 25 | 10 | 100 |
| k_mw_f_m_T_1w_ | 3 | 0 | 50 |
| Δ_m_ (ppm) | 0 | -3 | 3 |

**Supporting information Table S2**: EMR fitted MT parameters from the tumors and the contralateral normal tissues in the five rats measured at 4.7T.

| n=5 | k_mw_ (s^-1^) | T_2m_ (μs) | k_mw_f_m_T_1w_ | Δ_m_ (ppm) |
| --- | --- | --- | --- | --- |
| Tumor | 28.3 ± 3.6 | 64.6 ± 12.0 | 2.1 ± 0.3 | -1.0 ± 0.2 |
| Normal | 27.8± 3.4 | 69.1 ± 11.2 | 3.2 ± 0.4 | -1.3 ± 0.2 |

**Supporting information Table S3.** Starting points and boundaries of the amplitude, width, and offset of the exchange/coupling pools in the Lorentzian fit. The unit of peak width and offset is ppm. The NOE effect at -1.6ppm was termed NOE(-1.6), while the NOE effect at -3.5ppm was termed NOE(-3.5).

|  | Start | Lower | Upper |
| --- | --- | --- | --- |
| A_water_ | 0.9 | 0.02 | 1 |
| W_water_ | 1.4 | 0.1 | 10 |
| Δ_water_ | 0 | -1 | 1 |
| A_amide_ | 0.025 | 0 | 0.2 |
| W_amide_ | 0.5 | 0.4 | 3 |
| Δ_amide_ | 3.5 | 3 | 4 |
| A_amine/guanidine_ | 0.01 | 0 | 0.2 |
| W_amine/guanidine_ | 1.5 | 0.5 | 5 |
| Δ_amine/guanidine_ | 2 | 1 | 3 |
| A_NOE(-1.6)_ | 0.001 | 0 | 0.2 |
| W_NOE(-1.6)_ | 1 | 0 | 1.5 |
| Δ_NOE(-1.6)_ | -1.5 | -2 | -1 |
| A_NOE(-3.5)_ | 0.02 | 0 | 1 |
| W_NOE(-3.5)_ | 3 | 1 | 5 |
| Δ_NOE(-3.5)_ | -3.5 | -4.5 | -2.5 |
| A_MT_ | 0.1 | 0 | 1 |
| W_MT_ | 25 | 10 | 100 |
| Δ_MT_ | 0 | -4 | 4 |

2µT 3µT

**
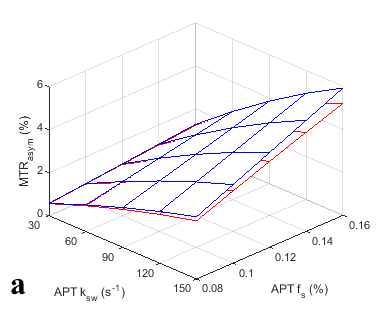

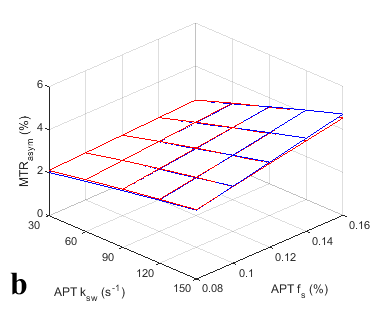

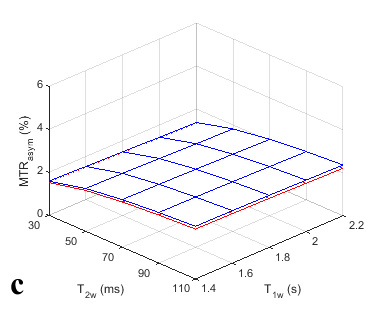

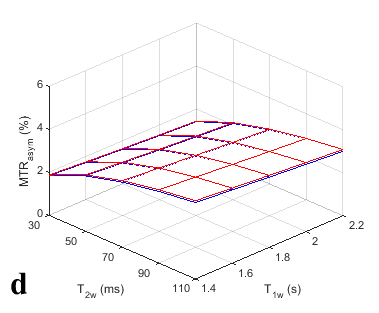

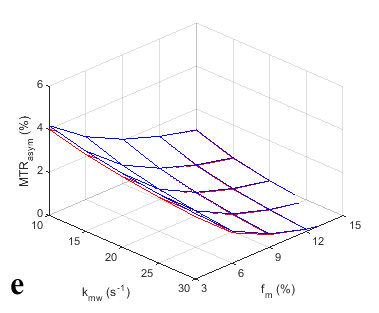

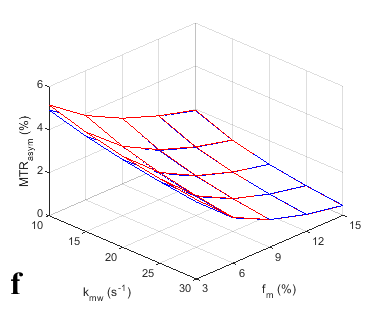

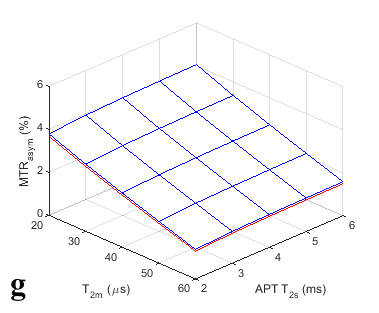

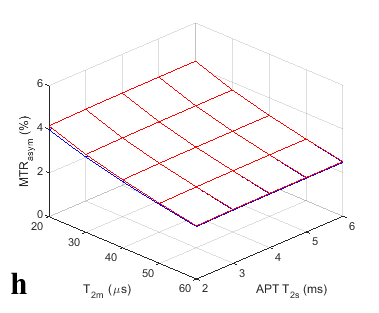
**

**Supporting information Figure S1.** Simulated MTR_asym_ at 3.5ppm (red) and calculated MTR_asym_ at 3.5ppm using Eq. (4) (blue) vs. APT f_s_ and APT k_sw_ (a, b), T_1w_ and T_2w_ (c, d), f_m_ and k_mw_ (e, f), as well as APT T_2s_ and T_2m_ (g, h) with B_1_ of 2µT (a, c, e, and g) and 3µT (b, d, f, and h), respectively. The normalized root mean square error (NRMSE) between the calculated and the simulated MTR_asym_ values at 3.5ppm are 7.42%, 2.53%, 5.74%, 2.25%, 5.43%, 2.29%, 4.72%, 2.99%, respectively, in (a-h). The close alignment between the calculated and calculated and the simulated MTR_asym_ values at 3.5ppm validates the approximate model in Eq. (4).

2µT 3µT

**
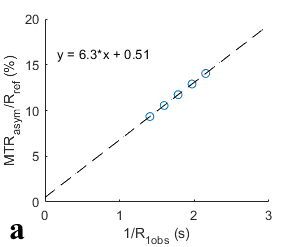

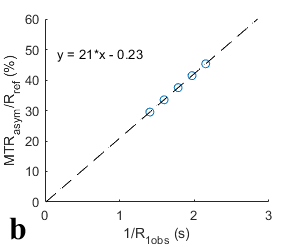

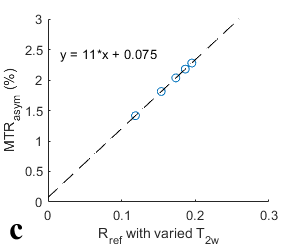

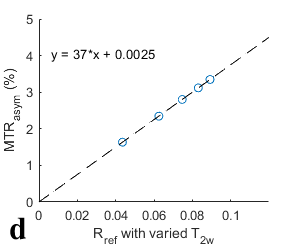

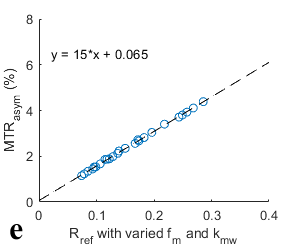

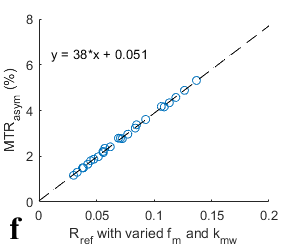

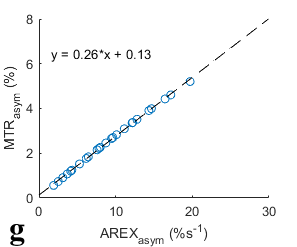

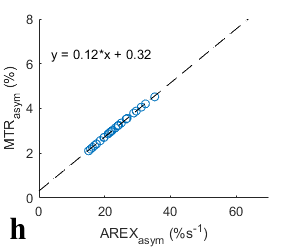
**

**Supporting information Figure S2.** Scatter plots between the simulated ${MTR}_{asym}/R_{ref}$ values at 3.5ppm and 1/R_1obs_ values (a, b), between the simulated ${MTR}_{asym}$ values at 3.5ppm and $R_{ref}$ values at 3.5ppm with varied T_2w_ (c, d), between the simulated ${MTR}_{asym}$ values at 3.5ppm and $R_{ref}$ values at 3.5ppm with varied f_m_ and k_mw_ (e, f), as well as between the simulated ${MTR}_{asym}$ values at 3.5ppm and ${AREX}_{asym}$ values at 3.5ppm (g, h) with B_1_ of 2µT (a, c, e, and g) and 3µT (b, d, f, and h), respectively. The dashed black lines represent the linear regression of all data points in each subfigure. This simulation suggests that the MTR_asym_ values at 3.5ppm has a roughly linear dependence on R_1obs_, $R_{ref}$, and AREX_asym_, respectively. Thus, MTR_asym_ can be approximated as the multiplication of these three terms shown in Eq. (4). Data in (a, b) were from Supporting information Figure S1c and S1d with a series of T_1w_ and a constant T_2w_ of 70ms. Data in (c, d) were from Supporting information Figure S1c and S1d with a series of T_2w_ and a constant T_1w_ of 1.8s. Data in (e-f) were from Supporting information Figure S1e and S1f with a series of f_m_ and k_mw_. Data in (g-h) were from Supporting information Figure S1a and S1b with a series of f_s_ and k_sw_.


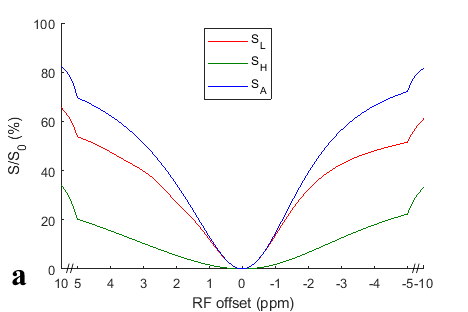

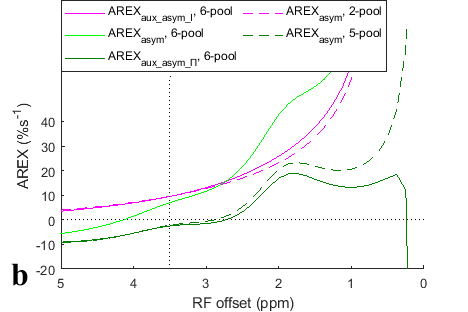


B_1_L_=2µT


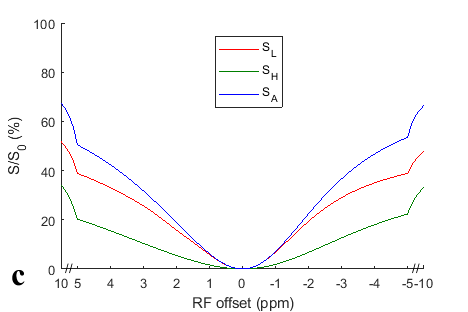

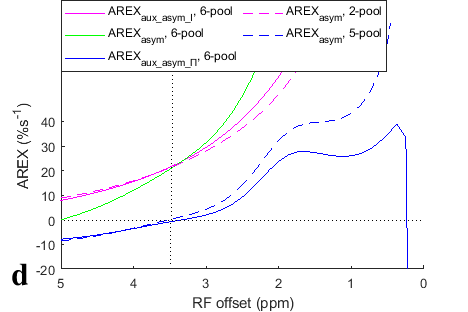


B_1_L_=3µT

**Supporting information Figure S3.** (a, c) show the six-pool model simulated CEST Z-spectra with low B_1_ (S_L_) and high B_1_ (S_H_), along with the corresponding auxiliary Z-spectra (S_A_) with $B_{1\_L}$ of 2µT and 3 µT, respectively. (b, d) show the six-pool model simulated ${AREX}_{aux\_asym\_І}$_,_ ${AREX}_{asym}$, and ${AREX}_{aux\_asym\_\Pi}$ spectra (solid), and the two-pool (amine and water) and five-pool (amide, guanidine, NOE, MT, and water) model simulated ${AREX}_{asym}$ (dashed), with the corresponding $B_{1\_L}$. Dotted lines in (b, d) represent the AREX values of 0%s^-1^ and RF offset of 3.5ppm. The RMSE between the ${AREX}_{aux\_asym\_І}$ metric from the six-pool model simulation and the ${AREX}_{asym}$ metric from the two-pool within the frequency range of 3 ppm and 4 ppm are 0.0020, and 0.0071 for $B_{1\_L}$ of 2µT and 3µT, respectively. In addition, the RMSE between the ${AREX}_{aux\_asym\_\Pi}$ metric from the six-pool model simulation and the ${AREX}_{asym}$ metric from the five-pool within the frequency range of 3ppm and 4ppm are 0.0048 and 0.0127 for $B_{1\_L}$ of 2µT and 3µT, respectively. These RMSE values are significantly lower than the six-pool model simulated ${AREX}_{asym}$ values at 3.5ppm, which are 0.0700 and 0.2056 for $B_{1\_L}$ of 2µT, and 3µT, respectively, suggesting close alignment at 3.5ppm. This result suggests that the ${AREX}_{aux\_asym\_І}$ metric can approximately isolate the asymmetry analysis of the amine CEST effect from that of all other effects, while the ${AREX}_{aux\_asym\_\Pi}$ metric can effectively remove the asymmetry analysis of amine CEST effect from that of all other effects. The ${AREX}_{aux\_asym\_І}$ spectra exhibit a gentler slope compared to the ${AREX}_{asym}$ spectra, and their values at 5ppm transition from negative to positive or become higher than ${AREX}_{asym}$ values at 5ppm, attributed to the reduction in the NOE/asymmetric MT effect. Additionally, the small peak at around 3.5pm in both ${AREX}_{asym}$ and ${AREX}_{aux\_asym\_\Pi}$ spectra become weaker in the ${AREX}_{aux\_asym\_І}$ spectra especially for $B_{1\_L}$ of 2µT, due to the capability of ${AREX}_{aux\_asym\_І}$to reduce the APT effect. The differences between the six-pool model simulated ${AREX}_{aux\_asym\_І}$ and the two-pool model simulated ${AREX}_{asym}$, as well as between the six-pool model simulated ${AREX}_{aux\_asym\_\Pi}$ and the five-pool model simulated ${AREX}_{asym}$, at around 2ppm, are attributed the guanidine CEST effect, which cannot be fully separated using this auxiliary asymmetry analysis method since it is in the intermediate-exchange regime. However, this difference is minimal at 3.5ppm because of the relatively narrow peak of the guanidine CEST effect.

**
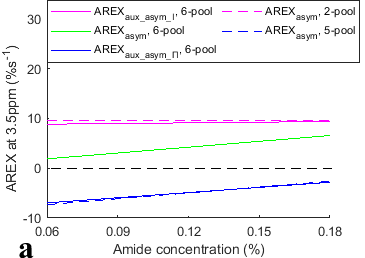

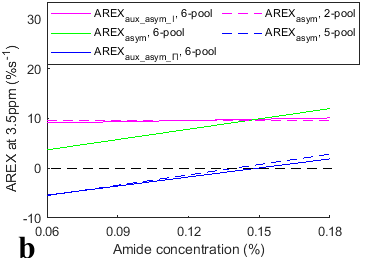

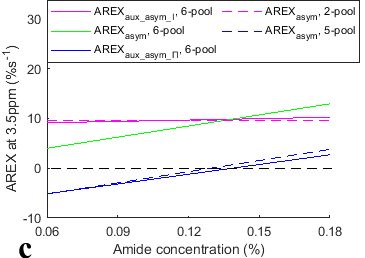

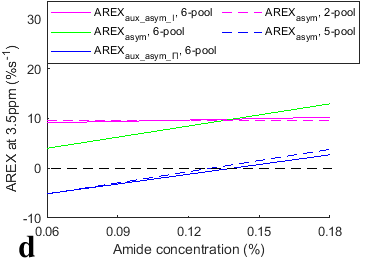

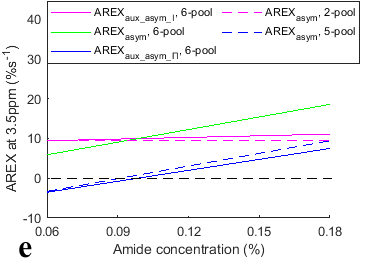

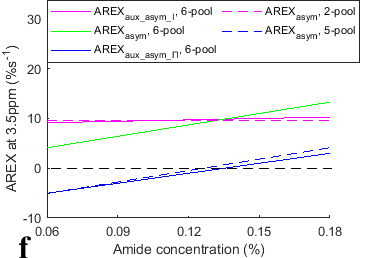
**

**Supporting information Fig S4.** Six-pool (amide, amine, guanidine, NOE, MT, and water) model simulated ${AREX}_{aux\_asym\_І}$_,_ ${AREX}_{asym}$, and ${AREX}_{aux\_asym\_\Pi}$ values at 3.5ppm (solid), and the two-pool (amine and water) and five-pool (amide, guanidine, NOE, MT, and water) model simulated ${AREX}_{asym}$ values at 3.5ppm (dashed), as a function of the amide concentration with amide k_sw_ of 40s^-1^ (a), 80s^-1^ (c), and 120s^-1^ (e), and amide T_2s_ of 2ms (b), 4ms (d), and 6ms (f). $B_{1\_L}= 2\mu T$ and $B_{1\_H}= 6\mu T$. The amide pool concentration was gradually increased to cause different relative size of APT to other effects. Notably, the differences between the six-pool model simulated ${AREX}_{aux\_asym\_І}$ and the two-pool model simulated ${AREX}_{asym}$, as well as between the six-pool model simulated ${AREX}_{aux\_asym\_\Pi}$ and the five-pool model simulated ${AREX}_{asym}$, are much smaller than the six-pool model simulated ${AREX}_{aux\_asym\_І}$, indicating that these two auxiliary asymmetry analysis metrics can effectively separate the amine CEST effect from other effects. Furthermore, the increase in both ${AREX}_{asym}$ and ${AREX}_{aux\_asym\_\Pi}$ values at 3.5ppm corresponds with higher amide concentration is due to the increased APT effect. On the other hand, the ${AREX}_{aux\_asym\_І}$ values at 3.5ppm remain relatively stable as they are less affected by the APT effect. The black dashed lines represent the AREX values of 0%s^-1^.

**
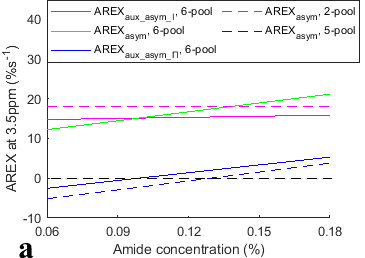

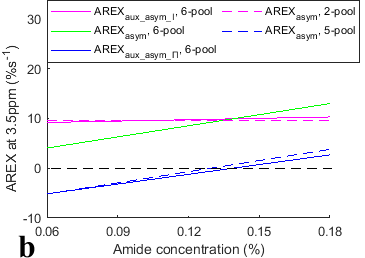

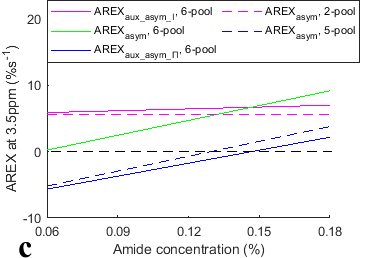

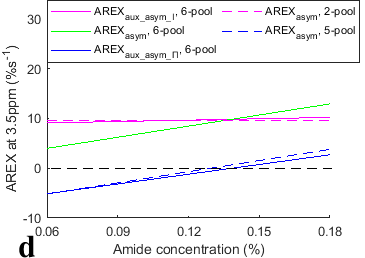
**

**Supporting information Fig S5.** Six-pool (amide, amine, guanidine, NOE, MT, and water) model simulated ${AREX}_{aux\_asym\_І}$_,_ ${AREX}_{asym}$, and ${AREX}_{aux\_asym\_\Pi}$ values at 3.5ppm (solid), and the two-pool (amine and water) and five-pool (amide, guanidine, NOE, MT, and water) model simulated ${AREX}_{asym}$ values at 3.5ppm (dashed), as a function of the amide concentration with amine k_sw_ of 3000s^-1^ (a) and 7000s^-1^ (c), and amine T_2s_ of 10ms (b) and 30ms (d). $B_{1\_L}= 2\mu T$ and $B_{1\_H}= 6\mu T$. The amide pool concentration was gradually increased to cause different relative size of APT to other effects. Notably, the differences between the six-pool model simulated ${AREX}_{aux\_asym\_І}$ and the two-pool model simulated ${AREX}_{asym}$, as well as between the six-pool model simulated ${AREX}_{aux\_asym\_\Pi}$ and the five-pool model simulated ${AREX}_{asym}$, are much smaller than the six-pool model simulated ${AREX}_{aux\_asym\_І}$, indicating that these two auxiliary asymmetry analysis metrics can effectively separate the amine CEST effect from other effects. Furthermore, the increase in both ${AREX}_{asym}$ and ${AREX}_{aux\_asym\_\Pi}$ values at 3.5ppm corresponds with higher amide concentration is due to the increased APT effect. On the other hand, the ${AREX}_{aux\_asym\_І}$ values at 3.5ppm remain relatively stable as they are less affected by the APT effect. The black dashed lines represent the AREX values of 0%s^-1^.

**
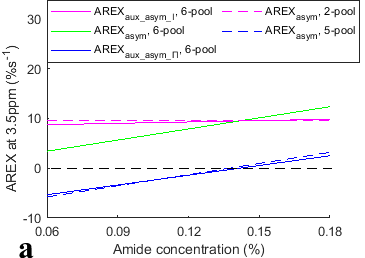

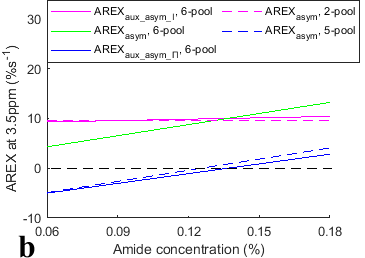

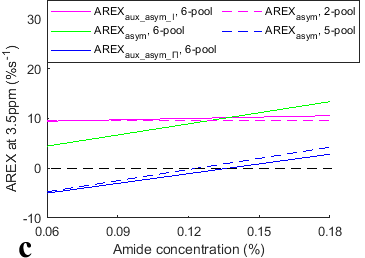

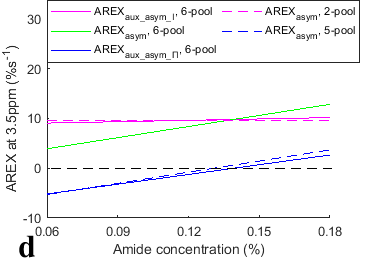
**

**Supporting information Fig S6.** Six-pool (amide, amine, guanidine, NOE, MT, and water) model simulated ${AREX}_{aux\_asym\_І}$_,_ ${AREX}_{asym}$, and ${AREX}_{aux\_asym\_\Pi}$ values at 3.5ppm (solid), and the two-pool (amine and water) and five-pool (amide, guanidine, NOE, MT, and water) model simulated ${AREX}_{asym}$ values at 3.5ppm (dashed), as a function of the amide concentration with guanidine k_sw_ of 200s^-1^ (a) and 800s^-1^ (c), and guanidine T_2s_ of 10ms (b) and 30ms (d). $B_{1\_L}= 2\mu T$ and $B_{1\_H}= 6\mu T$. The amide pool concentration was gradually increased to cause different relative size of APT to other effects. Notably, the differences between the six-pool model simulated ${AREX}_{aux\_asym\_І}$ and the two-pool model simulated ${AREX}_{asym}$, as well as between the six-pool model simulated ${AREX}_{aux\_asym\_\Pi}$ and the five-pool model simulated ${AREX}_{asym}$, are much smaller than the six-pool model simulated ${AREX}_{aux\_asym\_І}$, indicating that these two auxiliary asymmetry analysis metrics can effectively separate the amine CEST effect from other effects. Furthermore, the increase in both ${AREX}_{asym}$ and ${AREX}_{aux\_asym\_\Pi}$ values at 3.5ppm corresponds with higher amide concentration is due to the increased APT effect. On the other hand, the ${AREX}_{aux\_asym\_І}$ values at 3.5ppm remain relatively stable as they are less affected by the APT effect. The black dashed lines represent the AREX values of 0%s^-1^.

**
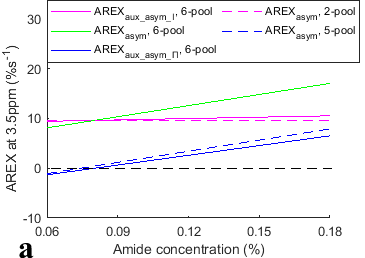

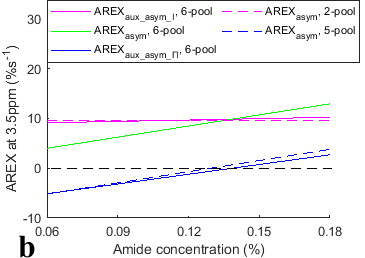

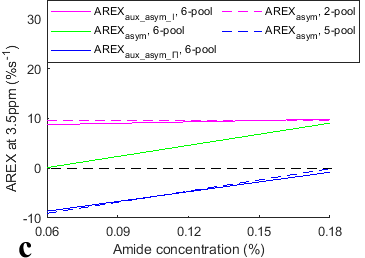

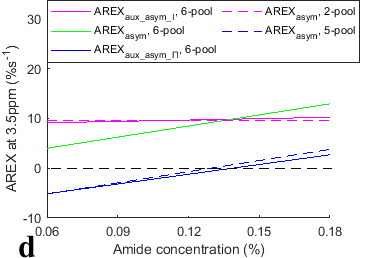
**

**Supporting information Fig S7.** Six-pool (amide, amine, guanidine, NOE, MT, and water) model simulated ${AREX}_{aux\_asym\_І}$_,_ ${AREX}_{asym}$, and ${AREX}_{aux\_asym\_\Pi}$ values at 3.5ppm (solid), and the two-pool (amine and water) and five-pool (amide, guanidine, NOE, MT, and water) model simulated ${AREX}_{asym}$ values at 3.5ppm (dashed), as a function of the amide concentration with NOE k_sw_ of 10s^-1^ (a) and 30s^-1^ (c), and NOE T_2s_ of 0.5ms (b) and 0.9ms (d). $B_{1\_L}= 2\mu T$ and $B_{1\_H}= 6\mu T$. The amide pool concentration was gradually increased to cause different relative size of APT to other effects. Notably, the differences between the six-pool model simulated ${AREX}_{aux\_asym\_І}$ and the two-pool model simulated ${AREX}_{asym}$, as well as between the six-pool model simulated ${AREX}_{aux\_asym\_\Pi}$ and the five-pool model simulated ${AREX}_{asym}$, are much smaller than the six-pool model simulated ${AREX}_{aux\_asym\_І}$, indicating that these two auxiliary asymmetry analysis metrics can effectively separate the amine CEST effect from other effects. Furthermore, the increase in both ${AREX}_{asym}$ and ${AREX}_{aux\_asym\_\Pi}$ values at 3.5ppm corresponds with higher amide concentration is due to the increased APT effect. On the other hand, the ${AREX}_{aux\_asym\_І}$ values at 3.5ppm remain relatively stable as they are less affected by the APT effect. The black dashed lines represent the AREX values of 0%s^-1^.


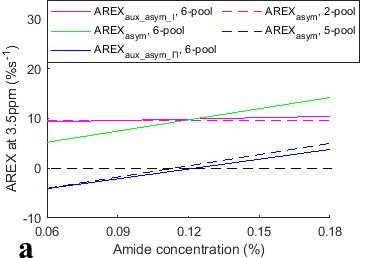

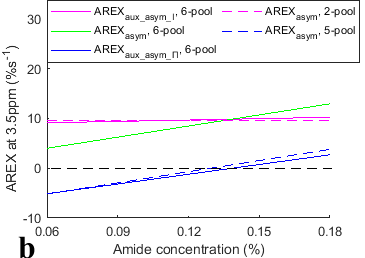

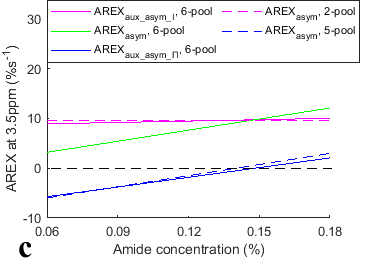

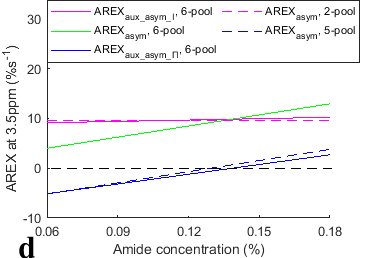


**Supporting information Fig S8.** Six-pool (amide, amine, guanidine, NOE, MT, and water) model simulated ${AREX}_{aux\_asym\_І}$_,_ ${AREX}_{asym}$, and ${AREX}_{aux\_asym\_\Pi}$ values at 3.5ppm (solid), and the two-pool (amine and water) and five-pool (amide, guanidine, NOE, MT, and water) model simulated ${AREX}_{asym}$ values at 3.5ppm (dashed), as a function of the amide concentration with MT k_sw_ of 15s^-1^ (a) and 35s^-1^ (c), and MT T_2s_ of 30µs (b) and 70µs (d). $B_{1\_L}= 2\mu T$ and $B_{1\_H}= 6\mu T$. The amide pool concentration was gradually increased to cause different relative size of APT to other effects. Notably, the differences between the six-pool model simulated ${AREX}_{aux\_asym\_І}$ and the two-pool model simulated ${AREX}_{asym}$, as well as between the six-pool model simulated ${AREX}_{aux\_asym\_\Pi}$ and the five-pool model simulated ${AREX}_{asym}$, are much smaller than the six-pool model simulated ${AREX}_{aux\_asym\_І}$, indicating that these two auxiliary asymmetry analysis metrics can effectively separate the amine CEST effect from other effects. Furthermore, the increase in both ${AREX}_{asym}$ and ${AREX}_{aux\_asym\_\Pi}$ values at 3.5ppm corresponds with higher amide concentration is due to the increased APT effect. On the other hand, the ${AREX}_{aux\_asym\_І}$ values at 3.5ppm remain relatively stable as they are less affected by the APT effect. The black dashed lines represent the AREX values of 0%s^-1^.

**
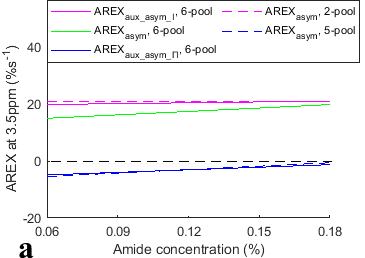

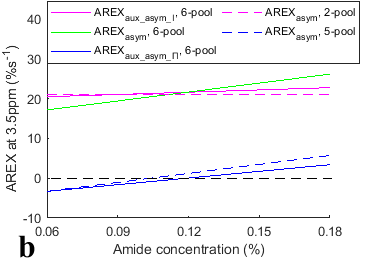

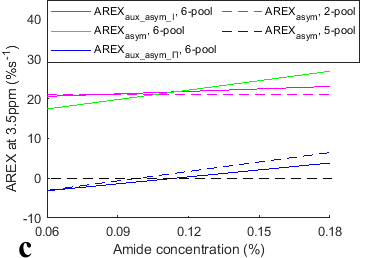

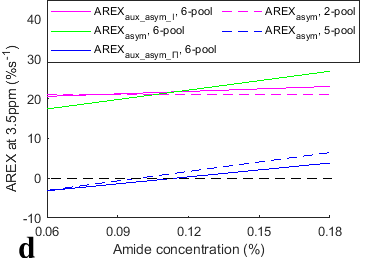

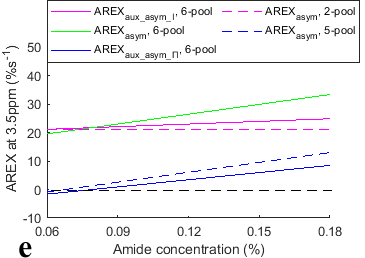

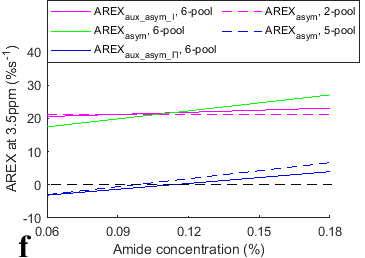
**

**Supporting information Fig S9.** Six-pool (amide, amine, guanidine, NOE, MT, and water) model simulated ${AREX}_{aux\_asym\_І}$_,_ ${AREX}_{asym}$, and ${AREX}_{aux\_asym\_\Pi}$ values at 3.5ppm (solid), and the two-pool (amine and water) and five-pool (amide, guanidine, NOE, MT, and water) model simulated ${AREX}_{asym}$ values at 3.5ppm (dashed), as a function of the amide concentration with amide k_sw_ of 40s^-1^ (a), 80s^-1^ (c), and 120s^-1^ (e), and amide T_2s_ of 2ms (b), 4ms (d), and 6ms (f). $B_{1\_L}= 3\mu T$ and $B_{1\_H}= 6\mu T$. The amide pool concentration was gradually increased to cause different relative size of APT to other effects. Notably, the differences between the six-pool model simulated ${AREX}_{aux\_asym\_І}$ and the two-pool model simulated ${AREX}_{asym}$, as well as between the six-pool model simulated ${AREX}_{aux\_asym\_\Pi}$ and the five-pool model simulated ${AREX}_{asym}$, are much smaller than the six-pool model simulated ${AREX}_{aux\_asym\_І}$, indicating that these two auxiliary asymmetry analysis metrics can effectively separate the amine CEST effect from other effects. Furthermore, the increase in both ${AREX}_{asym}$ and ${AREX}_{aux\_asym\_\Pi}$ values at 3.5ppm corresponds with higher amide concentration is due to the increased APT effect. On the other hand, the ${AREX}_{aux\_asym\_І}$ values at 3.5ppm remain relatively stable as they are less affected by the APT effect. The black dashed lines represent the AREX values of 0%s^-1^.


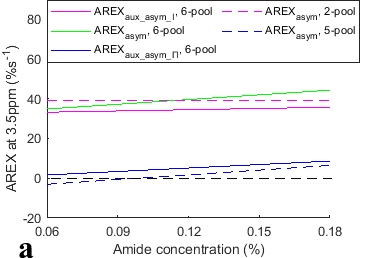

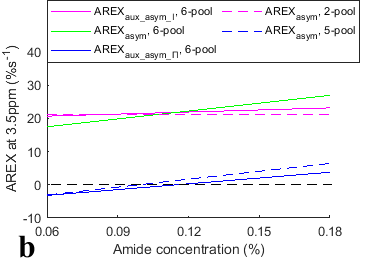

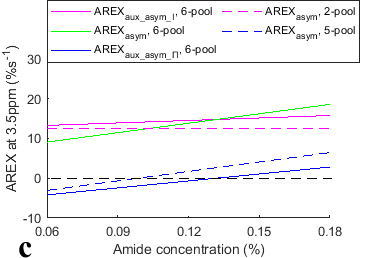

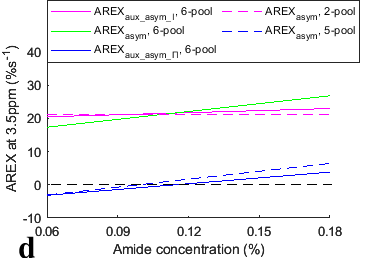


**Supporting information Fig S10.** Six-pool (amide, amine, guanidine, NOE, MT, and water) model simulated ${AREX}_{aux\_asym\_І}$_,_ ${AREX}_{asym}$, and ${AREX}_{aux\_asym\_\Pi}$ values at 3.5ppm (solid), and the two-pool (amine and water) and five-pool (amide, guanidine, NOE, MT, and water) model simulated ${AREX}_{asym}$ values at 3.5ppm (dashed), as a function of the amide concentration with amine k_sw_ of 3000s^-1^ (a) and 7000s^-1^ (c), and amine T_2s_ of 10ms (b) and 30ms (d). $B_{1\_L}= 3\mu T$ and $B_{1\_H}= 6\mu T$. The amide pool concentration was gradually increased to cause different relative size of APT to other effects. Notably, the differences between the six-pool model simulated ${AREX}_{aux\_asym\_І}$ and the two-pool model simulated ${AREX}_{asym}$, as well as between the six-pool model simulated ${AREX}_{aux\_asym\_\Pi}$ and the five-pool model simulated ${AREX}_{asym}$, are much smaller than the six-pool model simulated ${AREX}_{aux\_asym\_І}$, indicating that these two auxiliary asymmetry analysis metrics can effectively separate the amine CEST effect from other effects. Furthermore, the increase in both ${AREX}_{asym}$ and ${AREX}_{aux\_asym\_\Pi}$ values at 3.5ppm corresponds with higher amide concentration is due to the increased APT effect. On the other hand, the ${AREX}_{aux\_asym\_І}$ values at 3.5ppm remain relatively stable as they are less affected by the APT effect. The black dashed lines represent the AREX values of 0%s^-1^.

**
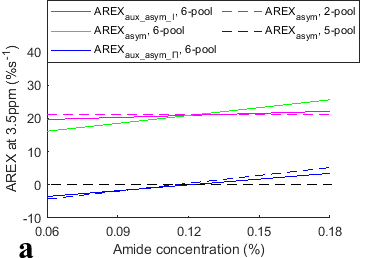

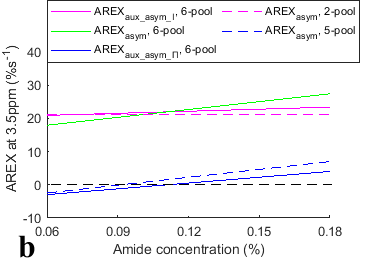

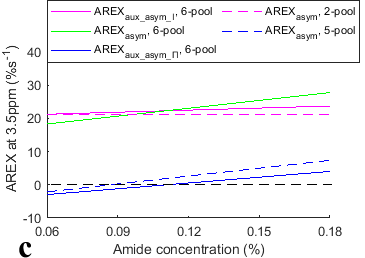

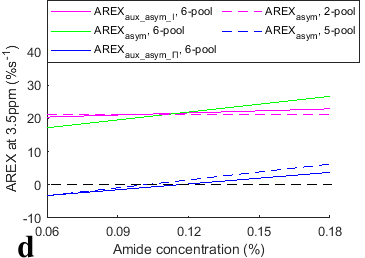
**

**Supporting information Fig S11.** Six-pool (amide, amine, guanidine, NOE, MT, and water) model simulated ${AREX}_{aux\_asym\_І}$_,_ ${AREX}_{asym}$, and ${AREX}_{aux\_asym\_\Pi}$ values at 3.5ppm (solid), and the two-pool (amine and water) and five-pool (amide, guanidine, NOE, MT, and water) model simulated ${AREX}_{asym}$ values at 3.5ppm (dashed), as a function of the amide concentration with guanidine k_sw_ of 200s^-1^ (a) and 800s^-1^ (c), and guanidine T_2s_ of 10ms (b) and 30ms (d). $B_{1\_L}= 3\mu T$ and $B_{1\_H}= 6\mu T$. The amide pool concentration was gradually increased to cause different relative size of APT to other effects. Notably, the differences between the six-pool model simulated ${AREX}_{aux\_asym\_І}$ and the two-pool model simulated ${AREX}_{asym}$, as well as between the six-pool model simulated ${AREX}_{aux\_asym\_\Pi}$ and the five-pool model simulated ${AREX}_{asym}$, are much smaller than the six-pool model simulated ${AREX}_{aux\_asym\_І}$, indicating that these two auxiliary asymmetry analysis metrics can effectively separate the amine CEST effect from other effects. Furthermore, the increase in both ${AREX}_{asym}$ and ${AREX}_{aux\_asym\_\Pi}$ values at 3.5ppm corresponds with higher amide concentration is due to the increased APT effect. On the other hand, the ${AREX}_{aux\_asym\_І}$ values at 3.5ppm remain relatively stable as they are less affected by the APT effect. The black dashed lines represent the AREX values of 0%s^-1^.

**
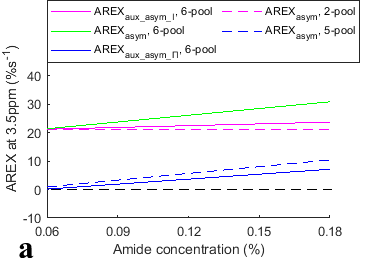

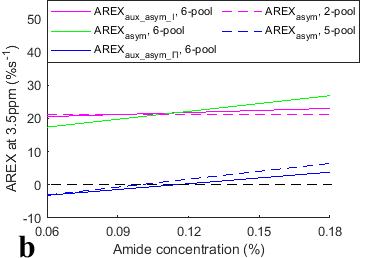

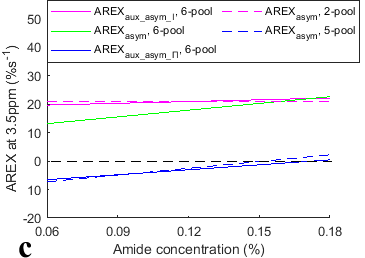

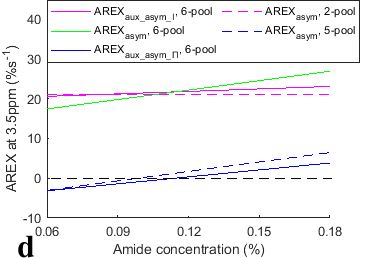
**

**Supporting information Fig S12.** Six-pool (amide, amine, guanidine, NOE, MT, and water) model simulated ${AREX}_{aux\_asym\_І}$_,_ ${AREX}_{asym}$, and ${AREX}_{aux\_asym\_\Pi}$ values at 3.5ppm (solid), and the two-pool (amine and water) and five-pool (amide, guanidine, NOE, MT, and water) model simulated ${AREX}_{asym}$ values at 3.5ppm (dashed), as a function of the amide concentration with NOE k_sw_ of 10s^-1^ (a) and 30s^-1^ (c), and NOE T_2s_ of 0.5ms (b) and 0.9ms (d). $B_{1\_L}= 3\mu T$ and $B_{1\_H}= 6\mu T$. The amide pool concentration was gradually increased to cause different relative size of APT to other effects. Notably, the differences between the six-pool model simulated ${AREX}_{aux\_asym\_І}$ and the two-pool model simulated ${AREX}_{asym}$, as well as between the six-pool model simulated ${AREX}_{aux\_asym\_\Pi}$ and the five-pool model simulated ${AREX}_{asym}$, are much smaller than the six-pool model simulated ${AREX}_{aux\_asym\_І}$, indicating that these two auxiliary asymmetry analysis metrics can effectively separate the amine CEST effect from other effects. Furthermore, the increase in both ${AREX}_{asym}$ and ${AREX}_{aux\_asym\_\Pi}$ values at 3.5ppm corresponds with higher amide concentration is due to the increased APT effect. On the other hand, the ${AREX}_{aux\_asym\_І}$ values at 3.5ppm remain relatively stable as they are less affected by the APT effect. The black dashed lines represent the AREX values of 0%s^-1^.


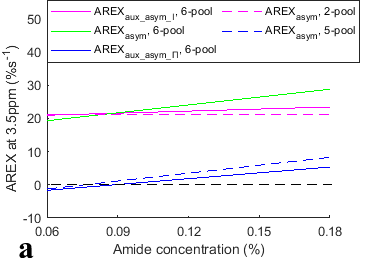

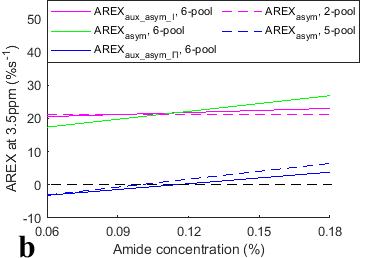

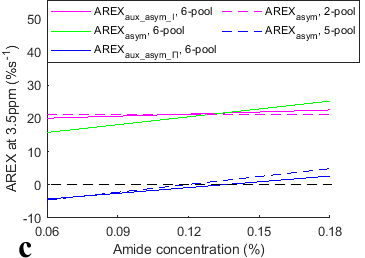

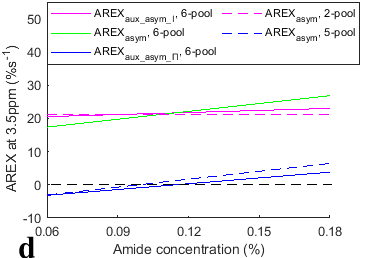


**Supporting information Fig S13.** Six-pool (amide, amine, guanidine, NOE, MT, and water) model simulated ${AREX}_{aux\_asym\_І}$_,_ ${AREX}_{asym}$, and ${AREX}_{aux\_asym\_\Pi}$ values at 3.5ppm (solid), and the two-pool (amine and water) and five-pool (amide, guanidine, NOE, MT, and water) model simulated ${AREX}_{asym}$ values at 3.5ppm (dashed), as a function of the amide concentration with MT k_sw_ of 15s^-1^ (a) and 35s^-1^ (c), and MT T_2s_ of 30µs (b) and 70µs (d). $B_{1\_L}= 3\mu T$ and $B_{1\_H}= 6\mu T$. The amide pool concentration was gradually increased to cause different relative size of APT to other effects. Notably, the differences between the six-pool model simulated ${AREX}_{aux\_asym\_І}$ and the two-pool model simulated ${AREX}_{asym}$, as well as between the six-pool model simulated ${AREX}_{aux\_asym\_\Pi}$ and the five-pool model simulated ${AREX}_{asym}$, are much smaller than the six-pool model simulated ${AREX}_{aux\_asym\_І}$, indicating that these two auxiliary asymmetry analysis metrics can effectively separate the amine CEST effect from other effects. Furthermore, the increase in both ${AREX}_{asym}$ and ${AREX}_{aux\_asym\_\Pi}$ values at 3.5ppm corresponds with higher amide concentration is due to the increased APT effect. On the other hand, the ${AREX}_{aux\_asym\_І}$ values at 3.5ppm remain relatively stable as they are less affected by the APT effect. The black dashed lines represent the AREX values of 0%s^-1^.

2µT 3µT

**
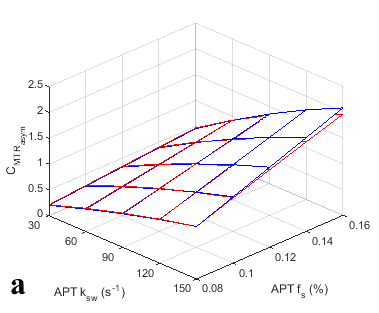

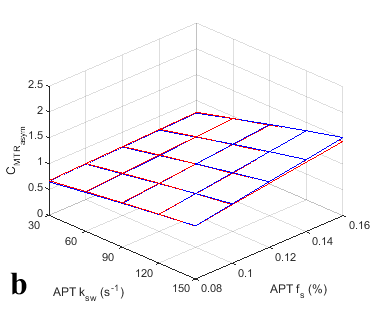

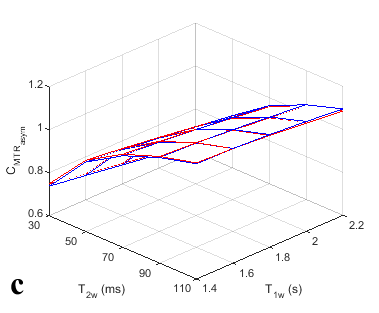

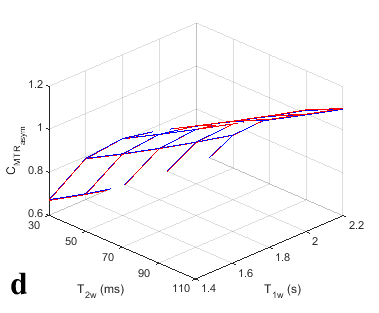

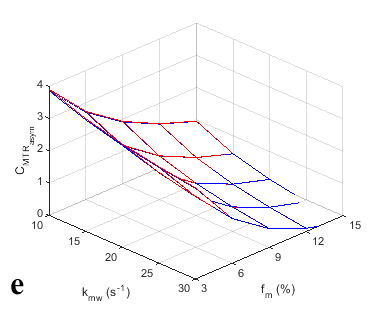

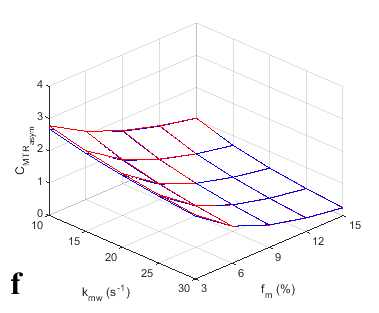
**

**
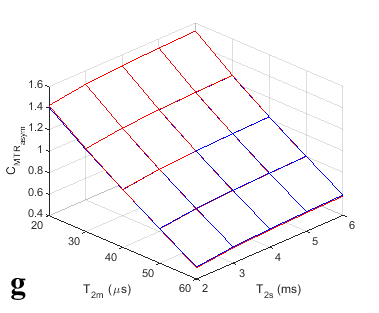

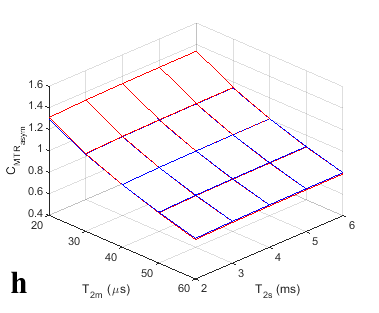
**

**Supporting information Figure S14.** Simulated $C_{{MTR}_{asym}}$ values at 3.5ppm (red) and calculated $C_{{MTR}_{asym}}$ values at 3.5ppm using Eq. (7) (blue) vs. APT f_s_ and APT k_sw_ (a, b), T_1w_ and T_2w_ (c, d), f_m_ and k_mw_ (e, f), as well as APT T_2s_ and T_2m_ (g, h) with B_1_ of 2µT (a, c, e, and g) and 3µT (b, d, f, and h), respectively. The NRMSE between the calculated and the simulated $C_{{MTR}_{asym}}$ values at 3.5ppm are 2.98%, 2.32%, 0.66%, 0.29%, 0.71%, 1.79%, 0.97%, 1.00%, respectively, in (a-h). The close alignment between the calculated and the simulated $C_{{MTR}_{asym}}$ values at 3.5ppm validates the approximate model in Eq. (7).

2µT 3µT

**
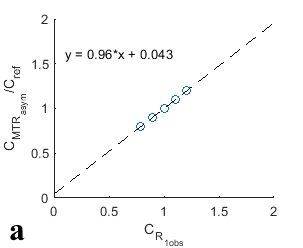

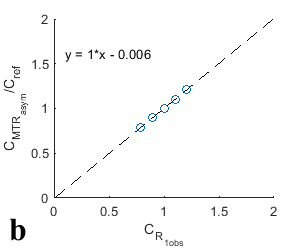

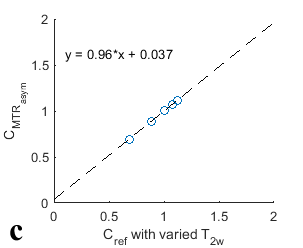

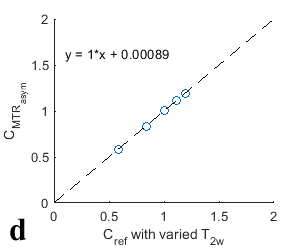

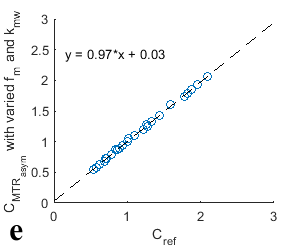

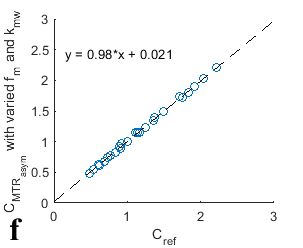

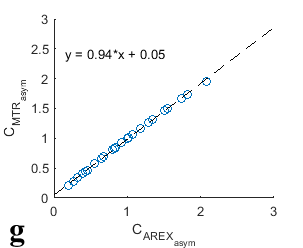

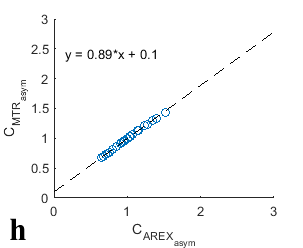
**

**Supporting information Figure S15.** Scatter plots between the simulated $C_{{MTR}_{asym}}/C_{ref}$ at 3.5ppm and $C_{R_{1obs}}$ (a, b), between the simulated $C_{{MTR}_{asym}}$ at 3.5ppm and $C_{ref}$ at 3.5ppm with varied T_2w_ (c, d), between the simulated $C_{{MTR}_{asym}}$ at 3.5ppm and $C_{ref}$ at 3.5ppm with varied f_m_ and k_mw_ (e, f), as well as between the simulated $C_{{MTR}_{asym}}$ at 3.5ppm and $C_{{AREX}_{asym}}$ at 3.5ppm (g, h) with B_1_ of 2µT (a, c, e, and g) and 3µT (b, d, f, and h), respectively. The dashed black lines represent the linear regression of all data points in each subfigure. This simulation suggests that the $C_{{MTR}_{asym}}$ at 3.5ppm has a roughly linear dependence on $C_{R_{1obs}}$, $C_{ref}$, and $C_{{AREX}_{asym}}$, respectively. Thus, $C_{{MTR}_{asym}}$ can be approximated as the multiplication of these three terms shown in Eq. (7). Data in (a, b) were from Supporting information Figure S14c and S14d with a series of T_1w_ and a constant T_2w_ of 70ms. Data in (c, d) were from Supporting information Figure S14c and S14d with a series of T_2w_ and a constant T_1w_ of 1.8s. Data in (e-f) were from Supporting information Figure S14e and S14f with a series of f_m_ and k_mw_. Data in (g-h) were from Supporting information Figure S14a and S14b with a series of f_s_ and k_sw_.

**
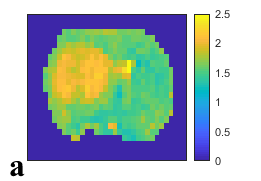

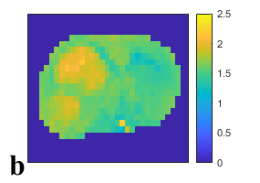

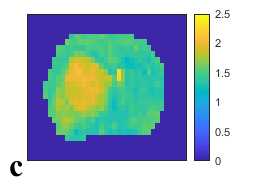

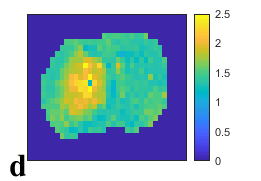

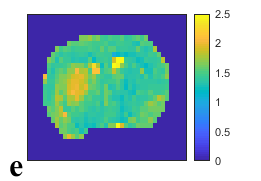
**

4.7T

**
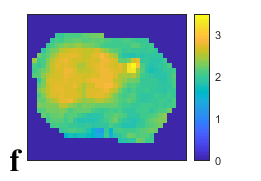

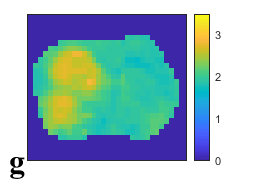

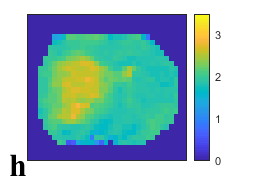

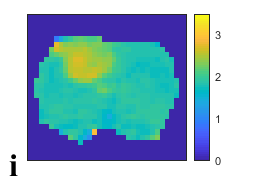
**

15.2T

**Supporting information Figure S16.** T_1obs_ maps from five rats acquired at 4.7T (a-e) and from four rats acquired at 15.2T (f-i). The T_1obs_ values are 1.9s, 1.8s, 1.9s, 1.9, and 1.7s in tumors, and 1.5s, 1.5s, 1.4s, 1.4, and 1.4s in normal tissues of the five rats acquired at 4.7T. The T_1obs_ values are 2.3s, 2.7s, 2.4s, and 2.3s in tumors, and 1.9s, 2.1s, 1.9s, and 1.9 in normal tissues of the four rats acquired at 15.2T. The rats depicted in (a) and (f), as well as in (b) and (g), are the same individuals, with images acquired at different scanners within a 24-h period. For each scanner, four Z-spectra with varying saturation field strengths and a corresponding T_1_ map were acquired, with the entire imaging session lasting approximately 1 hour. Due to the high mortality rate associated with prolonged scanning sessions, other rats scanned at 4.7T and 15.2T are different individuals.

**
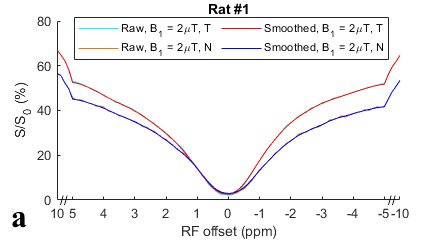

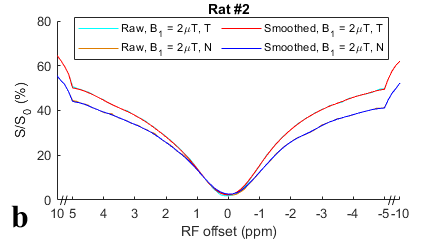

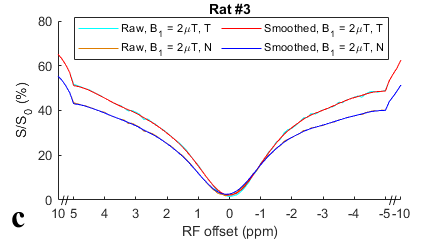
**

B_1_=2µT

B_1_=2µT

**
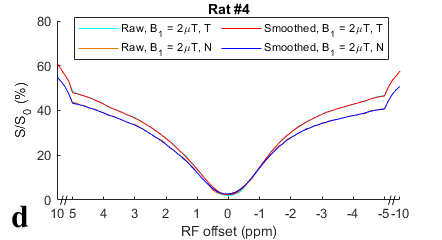

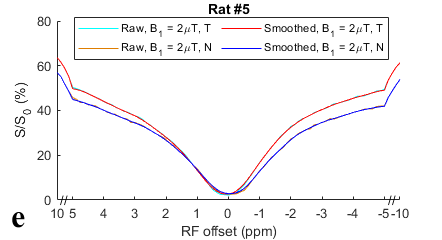

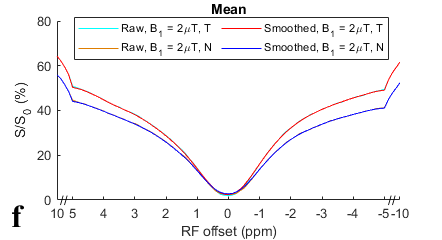
**

B_1_=3µT

**
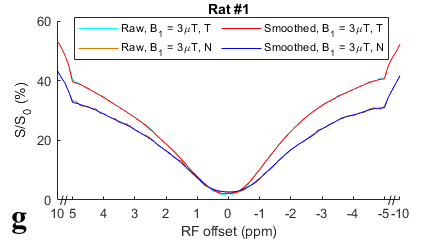

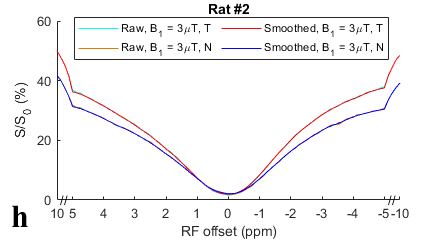

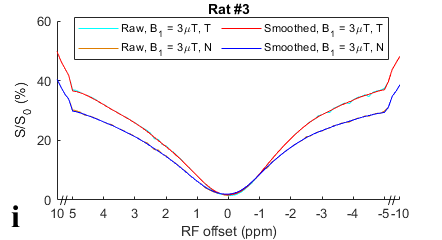
**

B_1_=3µT

**
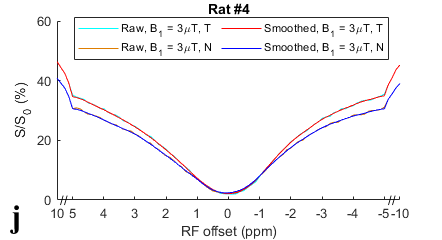

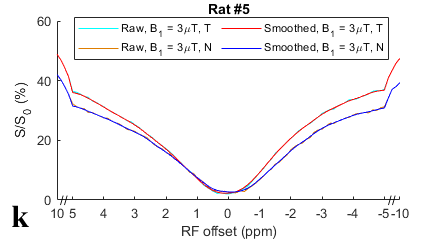
**

B_1_=6µT

B_1_=6µT

**Supporting information Figure S17.** Raw and smoothed CEST Z-spectra from the tumors (T) and contralateral normal tissues (N) in each rat brain as well as the mean of all brains with B_1_ of 2µT (a-f), 3µT (g-l), and 6µT (m-r), respectively, at 4.7T.

B_1_=2µT

B_1_=3µT

**Supporting information Figure S18.** MTR_asym_ (a, c) and AREX_asym_ (b, d) spectra from six-pool model simulations, along with the simulations without amine/guanidinium CEST, without NOE/asymmetric MT, and without APT, with B_1_ of 2µT (a, b) and 3µT (c, d). Notably, the APT effect at 3.5ppm overlays a sloping baseline on the six-pool model simulated MTR_asym_ and AREX_asym_ spectra. This sloping baseline decreases in simulations excluding the amine/guanidinium CEST, suggesting its origination from the amine/guanidinium CEST effects. Moreover, the negative values on the six-pool model simulated MTR_asym_ and AREX_asym_ spectra turn positive in the simulations without the NOE/asymmetric MT effects, indicating that these negative values are attributed to the NOE/asymmetric MT effects.

MTR_asym_  AREX_asym_  ΔMTR_asym_ ΔAREX_asym_

APT f_s_

T_1w_

T_2w_

f_m_

**Supporting information Figure S19.** MTR_asym_ (a, e, i, m), AREX_asym_ (b, f, j, n), ΔMTR_asym_ (c, g, k, o), and ΔAREX_asym_ (d, h, l, p) spectra from three-pool (amide, water, and MT) model simulations with varied APT f_s_ (a-d), T_1w_ (e-h), T_2w_ (i-l), or f_m_ (m-p), while other parameters remained constant. MT pool has no frequency offset. B_1_ is 2µT.

MTR_asym_  AREX_asym_  ΔMTR_asym_ ΔAREX_asym_

APT f_s_

T_1w_

T_2w_

f_m_

**Supporting information Figure S20.** MTR_asym_ (a, e, I, m), AREX_asym_ (b, f, j, n), ΔMTR_asym_ (c, g, k, o), and ΔAREX_asym_ (d, h, l, p) spectra from three-pool (amide, water, and MT) model simulations with varied APT f_s_ (a-d), T_1w_ (e-h), T_2w_ (i-l), or f_m_ (m-p), while other parameters remained constant. MT pool has no frequency offset. B_1_ is 3µT.

MTR_asym_  AREX_asym_  ΔMTR_asym_ ΔAREX_asym_

B_1_=2µT

B_1_=3µT

**Supporting information Figure S21.** MTR_asym_ (a, e), AREX_asym_ (b, f), ΔMTR_asym_ (c, g), and ΔAREX_asym_ (d, h) spectra from four-pool (amide, water, NOE(-3.5), and MT) model simulations with variation in both NOE(-3.5) f_s_ and MT f_m_, while other parameters remained constant. Asymmetric MT was set in this simulation. B_1_ are 2µT and 3µT.

B_1_=2µT

B_1_=3µT

**Supporting information Figure S22.** Relative change of the APT (a, c) and NOE (b, d) effects with variation in the solute T_2s_ values for a few solute k_sw_ values with B_1_ of 2µT (a, b) and 3µT (c, d). The APT and NOE effects were calculated using the formula $f_{s}k_{sw}\omega_{1}^{2}/(\omega_{1}^{2}+k_{sw}(R_{2s}+k_{sw}))$ which represents the AREX quantified CEST effects(13). The relative changes of the APT effect were calculated by subtracting the APT effect with an APT T_2_ of 4ms from those with other APT T_2_ values and normalizing by the APT effect with the APT T_2_ of 4ms. Similarly, the relative changes of the NOE effect were calculated by subtracting the NOE effect with a NOE T_2_ of 0.7ms from those with other NOE T_2_ values and normalizing by the NOE effect with the NOE T_2_ of 0.7ms. Other sample parameters are from Supporting information Table S4.

**Supporting information Figure S23.** MTR_asym_ values at 3.5ppm against saturation time, with B_1_ of 2µT from a variety of two-pool model simulations including amide and water (a), amine and water (b), guanidine and water (c), NOE and water (d), and MT and water (e), respectively. The simulations were conducted for a series of sample parameters for each pool. Dashed line indicates the saturation time of 2s. The ratios of MTR_asym_ values at 3.5ppm from each pool to that from APT (ratio_MTR_asym_), representing their relative contributions, for all combination of sample parameters were first calculated. The variation of this ratio with the saturation time of 2s (ratio_MTR_asym__2s) and 5s (ratio_MTR_asym__5s) was then calculated by the absolute value of (ratio_MTR_asym__2s - ratio_MTR_asym__5s) / ratio_MTR_asym__5s. The maximum variation of these ratios for all combination of sample parameters are 5.0% for amine, 3.0% for guanidine, 3.9% for NOE, and 15.4% for MT, respectively.

**Supporting information Figure S24.** MTR_asym_ values at 3.5ppm with B_1_ of 3µT from a variety of two-pool model simulations including amide and water (a), amine and water (b), guanidine and water (c), NOE(-3.5ppm) and water (d), and MT and water (e), respectively. The simulations were conducted for a series of sample parameters for each pool. Dashed line indicates the saturation time of 2s. The ratios of MTR_asym_ values at 3.5ppm from each pool to that from APT (ratio_MTR_asym_), representing their relative contributions, for all combination of sample parameters were first calculated. The variation of this ratio with the saturation time of 2s (ratio_MTR_asym__2s) and 5s (ratio_MTR_asym__5s) was then calculated by the absolute value of (ratio_MTR_asym__2s - ratio_MTR_asym__5s) / ratio_MTR_asym__5s. The maximum variation of these ratios for all combination of sample parameters are 3.9% for amine, 1.4% for guanidine, 2.1% for NOE, and 5.8% for MT, respectively**.**

**Supporting information Figure S25.** Averaged AREX_mfit_ spectra for APT, amine/guanidine CEST, and NOE effects from tumors (a) and contralateral normal tissues (b) in rat brain with B_1_ of 0.5µT, 1µT, 1.5µT, and 2µT at 15.2T. Mean and standard deviation of the AREX_mfit_-quantified amine/guanidine CEST effects at 3ppm, from tumor and normal tissues, as a function of B_1_ values. Guan is the abbreviation of guanidine.

1. Hua J, Jones CK, Blakeley J, Smith SA, van Zijl PCM, Zhou JY. Quantitative description of the asymmetry in magnetization transfer effects around the water resonance in the human brain. Magnetic Resonance In Medicine 2007;58(4):786-793.

2. Cui J, Zhao Y, Sun C, Xu J, Zu Z. Evaluation of contributors to amide proton transfer-weighted imaging and nuclear Overhauser enhancement-weighted imaging contrast in tumors at a high magnetic field. Magn Reson Med 2023;90(2):596-614.

3. Cohen O, Huang SN, McMahon MT, Rosen MS, Farrar CT. Rapid and quantitative chemical exchange saturation transfer (CEST) imaging with magnetic resonance fingerprinting (MRF). Magnetic Resonance In Medicine 2018;80(6):2449-2463.

4. Heo HY, Han Z, Jiang SS, Schar M, van Zijl PCM, Zhou JY. Quantifying amide proton exchange rate and concentration in chemical exchange saturation transfer imaging of the human brain. Neuroimage 2019;189:202-213.

5. Zhou J, Payen JF, Wilson DA, Traystman RJ, van Zijl PC. Using the amide proton signals of intracellular proteins and peptides to detect pH effects in MRI. Nature medicine 2003;9(8):1085-1090.

6. Cohen O, Otazo R. Global deep learning optimization of chemical exchange saturation transfer magnetic resonance fingerprinting acquisition schedule. NMR in biomedicine 2023;36(10):e4954.

7. Perlman O, Farrar CT, Heo HY. MR fingerprinting for semisolid magnetization transfer and chemical exchange saturation transfer quantification. NMR in biomedicine 2022.

8. Wermter FC, Bock C, Dreher W. Investigating GluCEST and its specificity for pH mapping at low temperatures. NMR in biomedicine 2015;28(11):1507-1517.

9. Cai KJ, Haris M, Singh A, Kogan F, Greenberg JH, Hariharan H, Detre JA, Reddy R. Magnetic resonance imaging of glutamate. Nature Medicine 2012;18(2):302-306.

10. Zhang ZQ, Wang KX, Park S, Li A, Li YG, Weiss RG, Xu JD. The exchange rate of creatine CEST in mouse brain. Magnetic Resonance In Medicine 2023;90(2):373-384.

11. Cai K, Haris M, Singh A, Kogan F, Greenberg JH, Hariharan H, Detre JA, Reddy R. Magnetic resonance imaging of glutamate. Nature medicine 2012;18(2):302-306.

12. Goerke S, Zaiss M, Bachert P. Characterization of creatine guanidinium proton exchange by water-exchange (WEX) spectroscopy for absolute-pH CEST imaging in vitro. NMR in biomedicine 2014;27(5):507-518.

13. Zaiss M, Zu ZL, Xu JZ, Schuenke P, Gochberg DF, Gore JC, Ladd ME, Bachert P. A combined analytical solution for chemical exchange saturation transfer and semi-solid magnetization transfer. NMR in biomedicine 2015;28(2):217-230.

14. Zu ZL. Towards the complex dependence of MTRasym on T-1w in amide proton transfer (APT) imaging. NMR in biomedicine 2018;31(7).
